# Trends in Burdens of Disease by Transmission Source (USA, 2005–2020) and Hazard Identification for Foods: Focus on Milkborne Disease

**DOI:** 10.1007/s44197-024-00216-6

**Published:** 2024-03-28

**Authors:** Michele M. Stephenson, Margaret E. Coleman, Nicholas A. Azzolina

**Affiliations:** 1 Syracuse University, Syracuse, NY 13203 USA; 2 Coleman Scientific Consulting, Groton, NY 13703 USA; 3 Independent consultant, Green Bay, WI 54313 USA

**Keywords:** Etiology, Interagency Food Safety Analytics Collaboration (IFSAC) food category, National Outbreak Reporting System (NORS), Food safety, Food security

## Abstract

**Background:**

Robust solutions to global, national, and regional burdens of communicable and non-communicable diseases, particularly related to diet, demand interdisciplinary or transdisciplinary collaborations to effectively inform risk analysis and policy decisions.

**Objective:**

U.S. outbreak data for 2005–2020 from all transmission sources were analyzed for trends in the burden of infectious disease and foodborne outbreaks.

**Methods:**

Outbreak data from 58 Microsoft Access^®^ data tables were structured using systematic queries and pivot tables for analysis by transmission source, pathogen, and date. Trends were examined using graphical representations, smoothing splines, Spearman’s rho rank correlations, and non-parametric testing for trend. Hazard Identification was conducted based on the number and severity of illnesses.

**Results:**

The evidence does not support increasing trends in the burden of infectious foodborne disease, though strongly increasing trends were observed for other transmission sources. Morbidity and mortality were dominated by person-to-person transmission; foodborne and other transmission sources accounted for small portions of the disease burden. Foods representing the greatest hazards associated with the four major foodborne bacterial diseases were identified. Fatal foodborne disease was dominated by fruits, vegetables, peanut butter, and pasteurized dairy.

**Conclusion:**

The available evidence conflicts with assumptions of zero risk for pasteurized milk and increasing trends in the burden of illness for raw milk. For future evidence-based risk management, transdisciplinary risk analysis methodologies are essential to balance both communicable and non-communicable diseases and both food safety and food security, considering scientific, sustainable, economic, cultural, social, and political factors to support health and wellness for humans and ecosystems.

**Supplementary Information:**

The online version contains supplementary material available at 10.1007/s44197-024-00216-6.

## Introduction

The global, national, and regional burdens of communicable (infectious) diseases and non-communicable diseases (NCDs) take a high toll on the health and well-being of humans and other organisms around the world (World Health Organization (WHO) [1]. Data on estimated primary causes of human deaths are reported by WHO, including estimates of 17 million deaths from NCDs worldwide in 2019, with 2.5 million of those deaths reported in the U.S. largely associated with NCDs and 0.4% of U.S. deaths (9955) attributed to diarrheal diseases in 2019.

Epidemiologic outbreak investigations, particularly those conducted under severe time and resource constraints, may focus narrowly on a few metrics for estimating the burden of disease or its severity (numbers and rates of morbidity, hospitalization, and mortality associated with outbreaks) rather than determining the root cause and interventions necessary to resolve outbreaks and prevent similar outbreaks in the future. A recent U.S. study [2] documented variable completeness of outbreak data reported by local, state, and territorial health departments to the federal Centers for Disease Control and Prevention (CDC) for entry into the National Outbreak Reporting System (NORS).

Zhang and colleagues [2] also pointed out that no documentation of data quality is available for CDC NORS data. As such, correlative associations based on such limited observational data with undocumented data quality would require additional data and analysis to establish causal associations essential to informing effective policies and improving public health accountability, as well as modeling trends in disease outbreaks with greater reliability, accounting for spatial and temporal dependencies in epidemiologic data [2–4].

Researchers around the world including those associated with Agencies of the European Union [5, 6], the international tripartite organizations [7], and an international expert committee [8] acknowledged the need for more holistic, multisector, and transdisciplinary collaborations, rather than narrow approaches focused within disciplinary silos. Such collaborations are consistent with One Health approaches [5–7, 9, 10] essential to supporting the transition to safe and sustainable food systems that promote human, animal, and ecosystem health in the twenty-first century.

These studies highlighted the need to replace reliance on often fragmented simplistic analyses based on limited data in traditional disciplinary silos (including epidemiology and microbiology) with transdisciplinary analysis, critical for realistic accounting for complex interacting systems required for reliable decision making. Separate disciplines alone are inadequate to identify and test alternative controls that optimize both food safety (incorporating data from epidemiology and microbiology) and food security (incorporating wider dimensions of access, agency, availability, stability, sustainability, and utilization [8] in complex regional, national, and global systems and ecosystems where political, economic, cultural, and other factors drive the status quo.

The practice of risk analysis described herein and in the work of others [11–14] can serve as a bridge to connect food safety and food security in a manner amenable to support decision making and improve health of humans and ecosystems. Notably, the WHO 75th World Health Assembly [15] identified the need to strengthen foodborne risk analysis (the ‘assessment, management, and communication of food risks’) to achieve sustainable health and food systems, reduce global health threats, and improve ecosystem management.

Epidemiologic and microbiological data are inputs to methods for assessments for foodborne pathogen risk (often quantitative microbial risk assessments or QMRAs). QMRAs are commonly applied for communicable diseases attributed to foods, though often using oversimplified simulation models estimating potential risks for enteric pathogen-food pairs, with little context or acknowledgment of the interdependencies and ambiguities of the real world. Simulations can also provide estimates of confidence intervals for hypothesis testing and decision support of alternative interventions or policies in risk management [16, 17]. Further, recent papers extended QMRA methods to predict epidemic curves and identify potential root causes for more effective prevention of future outbreaks [4, 18]. Methods for benefit-risk assessment [19–21] or risk–risk tradeoffs [11, 12, 22] seem to be applied more rarely for foodborne risk analysis.

Recent studies documented factors other than epidemiologic or microbial evidence as drivers of policies related to food safety [8, 13, 14], including consolidation of food systems at industrial scales as a barrier to potential dietary and health benefits. Attention to diverse drivers of change reliant on not only scientific knowledge, but also policy shifts and governance, are essential to transforming food systems to improve resiliency and achieve the UN Sustainable Development Goal 2 (zero hunger) as laid out in the sustainable food system framework [8]. The complexity of food systems and multi-sector interdependencies depicted in this framework illustrate the potential of multi-sector collaborative work, including epidemiologists and risk analysts, to balance food safety and food security.

From the risk analysis arena, a recent transdisciplinary analysis of a food system by Duret and colleagues [11] analyzed three potentially conflicting objectives (food safety, food waste from spoilage and recalls of potentially low-risk foods, and economic losses associated with energy and recalls for low-risk foods). These researchers determined that setting the refrigerator thermostat at 4 °C was the best compromise to maximize food safety and minimize economic losses from food waste and energy use.

Further, recent risk analysis studies include the risk management analysis of Farber and colleagues [23] that documented policy and legislative differences for Canada, the EU, and the U.S. based on microbial ‘hazard’ versus ‘risk’. Canada and the EU permit the presence of the pathogen *Listeria monocytogene*s (the pathogen that can cause listeriosis) at levels up to 100 pathogens per mL or gram in foods not permitting growth, a ‘risk’ basis that reflects the extremely high pathogen numbers associated with illness, even for more susceptible immunocompromised persons [24]. In contrast, the U.S. FDA has a ‘zero-tolerance’ policy based in potential ‘hazard’ that does not adjust for human tolerance of low pathogen numbers, but considers a food containing even a single pathogen cell as adulterated and subject to recall and destruction, though ‘risk’ of human illness to consumers may actually be low. The Farber study indicated benefits to alternative sampling approaching for monitoring low-risk foods that do not support pathogen growth and contain low pathogen levels: more efficient use of industry and regulatory resources; preserving customer confidence; contributing to secure and sufficient food supplies; decreasing food waste; reducing negative environmental effects; and avoiding unnecessary costs of food recalls for low-risk foods. Similarly, the QMRA performed by Abe and colleagues [25] identified combinations of factors linked to listeriosis in pasteurized milk: high initial level of the pathogen in milk; less effective pasteurization; and extremely high pathogen growth at inappropriately high temperatures. Further, their work determined that the dose–response assessment (the model of the relationship between ingested dose of a pathogen and likelihood of illness) had the strongest relevance to illness.

Together, these risk analysis studies point out the limitations of application of a ‘hazard-based’ risk management, the ‘zero-tolerance’ system for *L. monocytogenes*, a ubiquitous pathogen of low infectivity, in terms of promoting a favorable balance of food safety and food security within the sustainable food system framework [8].

Based on our perception of the need for greater coherence across disciplinary silos for nuanced bodies of data on burdens of illness, microbial ecology, and root cause analysis for complex systems, the authors undertook a detailed trend analysis for all six transmission sources included in U.S. CDC NORS [26] for years 2005 through 2020, the most recent 16 years of data available at the time requested [27].

**Fig. 1:**
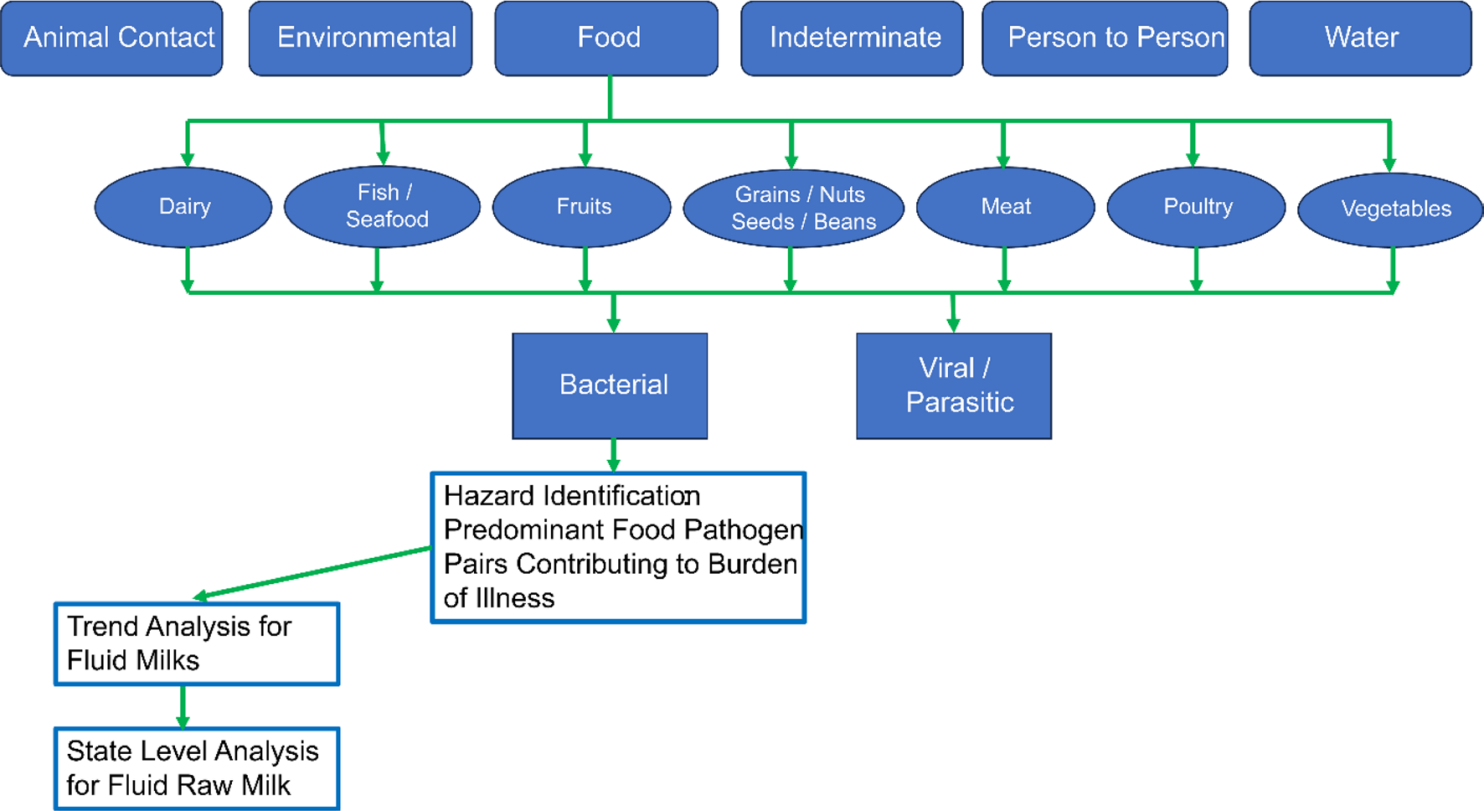
Process diagram for analysis of CDC NORS data by transmission source, food category, and etiology

Figure 1 provides an overview of our analytic approach for this CDC NORS dataset. The top row of text boxes in the figure depict the six transmission sources included in the dataset (animal contact; environmental; food; indeterminate; person-to-person; and water). The second row of text boxes depict the 7 major food categories included in the Interagency Food Safety Analytics Collaboration (IFSAC) system for which trends were considered. The third row of text boxes depict etiology (bacterial, viral, and parasitic) and trends by transmission source and food category. Next, for bacterial pathogens, Hazard Identification was conducted to identify the predominant food-pathogen pairs contributing to the burden of illness. For fluid milks, trend analysis was conducted, and for raw milk, state level analysis was conducted to account for state-level regulation of access to raw milk.

Our primary research objectives in analyzing this U.S. CDC NORS dataset were to:

1. determine trends for burdens of illness for all six transmission sources included in NORS;
2. begin the first step of QMRA, Hazard Identification, identifying predominant food-pathogen pairs contributing to the burden of bacterial foodborne disease; and
3. further explore trends in foodborne and milkborne illnesses relevant to risk analysis.

Due to the extent and complexity of the CDC NORS data for this 16-year period, we envision a series of manuscripts that provides a broader context for the foodborne burdens of infectious diseases and highlights different portions of the Microsoft Access^®^ dataset for foodborne hazards (particularly bacteria *Campylobacter*, pathogenic *E.coli* (Shiga Toxigenic *E. coli* or STECs), *L. monocytogenes*, and *Salmonella*) related to safety of food systems.

The European Union through the European Food Safety Authority [9] determines the strength of evidence for outbreaks in analyzing trends for foodborne illness and the top pathogen/food vehicle pairs in strong-evidence outbreaks that cause the highest numbers of outbreaks, illnesses, hospitalizations, and deaths. High uncertainty, questionable data quality, and ambiguous and conflicting studies merit multisector and transdisciplinary collaborations and more transparent deliberations of the available body of evidence, consistent with One Health [5–7, 10] and risk analysis [13, 28] principles discussed in more detail herein.

For risk analysis, high-quality data and methodology using transparent processes are essential to developing evidence-based decisions to balance food safety and food security. A recent analysis by Waller and colleagues [29] documented shortfalls in risk analysis quality for two government QMRAs, including apparent biases limiting the basis of knowledge, exclusion of conflicting expert opinions, and use of intentionally overpredictive assumptions (without demonstrating the impacts of alternative assumptions) that result in overestimated risk and underestimated uncertainty.

Given that evidence applied in QMRAs is typically incomplete, fragmented, and ambiguous, a lack of inter- or trans-disciplinary coherence in the knowledge base is, at best, misleading to regulators and consumers and, at worst, dangerous and likely to fail to appropriately balance transdisciplinary risks (e.g., economic, health, and ecosystem sustainability).

Simulations of possible risk scenarios that over-rely on unvalidated assumptions and fail to demonstrate the impact of alternative assumptions could be misleading, particularly without experimental validation of mitigation scenarios claimed to reduce risk or increase benefits. Recent research described the role of risk management as a ‘socially constructed,’ coherent, multidisciplinary, and anticipatory process of ‘sense-making,’ an ongoing and continuous process of making sense of reality [30].

Risk management as ‘sense making’ is based on beliefs about how retrospective knowledge illuminates plausible processes and constraints of complex social and physical interactions in order to reliably represent current knowledge of how the world functions [30]. Different conceptualizations of risk include: a techno-scientific focus, ‘assigning’ a probability of future events based on past events (perhaps along disciplinary silos); and constructionist perception, drawing inferences from incomplete, fragmented information, and discourse in order to balance differing ‘concerns, profits, safety, and reputation’. One improvement to global ‘sense-making’ might be expanding documentation for evidence quality, particularly when applied to trends (e.g., for strong-evidence outbreaks) [9], as well as for the allocation of funds for strengthening risk-based or evidence-based risk management.

The importance of transparency also extends to risk communication for epidemiologic and risk analyses. Engagement with diverse stakeholders (e.g., scientists, regulators, producers, processors, and consumers) is noted by Mogren and colleagues [31] as crucial to prevent additional outbreaks. Similarly, WHO [15] prioritized the action track for risk analysis, including risk communication. Transparent engagement with diverse stakeholders, including those who hold different cultural or behavioral values and ideologies or world views, is also crucial for high-quality risk analysis that includes evaluation of shortfalls, notably conflicting data and models, limitations for data and analysis, and incomplete characterization of uncertainty [32, 33]. Further, WHO [15] noted the need to advance a deeper understanding of linkages and drivers of foodborne illness.

While researchers [34] and risk practitioners [21, 35] acknowledge the need for simultaneous assessments of benefits and risks for consumers with diverse dietary preferences, some government agencies and public health authorities have focused more narrowly on risk, without considering benefits or risk–risk tradeoffs. Further, decision-makers may dismiss evidence of benefits and base policy on intentionally conservative assumptions and worst-case scenarios, ignoring or dismissing innate immunity and resistance to disease or severe disease for healthy people. Biased assumptions may intentionally or unintentionally overestimate risk and underestimate uncertainty for human health and wellness, as well as promote failure to discover and prevent unintended consequences that could have been identified by more comprehensive analysis.

In the U.S., deeper scrutiny of root cause analysis, data and analysis quality, strength of evidence determinations, and trends for strong epidemiologic evidence are merited. Regarding milkborne illness, Waller and colleagues [29] considered the U.S. QMRA [36] that reported both pasteurized and raw milk as high-risk foods for severe listeriosis yet divergent risk management positions consistent with pro-pasteurization bias. Also, lack of coherence in the body of evidence for raw and pasteurized milk outbreaks from the same source [27] over differing time periods, merits deeper assessment of conflicting studies [37–42], including the work described herein.

In addition to infectious disease burden, evidence regarding NCDs and the Right to Food Framework [8] merit deeper consideration in the US. Interest appears to be growing among consumers in the U.S. and around the world seeking access to unprocessed natural foods from local agriculture, including fresh, unprocessed (unhomogenized and unpasteurized) or raw milk [31, 43, 44] complete with its natural microbiota [45]. Regarding risk of allergy and asthma, Dietert and colleagues [21] documented evidence including a human provocation pilot study [46]that demonstrated increased risk of allergy and asthma, as well as respiratory and other infectious diseases, for pasteurized milk that may have been reduced or prevented had the natural microbiota of milk been present in consumed milk. A subsequent systematic review for North America documented a higher risk of hospitalization and death from listeriosis associated with pasteurized, not raw, milk [47]. Based on the need for greater coherence, the authors undertook a detailed trend analysis of milkborne illness from the NORS dataset.

The current work presented herein extends the analysis of NORS data from all transmission sources for the years 2009–2019 considered in the Wikswo study [48] for a longer period (2005–2020) and focuses on trends, with particular emphasis on the major food-pathogen pairs contributing to the burden of infectious disease, including *L. monocytogenes* not considered in the previous study.

Herein we address the data available, data quality, and gaps in knowledge regarding the root causes of foodborne morbidity and mortality for raw and pasteurized fluid milks. The data used in the current analysis includes CDC NORS data from outbreaks reported between 2005 and 2020 informing QMRA Hazard Identification, as well as U.S. Census data for this period in considering population-normalized trends.

Note that the analyses herein relate to major foodborne hazards for consideration in the first phase of QMRA (Hazard Identification) and are not risk estimates. Risk estimates for the hazards would be adjusted for asymmetries in consumption between foods and other factors (QMRA Exposure Assessment) and for relationships between ingested pathogen doses and strains likely to cause illness or severe illness (QMRA Dose–Response Assessment). Thus, these data represent ‘hazards’ that may cause human illness in the future but are not estimates of ‘risk’ (likelihood and severity of harm, with attendant uncertainty) that would be generated in QMRAs.

The current work utilizes graphical analysis, trend analysis, smoothing splines, and nonparametric rank-sum tests to test statistical hypotheses about potential root causes of morbidity and mortality. For milkborne illness, we describe trends for pasteurized and raw milk. Because U.S. states, not the federal government, regulate consumer access to raw milk, the potential relationships between legal access and raw milk-related outbreak, illness, and hospitalization rates are explored by state.

## Methods

### Epidemiological Data

The outbreak data used in this study were obtained from the CDC’s NORS database for the years 2005–2020 [27]. Each outbreak included a unique CDC identification number and provided the exposure state and the date of the first recorded illness. The counts of outbreaks, illnesses, hospitalizations, and deaths from all transmission sources (animal contact, environmental, foodborne, person-to-person, waterborne, and indeterminate/unknown), as well as etiology (identifying pathogenic microbes) were included in the analysis.

As depicted in Fig. 1, our initial analysis was for trends by transmission source using graphical analysis with smoothing splines. Charts were created by etiology from Microsoft Excel® pivot tables. Hazard Identification was conducted using Microsoft Excel^®^ pivot tables by food-pathogen pairs for morbidity as well as information about mortality. For foods, charts were created from Microsoft Excel® pivot tables using the Interagency Food Safety Analytics Collaboration (IFSAC) food categories for morbidity and mortality, with and without etiology. Duplicate etiologies for the same CDCID number were removed from counts of health outcome, though retained for summarizing data by etiology. A more detailed time series analysis was undertaken for raw fluid milk that contributed to the burden of foodborne illness using graphical analysis with Locally Weighted Scatterplot Smoothing (LOESS).

Further details on NORS data tables are provided in the Supplementary Materials, and additional information is available online (https://www.cdc.gov/nors/index.html).

### Data Preprocessing for Statistical Analysis

The NORS dataset for 2005–2020 included outbreaks attributed to both pasteurized and unpasteurized fluid milk, as well as processed and unprocessed dairy products. The primary question for evaluation herein was whether the burden of illness for each state and year (state-year) was a function of the legal status of fluid raw milk in that state-year, adjusting for the state population that year. Therefore, the analysis focused solely on outbreaks, illnesses, and hospitalizations associated with fluid raw milk, and both pasteurized and processed dairy products were excluded from the data table.

The data table for analysis was generated for fluid raw milk (Supplementary Information, Table S1) combining the raw milk-related outbreaks, illnesses, and hospitalizations, the U.S. Census populations for each state-year (see Sect. 2.3), the state legal classifications by state-year (see Sect. 2.4), and for some states, the reported numbers of licenses/permits issued to dairies approved to sell raw milk (see Sect. 2.4). The data table was then used as input for the statistical analysis. Adjusting for 4 multi-state outbreaks, a total of 162 outbreaks, 1696 illnesses, 170 hospitalizations, and two deaths were associated with raw milk from 2005 to 2020.

An additional data table (Supplementary Information, Table S2) combined information by state on legal classification, dairy commodities regulated, state Census data, and milking cow numbers and milk production from the Census of Agriculture.

Four of the 162 raw milk-associated outbreaks were multi-state outbreaks, where the same CDC identification number was attributed to more than one exposure state. These multi-state outbreaks were expanded to include each exposure state for the multi-state outbreak. For example, the 2005 CDC ID 257838 for whole raw milk occurred in both Oregon and Washington; therefore, this outbreak was assigned to both states in the data analysis. This approach mildly inflated the total number of outbreaks, taking four multi-state outbreaks and expanding them into eight different states, for a net increase of four outbreaks and a total of 166 outbreaks in the data analysis. However, this approach allowed the data analysis to evaluate state-level outbreaks more accurately. Multi-state cases were excluded from the data analysis of illnesses and hospitalizations since there was no mechanism for associating these counts with their respective exposure states.

### Population-Scaling

Population data for each state-year were obtained from the U.S. Census Bureau. Three U.S. Census Bureau tables were used, one for the population from 2000 to 2009 [49], one for the population from 2010 through 2019 [50], and one for the population in 2020 [51]. The population data for each state-year combination in the U.S. Census Bureau data were matched with each state-year combination in the outbreak data such that the data table contained population estimates for each outbreak record. The outbreak, illness, and hospitalization counts in each year were expressed as rates per one million persons, i.e., outbreaks/1MM, illnesses/1MM, and hospitalizations/1MM, respectively. The data table that is provided in the Supporting Information as Table S1 includes columns for state-year population and outbreak, illness, and hospitalization rates.

### Incorporating State Legal Availability

Supplementary Table 2 from Whitehead and Lake [41] provided some information about state legal availability for raw milk. For simplicity and consistency, the year in which a jurisdiction changed status was assigned the new status. In 2018, Whitehead and Lake [41] classified the legal status of raw milk into one of five groups (Table 1). The classifications for all states by state-year are provided in the Supplementary Information as Table S2.

**Table 1:** Classifications used for the legal availability of raw milk

R	Legal off-farm sales in retail stores, at farm markets, or both
F	Farm-gate sales are legal, but no off-farm sales
H	Herdshares are permitted by law or policy or no law prohibits herdshares
P	Farm-gate sales legal with “pet food” license
I	Both herdshares and other sales are illegal

The five classifications for the legal availability of raw milk were incorporated into the data analysis by re-expressing legal availability into a dichotomous variable of either “illegal” or “legal”. The data analysis used two binary definitions of illegal and legal: (Definition 1) illegal = I and legal = R, F, H, or P, and (Definition 2) illegal = I or P and legal = R, F, or H. In other words, for Definition 1, regardless of the type of legalization (off-farm, farm-gate, herdshare, or pet food), the legal availability of raw milk for human consumption was classified as “legal”. For Definition 2, the P classification was also considered illegal. The data table that is provided in the Supplementary Information as Table S1 includes columns for state legal classification and the binary definitions of illegal and legal under Definition 1 or Definition 2.

In addition to the legal classifications, seven states (California, Colorado, Maine, Massachusetts, New York, Texas, and Utah) responded to Freedom of Information Act (FOIA) requests for the number of new raw milk licenses (registrations) issued each year from 2005 to 2022. These numbers of licenses for each state-year were added to the data table for the seven states. The data table that is provided in the Supplementary Information as Table S1 includes a column for the number of state-year licenses; however, many of the cells are blank (missing data) due to our lack of information for 43 of the 50 states.

### Statistical Analysis

Graphical approaches were used to explore the dataset and to help guide further analysis. These graphical approaches included generating bar charts of outbreak, illness, or hospitalization rates versus time and grouped by state to help assess whether there was or was not visual evidence of increasing rates over time (Supplementary Information, Figures S1 through S3) or time-series plots of legal status (Figures S4 and S5) or the number of licenses issued (Fig. S6). In addition, smoothing splines, LOESS smoothers, and Spearman’s rho rank correlation coefficients were estimated for these data.

To quantitatively evaluate whether outbreak rates increased after a change in legal status, a nonparametric rank-sum test, which is sometimes called the "Wilcoxon Rank-Sum Test" or "Mann–Whitney Test" (hereafter "rank-sum test"), was used to compare the outbreak rates before and after a change in legal status [52]. The rank-sum testing could only be applied to eight states: Kentucky, Maryland, Michigan, Montana, North Dakota, Tennessee, West Virginia, and Wyoming, as the other states did not have a change in legal status or enough state-years where the legal classification was illegal and then switched to legal.

Due to few outbreaks in the eight states (only 16 outbreaks across eight states and 16 years) and therefore limited statistical power to distinguish outbreak rates between illegal and legal state-years, in addition to looking at eight individual states, all the outbreak rate data were combined by legal status to evaluate a larger, pooled dataset.

The rank-sum tests were conducted as one-sided tests, with the following null and alternative hypotheses:

H_0_ (null): Ƞ_1_ − Ƞ_2_ = 0.

H_A_ (alternative): Ƞ_1_ − Ƞ_2_ > 0.

Where: Ƞ_1_ is the median of outbreaks for “legal”, and Ƞ_2_ is the median of outbreaks for “illegal”.

In other words, the rank-sum tests asked whether the median outbreak rates observed when raw milk was legal were higher than those observed when raw milk was illegal.

## Results

### Burden of Disease and Disease Severity Across All Transmission Sources

Graphical depictions of the CDC NORS data from 2005 to 2020 are presented below.

**Fig. 2:**
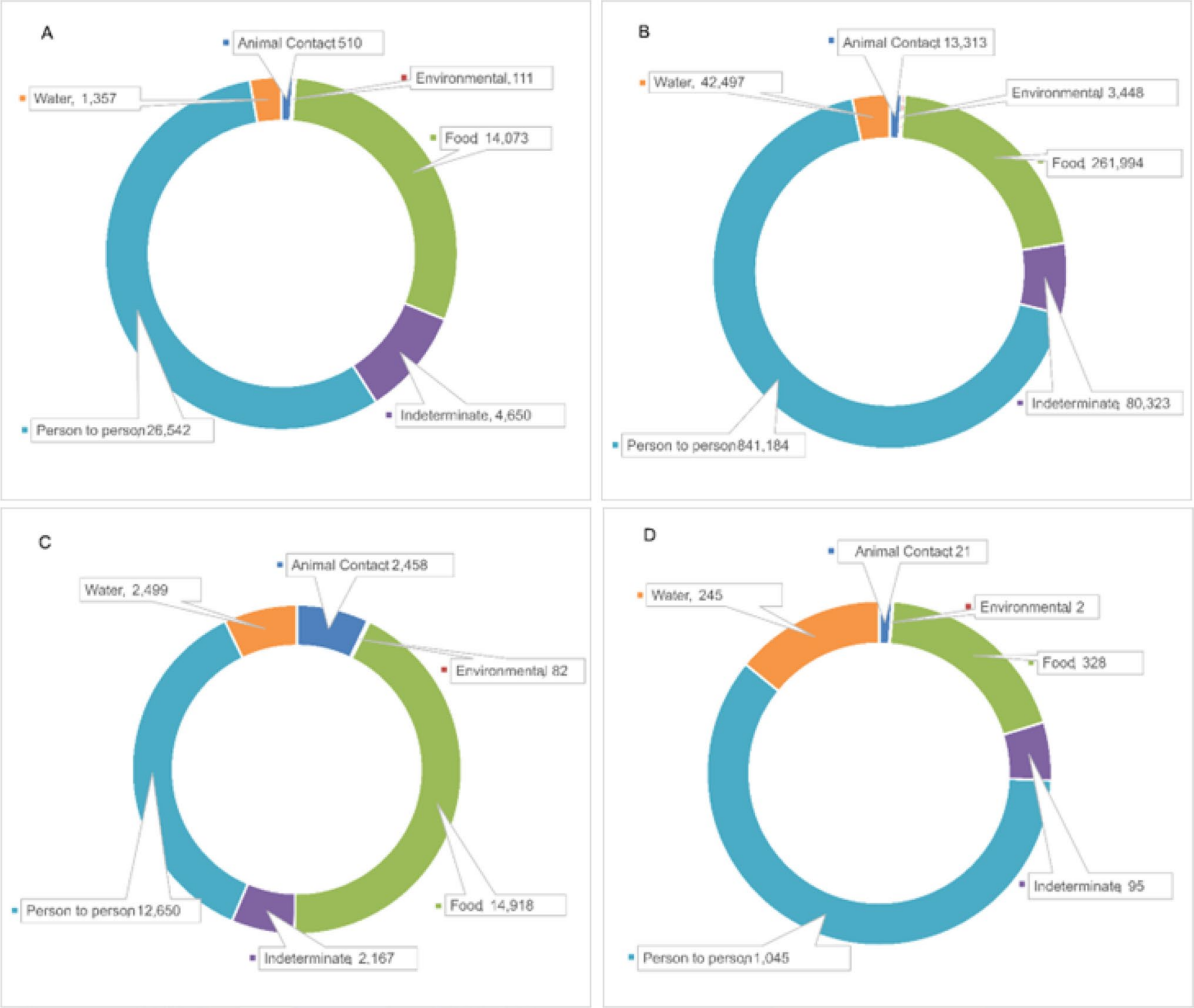
Numbers of U.S. outbreaks (**A** person-to-person 56%, foodborne 30%), illnesses (**B** person-to-person 68%, foodborne 21%), hospitalizations (**C** person-to-person 37%, foodborne 43%), and deaths (**D** person-to-person 60%, foodborne 19%) by modes of transmission (2005–2020) [27]

The major transmission source for outbreak data in this period was person-to-person transmission, accounting for 26,542 outbreaks (56% of all sources), 841,184 illnesses (68%), 12,650 hospitalizations (37%), and 1045 deaths (60%) (teal-colored portions of the sunburst graphs in Fig. 2). Foodborne transmission also accounted for substantial portions of the disease burden, including 14,073 outbreaks (30% of all sources), 261,994 illnesses (21%), 14,918 hospitalizations (43%), and 328 deaths (19%) (green-colored portions of the sunburst graphs in Fig. 2). Animal contact, environmental, indeterminate, and water sources accounted for 14% or less for all sources and all metrics.

**Fig. 3:**
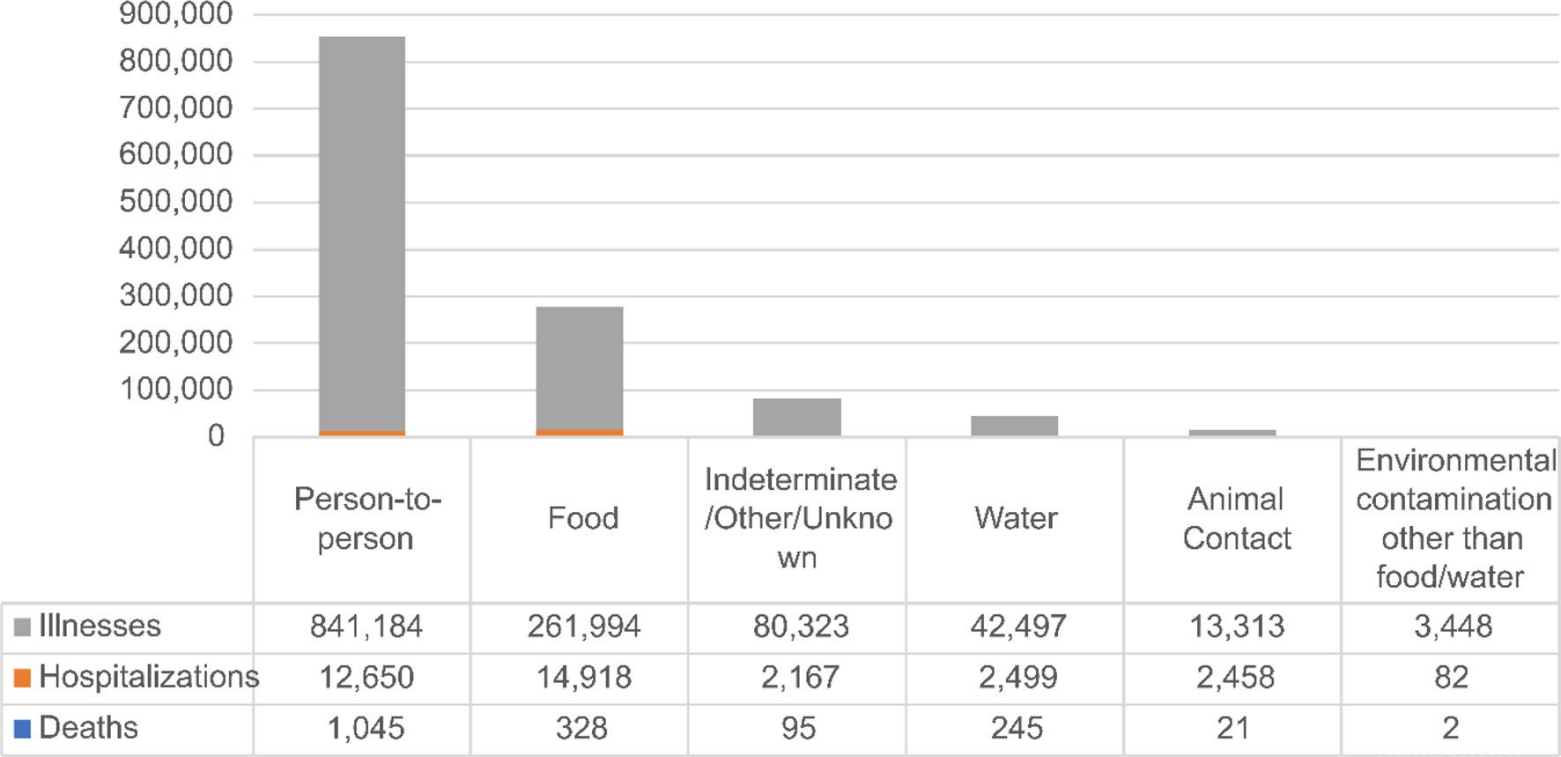
Numbers of illnesses, hospitalizations, and deaths by transmission source reported in the U.S. (2005–2020) [27]

Four primary transmission sources accounted for ~ 99% of the burden of illness: person-to-person (841,184 illnesses, 68% of total illnesses), food (261,994 illnesses, 21% total), indeterminate (80,323, 7%), and water (42,297, 3%) (Fig. 3). Person-to-person transmission also accounted for the highest numbers of illnesses (841,184) and deaths (1045), while foodborne transmission accounted for the highest numbers of hospitalizations (14,918). Deaths by transmission source were predominantly associated with Norovirus for person-to-person transmission, listeriosis and salmonellosis for foodborne transmission, Legionella for waterborne transmission, salmonellosis and STEC for animal contact, and one death each due to *Clostridium* and Norovirus for environmental transmission (Fig. 3).

**Fig. 4:**
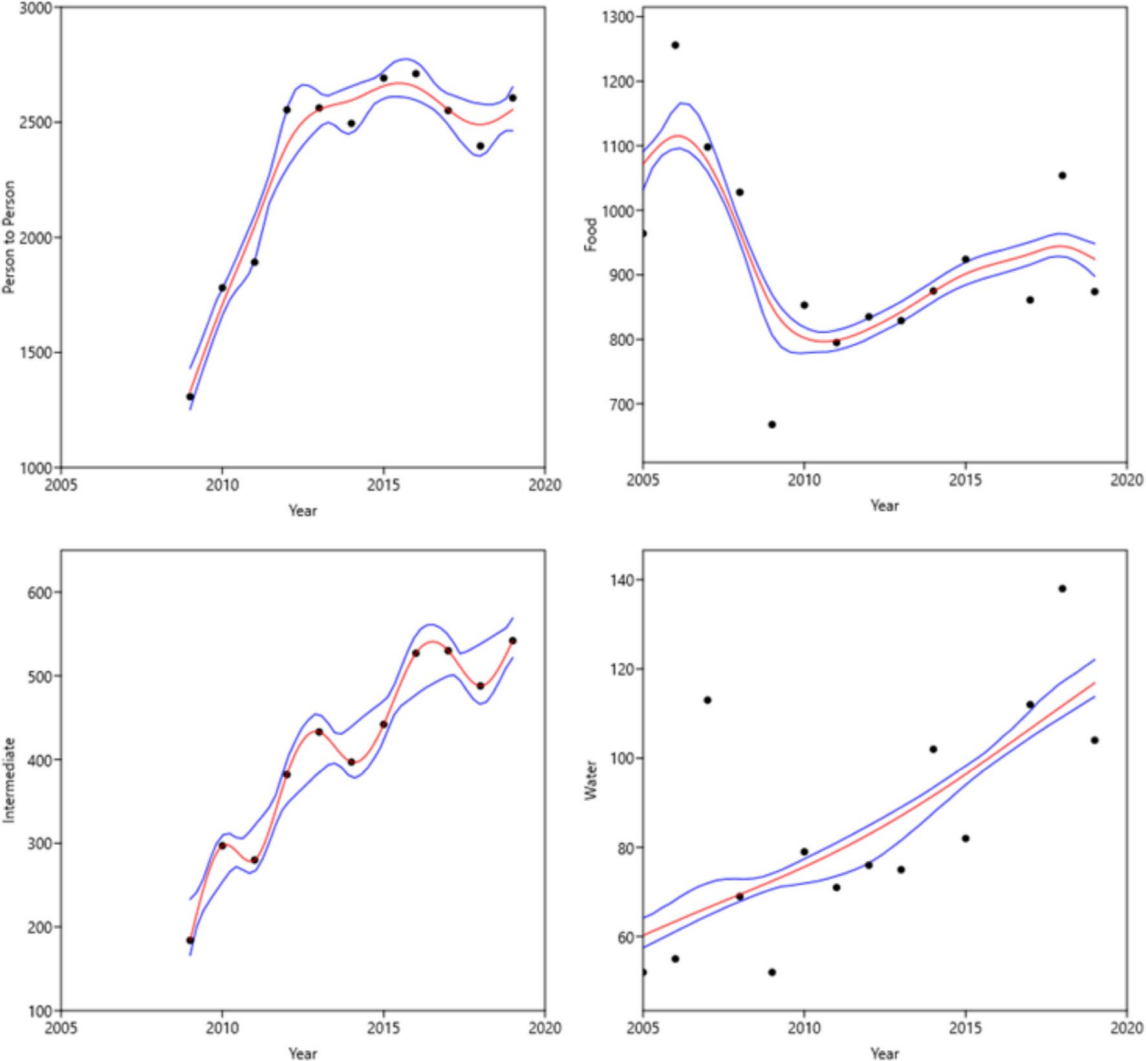
Trends in numbers of illnesses per year for major transmission sources using smoothing splines (red lines) and bootstrapped 95% confidence intervals (blue lines) (2005–2020) [27]

The trends for the four primary transmission sources representing ~ 99% of the burden of illness are depicted using smoothing splines (red lines) in Fig. 4. Visual and statistical assessment of data on the numbers of illnesses per year reveals no increasing trend for foodborne illness. Trends were strongly increasing initially for person-to-person followed by recent leveling off, and strongly increasing for indeterminate and water transmission (Fig. 4). We acknowledge that data on transmission by person-to-person, animal contact, and environmental sources were not reported in NORS before 2009. Therefore, the assessment of illness trends by those transmission sources reflects only the most recent 12 of 16 years of U.S. outbreak data.

**Fig. 5:**
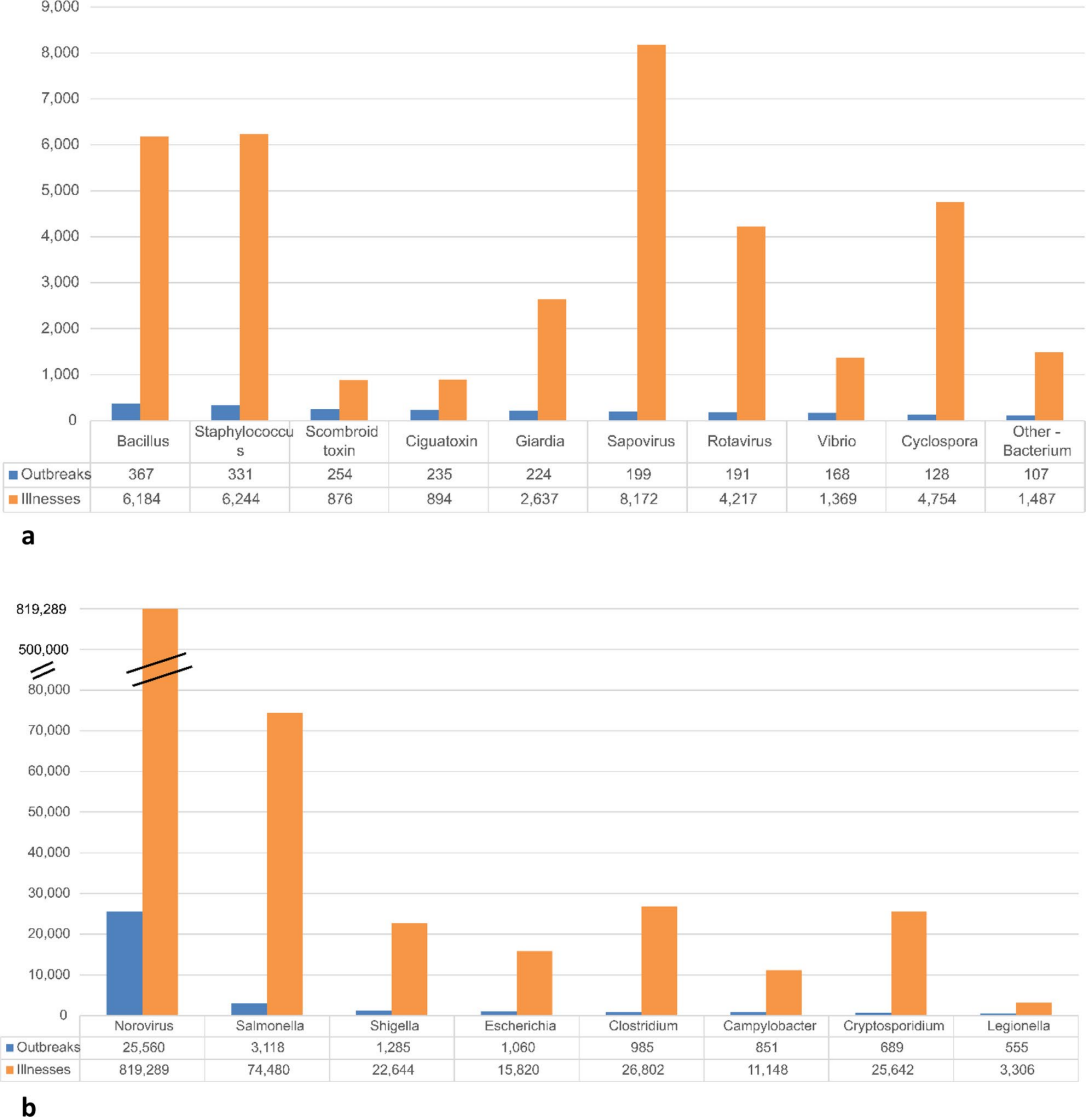
**a** Numbers of U.S. outbreaks and illnesses by pathogens or toxins across all transmission sources associated with less than 500 outbreaks (2005–2020) [27]. **b** Numbers of U.S. outbreaks and illnesses by pathogens or toxins across all transmission sources associated with more than 500 outbreaks (2005–2020) [27]

Considering the pathogens associated with outbreaks in this period, Norovirus accounted for the highest numbers of outbreaks (25,560) and illnesses (819,289) across all transmission sources (Fig. 5). In addition, Norovirus was also associated with the highest number of deaths by pathogen (954). Of these deaths, 887 (93%) were attributed to person-to-person transmission, and 15 deaths (1.2%) were attributed to foodborne transmission of Norovirus. The major bacterial pathogens contributing to morbidity were *Salmonella* spp., *Shigella* spp. (mainly waterborne), pathogenic *E. coli*, clostridia, and *Campylobacter* spp., and *Cryptosporidium* spp. was the major parasite causing morbidity (Fig. 5).

**Fig. 6:**
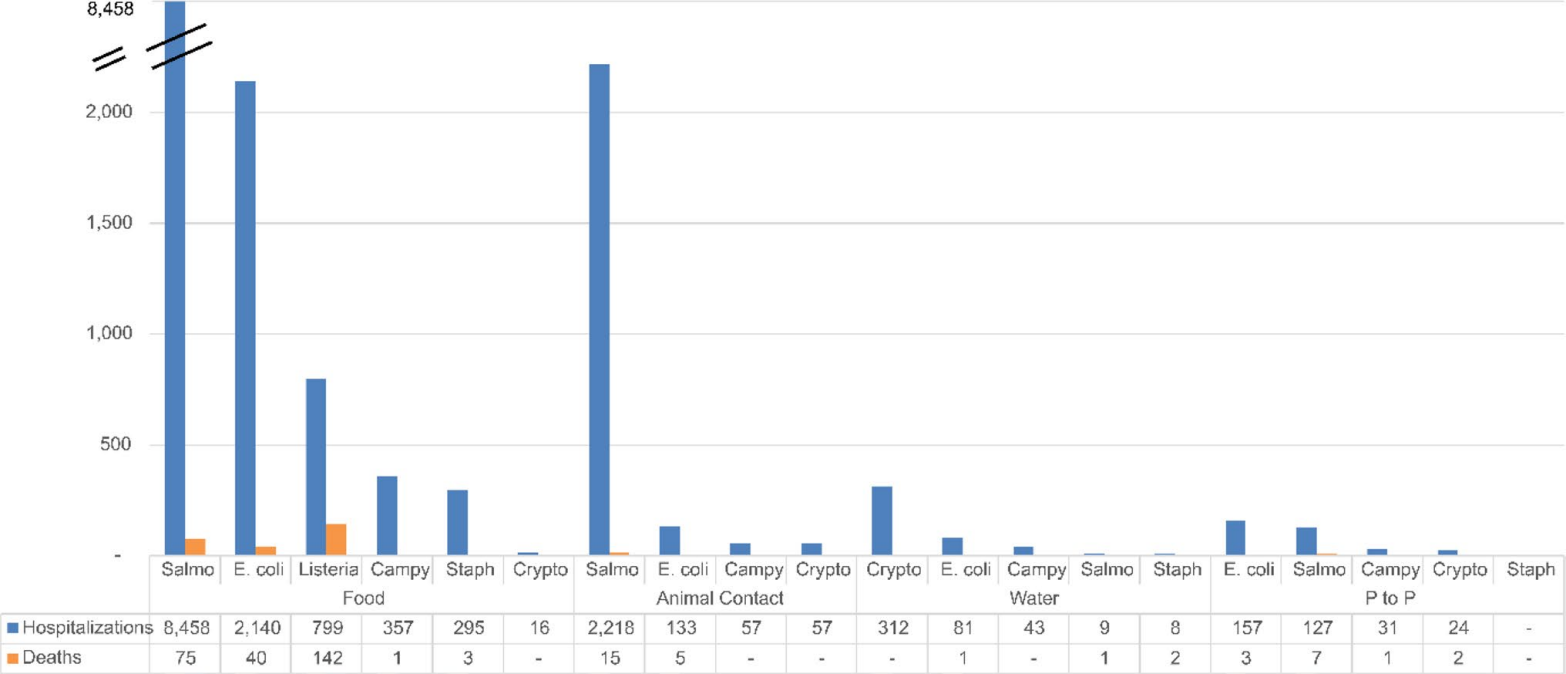
Numbers of hospitalizations and deaths by transmission source (P to P = person-to-person) for the top six bacterial and parasitic pathogens reported in the U.S. (2005–2020) [27]

Considering the severity of outbreaks associated with the major bacterial pathogens and parasites in this period, the highest number of hospitalizations was associated with *Salmonella* spp. (8458 from food among 11,349 hospitalizations over all transmission sources), and the highest number of deaths was associated with *L. monocytogenes* (142; Fig. 6), all from foodborne transmission.

### Burden of Foodborne Disease and Disease Severity for Foods

**Fig. 7:**
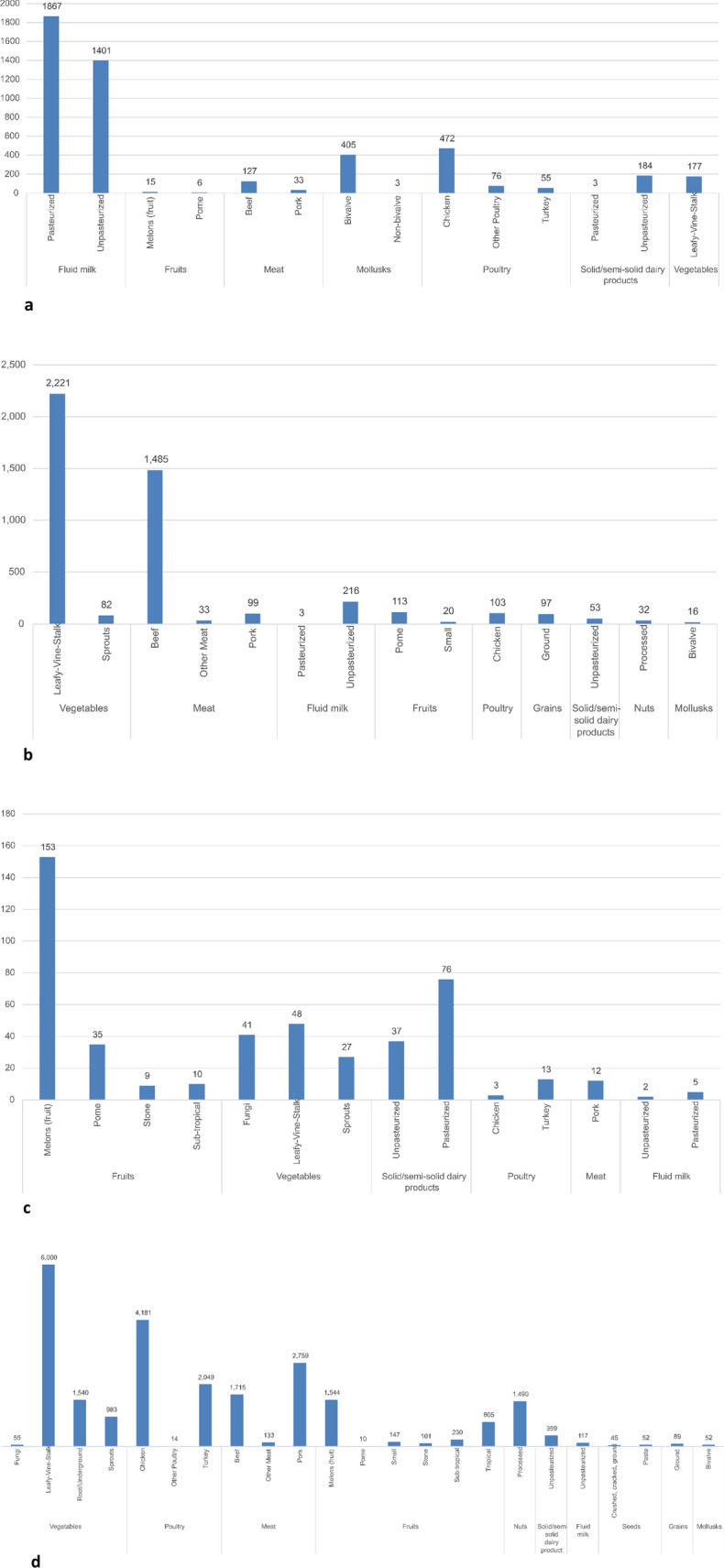
**a** Cases of foodborne illness: campylobacteriosis (2005–2020) [27]. **b** Cases of foodborne illness: pathogenic *E. coli* (2005–2020) [27]. **c** Cases of foodborne illness: listeriosis 2005–2020) [27]. **d** Cases of foodborne illness: salmonellosis (2005–2020) [27]. Note that an additional 24 salmonellosis cases and 1 hospitalization were associated with pasteurized milk in this period for outbreaks that were not coded with IFSAC food groupings

Morbidity data on numbers of illnesses associated with outbreaks that included IFSAC coding for foods or food categories associated with the four major foodborne bacterial hazards are provided in Fig. 7a–d below.

Campylobacteriosis cases were associated primarily with pasteurized milk, unpasteurized milk, chicken, bivalves, and leafy green vegetables (Fig. 7a). Cases for pathogenic *E. coli* were associated primarily with leafy/vine/stalk vegetables, beef, and raw milk (Fig. 7b). Listeriosis cases were associated primarily with melons, solid/semi-solid dairy products from pasteurized milks, and leafy/vine/stalk vegetables (Fig. 7c). Salmonellosis cases were associated primarily with leafy/vine/stalk vegetables, chicken, pork, turkey, beef, melons, and nuts (Fig. 7d).

**Fig. 8:**
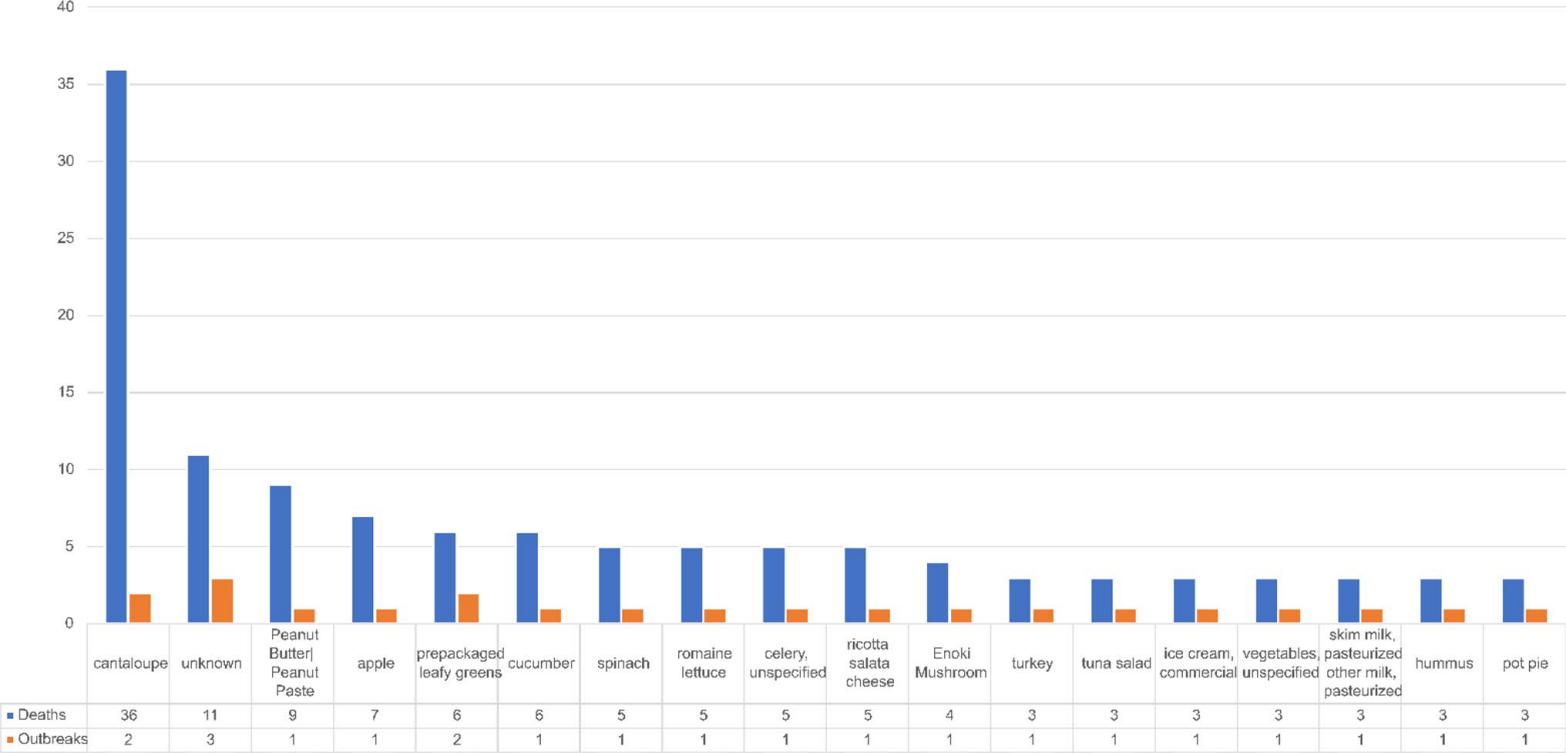
Foods associated with U.S. outbreaks reporting more than 2 deaths (2005–2020) [27]

Regarding disease severity, 172 outbreaks were associated with 347 deaths in this period. Nearly 90% of outbreaks reporting mortality were associated with one or two deaths. The remaining 10% of foodborne outbreaks reporting deaths were associated with more than 3 and up to 36 deaths, predominantly associated with fruits, vegetables, and peanut butter, as well as pasteurized cheese, pasteurized fluid milk, and ice cream processed from pasteurized milk (Fig. 8).

### Food-Pathogen Pairs Informing Hazard Identification

Epidemiologic evidence is used to contextualize the burden of illness associated with different foods and food-pathogen pairs for the first element of QMRA, Hazard Identification. Table 2 depicts the primary food-pathogen pairs contributing to the U.S. disease burden of bacterial foodborne illness using the CDC data for 2005–2020 [27].

**Table 2:** Bacterial pathogen/food pairs contributing to the burden of foodborne illness in the US

Bacterial pathogen	Food	Number of illnesses reported (2005–2020)
*Campylobacter* spp.	Pasteurized fluid milk	1873
	Raw fluid milk	1570
	Poultry	603
	Mollusks	408
	Homemade raw cheese	126
	Cheese (unspecified or pasteurized)	18
*Listeria monocytogenes*	Melons	153
	Cheese (unspecified or pasteurized)	143
	Pome, stone, and sub-tropical fruits	54
	Deli meats	49
	Leafy-vine-stalk vegetables	48
	Fungi	41
	Raw cheese	37
	Ice cream from pasteurized milk	10
	Pasteurized milk	5
	Raw milk	(2 reported; consumption unconfirmed)
*Salmonella* (non-typhoidal)	Poultry	6244
	Leafy-vine-stalk vegetables	6000
	Pork	2759
	Beef	1715
	Melons	1544
	Root/underground vegetables	1540
	Processed nuts	1490
	Sprouts	983
	Raw cheese	301
	Cheese (unspecified or pasteurized)	181
	Raw milk	162
	Pasteurized milk	24
Pathogenic *E. coli* or STEC	Leafy-vine-stalk vegetables	2221
	Beef	1485
	Raw milk	267
	Cheese (unspecified or pasteurized)	135
	Raw cheese	15
	Pasteurized milk	3

Pasteurized milk in this period was also associated with 125 cases of yersiniosis, 32 cases associated with an unidentified bacterium (CDCID 19133 VA 2014; no hospitalizations or deaths), and 4 cases where the agent was not identified. In addition to the bacterial agents above, pasteurized milk was associated with 33 cases of Norovirus illness, and raw milk was associated with 35 cases of cryptosporidiosis (3 hospitalizations, no deaths).

Considering foodborne deaths in this period, 143 deaths were attributed to *L. monocytogenes*, 75 deaths from salmonellosis, and 40 deaths from pathogenic *E. coli*. Six deaths were associated with fluid milk in this period: one death each from campylobacteriosis and listeriosis attributed to raw milk; 3 listeriosis deaths and one yersiniosis death attributed to pasteurized milk.

### Legal Access to Raw Milks and Outbreaks by State

States regulate legal access to raw milk, though inter-state sale is prohibited by federal law in the US. Depicting data by state is thus of great importance in this study since states regulate raw milk access, not federal authorities. The raw data on numbers and rates of outbreaks, illnesses, hospitalizations, and deaths, as well as population estimates from U.S. Census data, are provided by state and year in Supplementary Table S1. Additional information on state laws for raw milk access, along with notes on changes in legal status for fluid raw milk and other raw dairy commodities, numbers of milking cows and milk production by state from the U.S. Census of Agriculture, and U.S. Census data by state is provided in Supplementary Table S2.

**Fig. 9:**
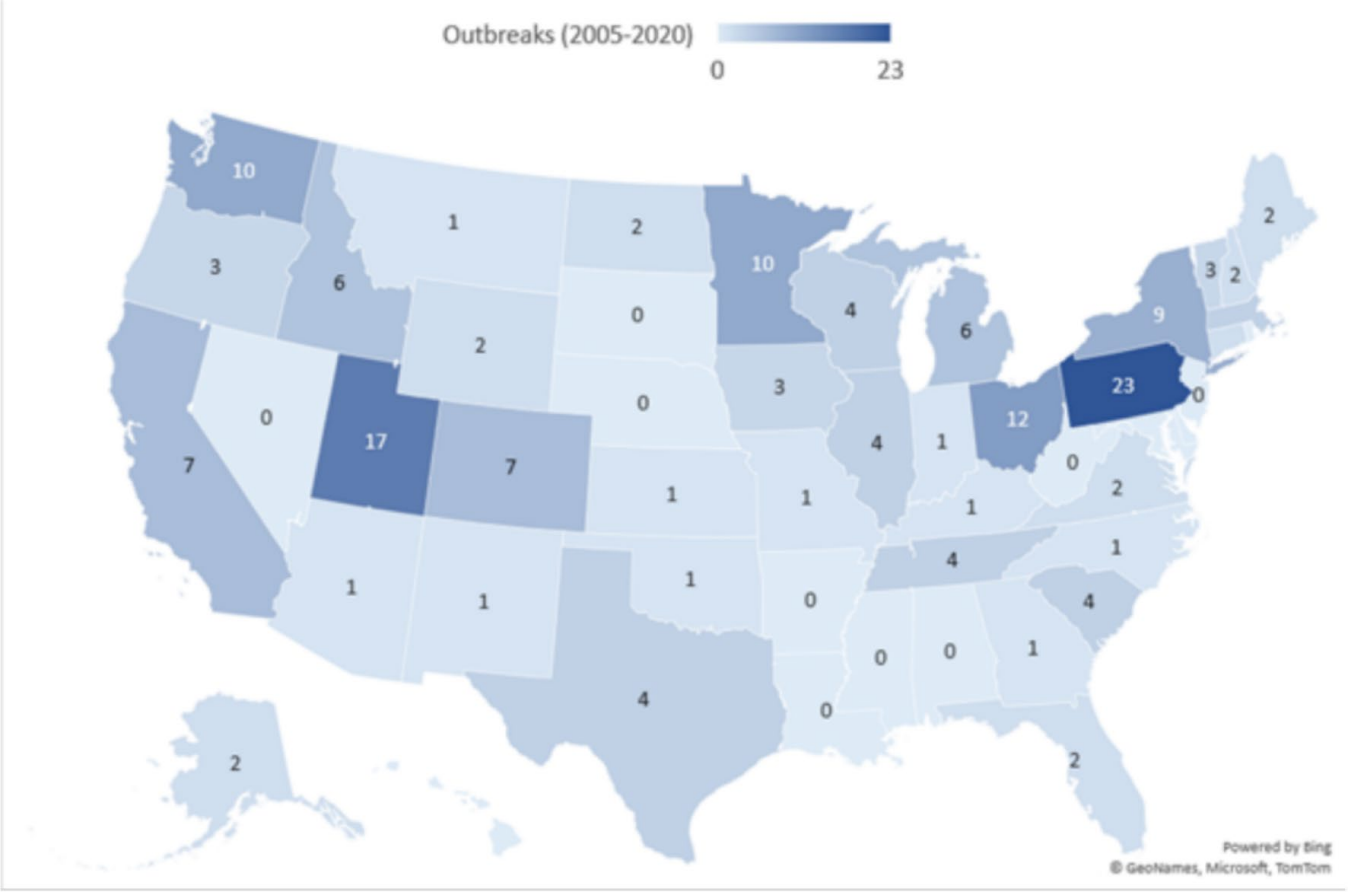
The total number of raw milk outbreaks by U.S. state reported from 2005 to 2020 [27]. Note that the map shows the results that expanded four multi-state outbreaks to their exposure states for a net increase of four outbreaks

The total number of raw milk outbreaks by U.S. state reported from 2005 to 2020 [27] included expansion for four multi-state outbreaks to each exposure state, for a net increase of four outbreaks. Fourteen states reported no raw milk outbreaks over the entire period: AL, AR, DC, DE, HI, LA, MD, MS, NE, NJ, NV, RI, SD, and WV. Three of these states permitted farm sales (AR, MS, SD), two either permitted herdshares or had no law prohibiting herdshare access (HI, WV), six permitted pet food sales (AL, DC, DE, LA, MD, NJ), and one permitted no legal access (NV). Ten states reported one raw milk outbreak, eight states had two outbreaks, 14 states had three to nine outbreaks, and the remaining five states had ≥ 10 outbreaks (Fig. 9).

The number of illnesses per raw milk per outbreak ranged from 2 to 163 illnesses, including 7 outbreaks exceeding 38 illnesses over this period. The number of hospitalizations was typically 0, and the maximum per year was 10.

### Trends for Burdens of Disease Associated with Fluid Milks

**Fig. 10:**
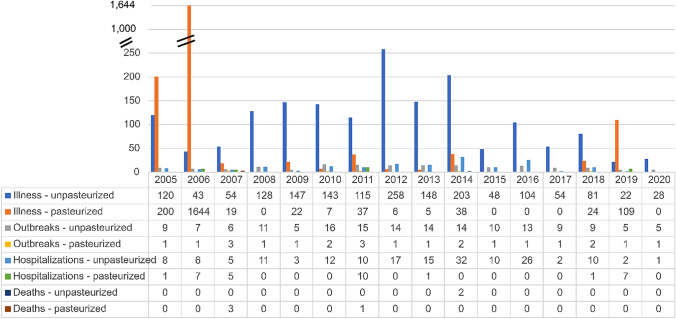
Numbers illnesses, outbreaks, hospitalizations, and deaths by year for unpasteurized (raw) and pasteurized milk (2005–2020) [27]

A total of 3,807 illnesses were reported for fluid milk. Both raw and pasteurized milk were associated with outbreaks, illnesses, hospitalizations, and deaths over the 16-year period, with raw milk associated with 162 outbreaks, 1,696 illnesses, 170 hospitalizations, and 2 deaths in 37 of 50 U.S. states, and pasteurized milk associated with 18 outbreaks, 2,111 illnesses, 32 hospitalizations, and 4 deaths (Fig. 10). Because deaths associated with fluid milk was so sparse (two deaths from raw milk and 4 deaths from pasteurized milk over the 16-year period), no statistical analysis on mortality rates for milk were conducted herein. We note documentation for the two deaths associated with raw milk consumption that were complicated by pre-existing underlying diseases that likely contributed to fatal outcomes [53, 54].

**Fig. 11:**
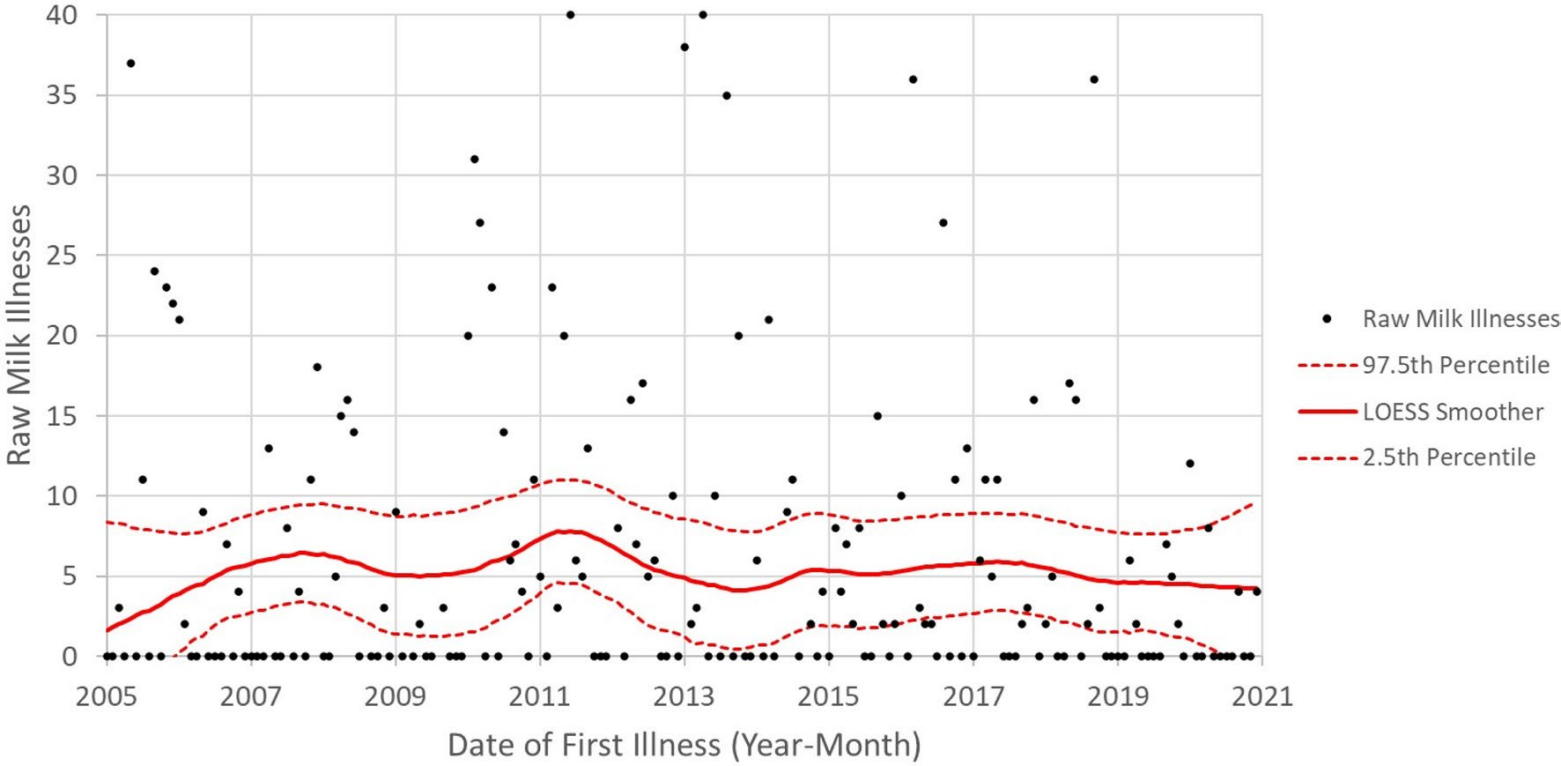
Numbers illnesses by date of first illness for raw milk (2005–2020) [27] depicted using LOESS Smother (red lines) and 95% confidence intervals (red dashed lines)

**Fig. 12:**
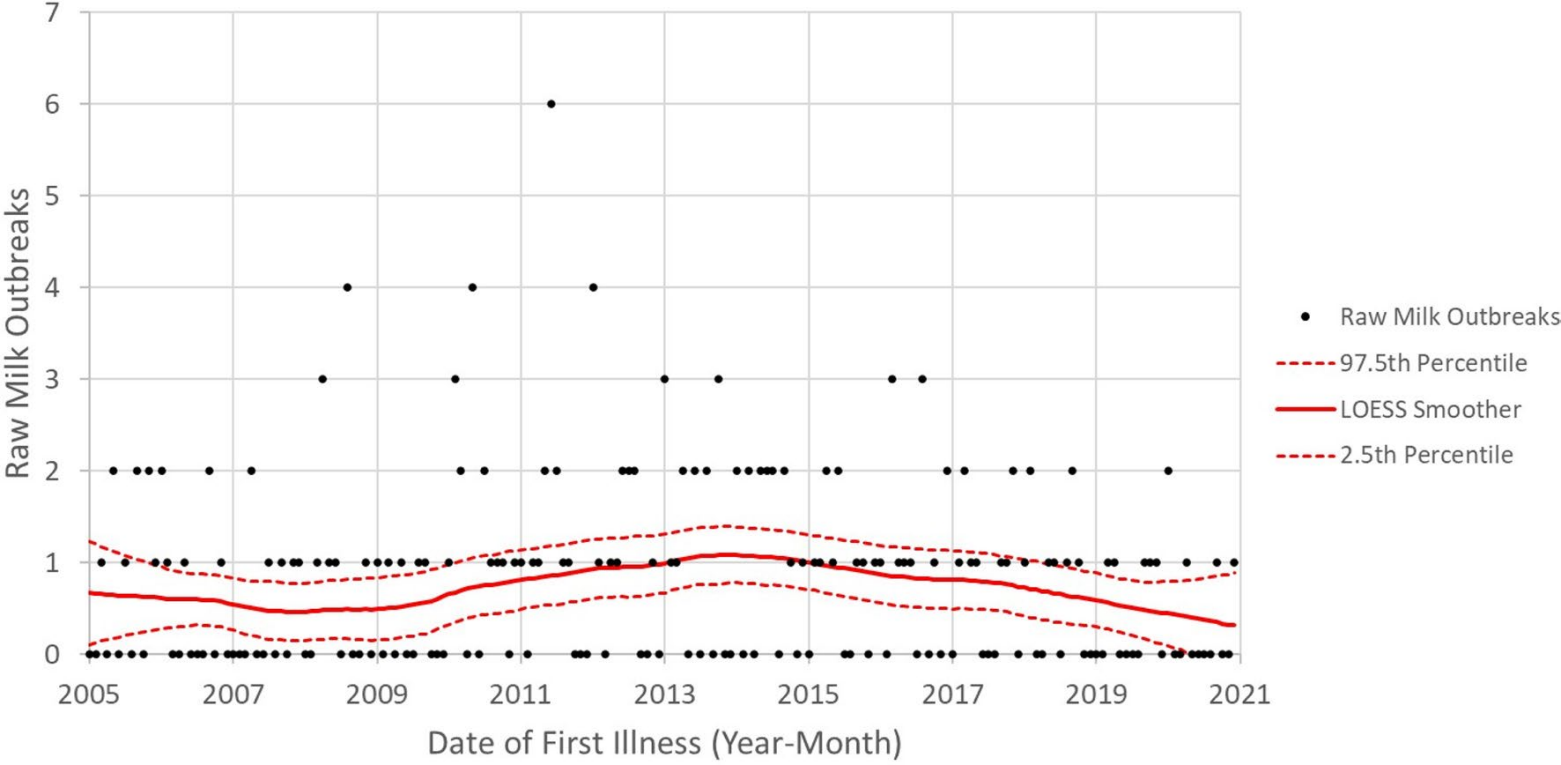
Numbers outbreaks by date of first illness for raw milk (2005–2020) [27] depicted using LOESS Smother (red lines) and 95% confidence intervals (red dashed lines)

Figures 11 and 12 show time series analyses for illnesses and outbreaks, respectively, associated with raw milk, depicted with Locally Weighted Scatterplot Smoothing (LOESS) smoothers and 95% confidence intervals. A LOESS smoother was chosen because it is less susceptible to the influence of outliers and can, therefore, be used to illustrate the time-series trend for a set of data points like the illness and outbreak data (as opposed to something like linear regression). If illnesses and outbreaks were increasing over time, the LOESS smoother would have had a positive slope. Conversely, if illnesses and outbreaks had decreased over time, the LOESS would have had a negative slope. A horizontal LOESS smoother indicates no time-series trend (neither increasing nor decreasing, i.e., flat). The illness trend was flat over the period (Fig. 11), and trends for outbreaks were flat or possibly declining since approximately 2014 (Fig. 12). These trends support the conclusion that raw milk-attributed illnesses and outbreaks did not increase over the period.

#### Graphical Analysis of Trends for Raw Milk by State

**Fig. 13:**
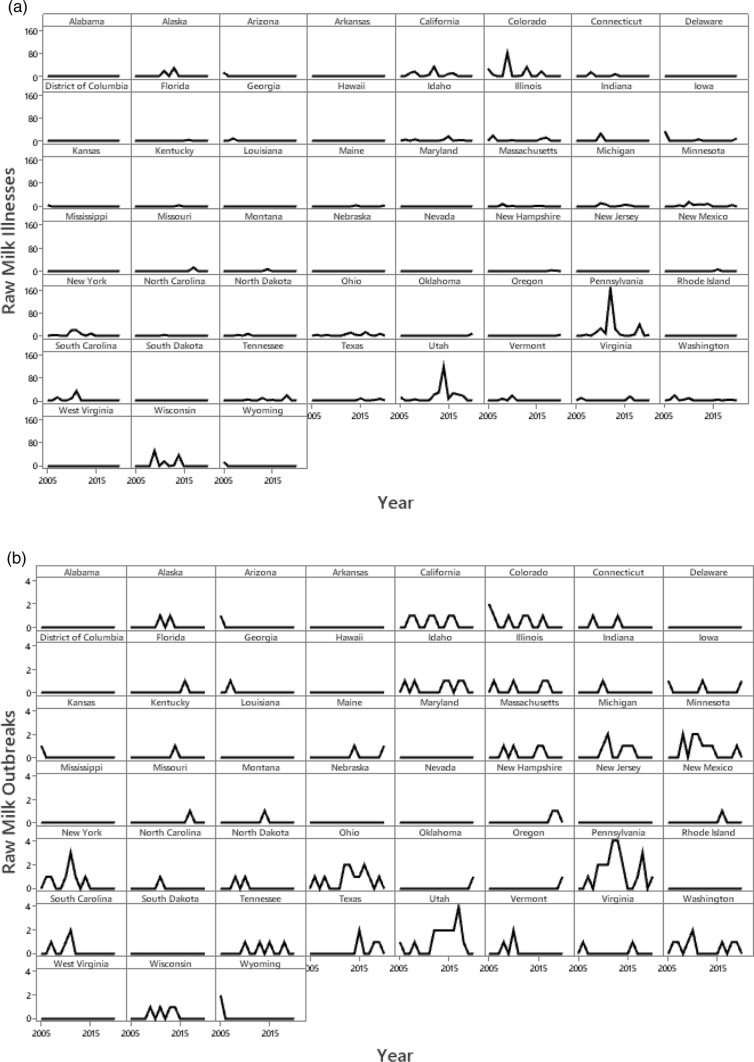
The numbers of raw milk illnesses (**a**) and outbreaks (**b**) by U.S. state reported from 2005 to 2020 [27]. No state demonstrated an increasing trend for this 16-year period

Trends for numbers of illnesses and outbreaks, respectively, associated with raw milk by state-year are presented in Fig. 13a, b to reflect differences in access and regulatory monitoring at the state level. Rates for numbers of illnesses and outbreaks per million person-years adjusted for U.S. Census data by state are presented in Supplementary Table S1.

Additional raw milk data available for New York (NY) and California (CA) are presented in Supplementary Table S2.

**Fig. 14:**
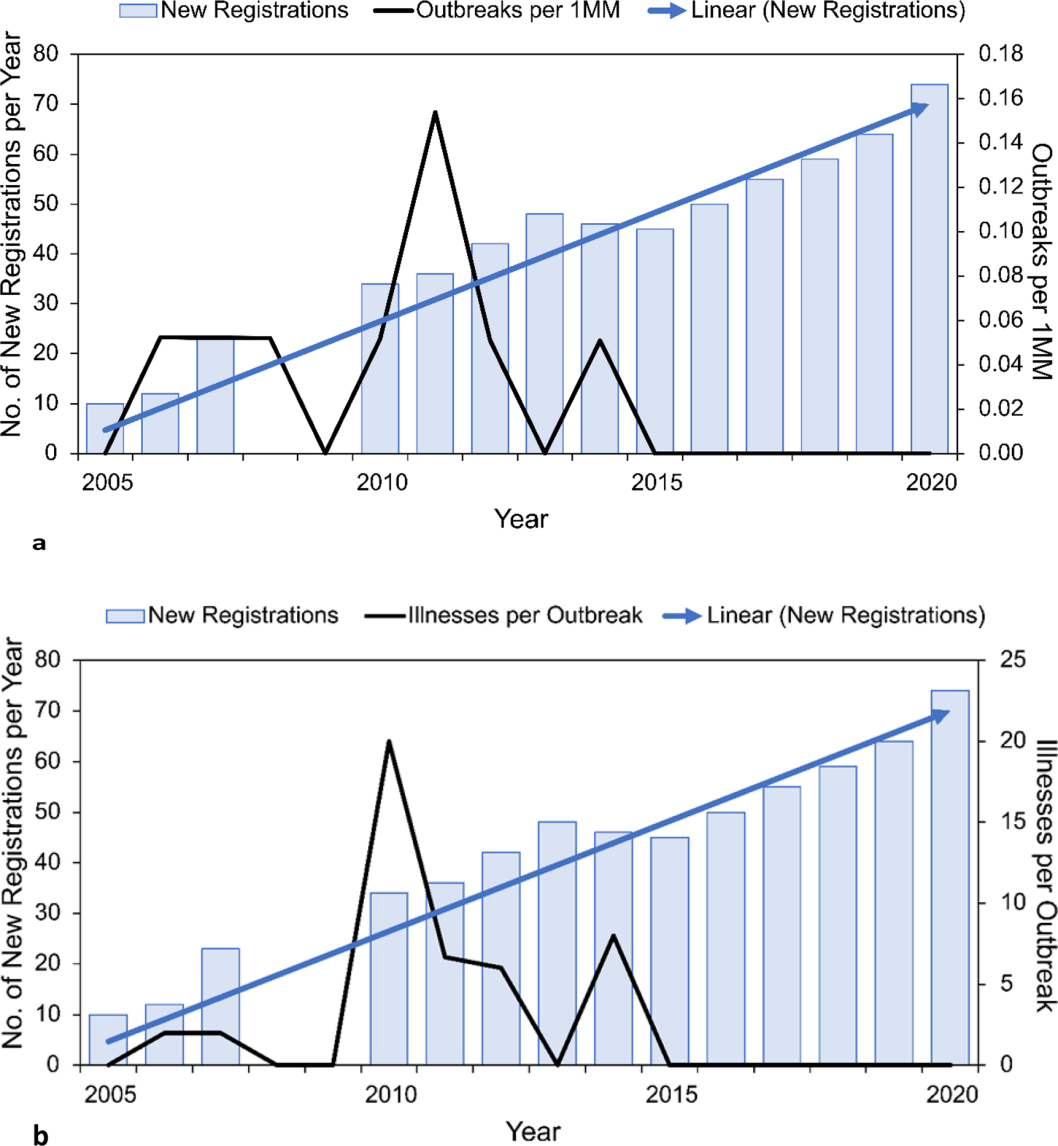
**a** Numbers of annual licenses (2005–2022) approved by NY State for sale of raw milk versus outbreak rates per 1MM people [27, 55]. **b** Numbers of annual licenses (2005–2022) approved by NY State for sale of raw milk versus illnesses per outbreak [27, 55]

New York state permits on-farm sale of raw milk from licensed dairies. The United States Department of Agriculture National Agricultural Statistics Service (USDA NASS) reported that 14,882 million pounds of pasteurized milk (1,730 million gallons) were sold in the state in 2018 (see Supplementary Table 2), but no data on raw milk sales or production were collected. However, data on the number of licenses approved by NY State were obtained by a Freedom of Information Act request in 2022. Nine outbreaks were reported in NY state between 2005 and 2020, with three outbreaks reported in 2011 and one outbreak reported in 2006, 2007, 2008, 2010, 2012, and 2014. Zero outbreaks were reported in NY state for all other years in this period. Data on the numbers of licenses were plotted against outbreak rates adjusted for population (Fig. 14a, b). The Spearman’s rho rank correlation coefficient between outbreak rates and registrations was − 0.647 (p-value = 0.012), indicating that outbreak rates were inversely related to the number of registrations, exactly the opposite of what would be expected if access to raw milk was linked to increasing rates for outbreaks.

**Fig. 15:**
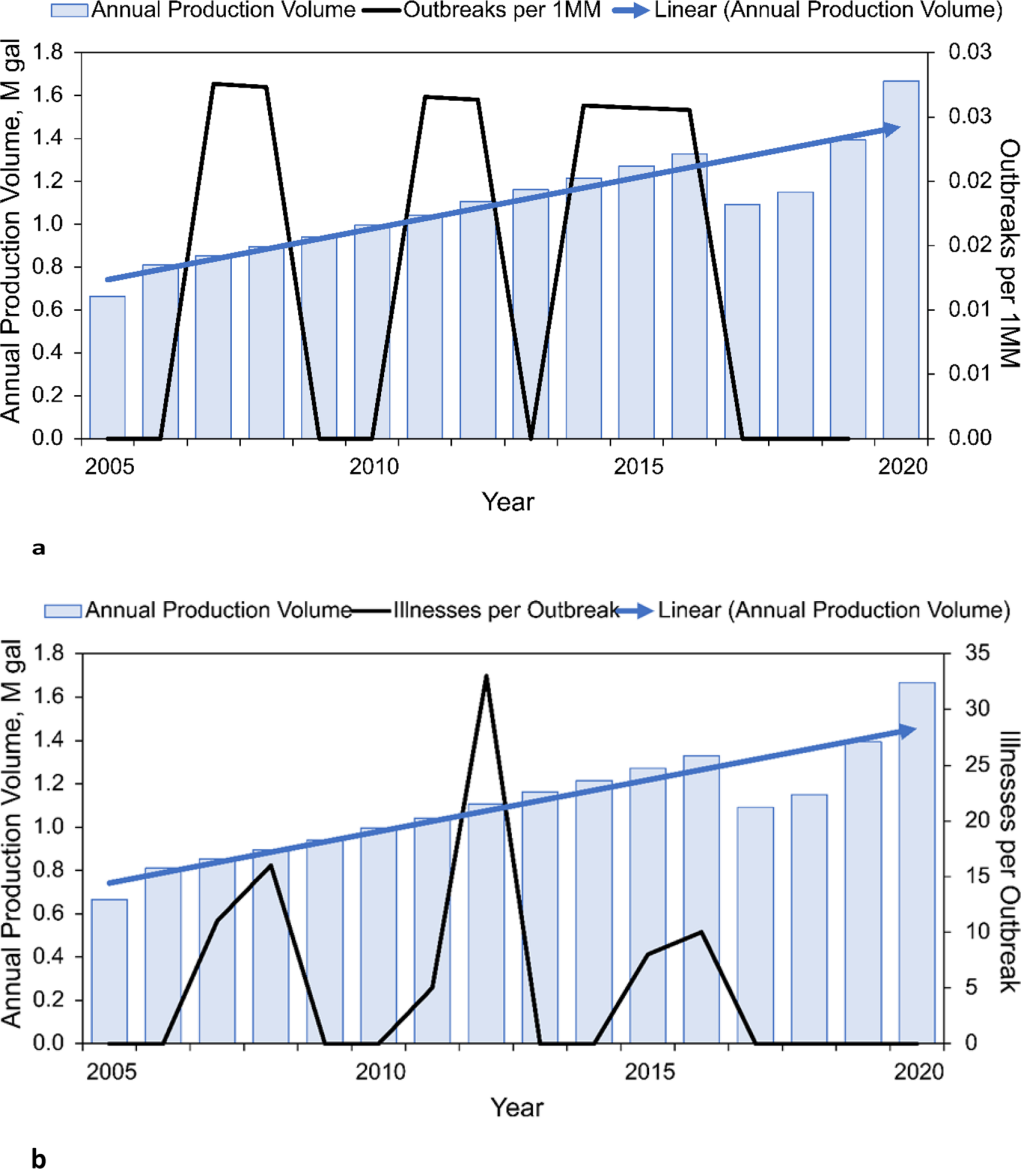
**a** Annual retail production volumes in millions of gallons for one California dairy (personal communication) and outbreak rates per million across the state (not necessarily from this dairy) [27, 56]. **b** Annual retail production volumes in millions of gallons for one California dairy (personal communication) and illnesses per outbreak across the state (not necessarily from this dairy) [27, 56]

The state of CA permits retail sale of raw milk. The USDA NASS reported 40,413 million pounds of pasteurized milk (4699 million gallons) were sold in the state in 2018 (see Supplementary Table 2), but no data on raw milk sales are collected. Data were provided by the largest CA raw dairy on annual retail sales from 2005 to 2020 (personal communication), including 2018 sales of 10 million pounds (1.1 million gallons). Six outbreaks were reported in CA state between 2005 and 2020, with two outbreaks reported in 2015 and one outbreak reported in 2006, 2007, 2008, 2011, 2012, 2014, and 2016. Zero outbreaks were reported in CA for all other years in this period. A total of 83 illnesses and no deaths were reported, 68 campylobacteriosis and 15 illnesses associated with STEC. Figure 15a, b depict annual raw milk production from that CA raw milk dairy in gallons plotted against raw milk outbreak rates in the state. The Spearman’s rho rank correlation coefficient between outbreak rates and production was − 0.117 (p-value = 0.667), indicating that outbreak rates were inversely related to production, exactly the opposite of what would be expected if access to raw milk was linked to increasing numbers of outbreaks. However, the trend was not significant; therefore, the trend was horizontal (neither increasing nor decreasing) despite greater and greater production.

#### Rank-Sum Test Results

Table 3 provides the results for the rank-sum tests for each of the eight individual states and the pooled analysis. The null hypothesis was that the median outbreak rates for both groups were identical. The *p*-values were all greater than common decision thresholds of 5% (0.05) and 10% (0.10) significance; therefore, the null hypothesis could not be rejected. These results suggest that there was no significant increase in the outbreak rates after a change in legal status, i.e., a change in legal classification from “illegal” to “legal” regardless of using Definition 1 (illegal classification = “I”) or Definition 2 (illegal classification = “I” or “P”). Had the *p*-values been less than 0.05 or smaller (e.g., less than 0.01), the null hypothesis would have been rejected, and this would have led to a different inference and suggested that legal definition was a significant predictor of outbreak rates. However, these results are based on sparse data. For example, (1) there was only one outbreak in Kentucky and one outbreak in Montana; (2) there were zero outbreaks in North Dakota after the legal status change from “I” to “H” in 2014; and (3) Wyoming only had only two outbreaks in 2005. In addition, the tests could not be run for Maryland or West Virginia, which had zero outbreaks during the reporting period from 2005 to 2020. While the nonparametric rank-sum test generally provides greater statistical power than the parametric equivalent (e.g., t-test or ANOVA), inferences about statistical significance based on such sparse count data should be made with caution. Nevertheless, the conclusions from these tests are that outbreak rates were no greater after changing raw milk’s legal status from illegal to legal.

**Table 3:** Results for the rank-sum tests for each of the eight individual states and pooled analysis using Definitions 1 or 2 as the grouping variable

Group	*p*-value		Notes
	Legal Definition 1	Legal Definition 2	
Pooled	0.359	0.295	
Kentucky	0.394	N/A	N/A because of only classifications I and H, so Def1 = Def 2
Maryland	N/A	N/A	N/A because of zero outbreaks
Michigan	0.417	N/A	N/A because of only classifications I and H, so Def1 = Def 2
Montana	0.417	N/A	N/A because of only classifications I and H, so Def1 = Def 2
North Dakota	0.215	N/A	N/A because of only classifications I and H, so Def1 = Def 2
Tennessee	0.159	N/A	N/A because of only classifications I and H, so Def1 = Def 2
West Virginia	N/A	N/A	N/A because of zero outbreaks
Wyoming	0.337	N/A	N/A because of only classifications I, H, and F, so Def1 = Def 2

## Discussion

The U.S. CDC NORS data analyzed herein [27] were generated from voluntary passive surveillance systems at local, state, and territorial levels that may not be generalizable to all outbreaks or illnesses associated with reported etiologies [48]. Wikswo and colleagues [48] note that variability in reporting by site and transmission mode may introduce biases in NORS data.

Epidemiologic data from NORS and other passive surveillance systems are subject to limitations. Data quality in NORS is not documented for reported records, particularly reporting capacity and testing rigor from local, state, and territorial facilities [2]. Though our analysis did not focus on outbreaks with multiple etiologies, such outbreaks may reflect improper handling or environmental cross-contamination events at various points in the food chain [2], factors relevant to risk analysis. Inconsistencies in NORS are noted for outbreak and case definitions [2, 48] and in various text strings describing suspect foods and contaminated ingredients.

NORS data are incomplete for many variables [2, 48], including: transmission source, etiology, attack rate (numbers ill/numbers exposed), age range of cases, signs and symptoms, point of contamination in the food chain from production to consumption, contributing factors to illness and its severity, and location (where produced, processed, prepared, and consumed). In addition, differences in reporting rates by state and transmission mode were noted for NORS, limitations that may reflect variable resource availability and prioritizations for outbreak investigations [48]. Other confounding factors may include immune status of cases and underlying health conditions particularly for outbreaks in long-term care facilities, predisposing factors (diet, malnutrition, pharmaceuticals, polluted environments, poverty, cultural stressors), doses of hazards ingested, inhaled, or contacted, and laboratory resource or competency limitations. While NORS includes variables for many potential confounding factors, the key factors from a risk analysis perspective include incompleteness regarding data quality, attack rates, and information on doses or levels of pathogens ingested by consumers, stratified by health outcome. Rarely are these data available in NORS and other passive surveillance systems.

Consistent with a previous analysis of CDC NORS data for the years 2009–2019 [48], Norovirus caused the highest numbers of outbreaks, illnesses, and deaths for the reporting years 2005–2020 analyzed herein. However, foodborne, not person-to-person transmission was associated with the highest numbers of hospitalizations for the current study. Similarly, both studies reported predominance of person-to-person transmission for Norovirus outbreaks, followed by foodborne, with small proportions of illnesses associated with transmission via the environment and water. Our work herein emphasized the major food-pathogen pairs contributing to the burden of foodborne bacterial illness for CDC NORS reporting years 2005–2020.

This section focuses on four aspects of trends in infectious disease that intersect with the need to incorporate systems analysis and quality of risk and risk–benefit analysis, consistent with One Health approaches, to more transparently and effectively balance risks to human health and the planet.

### Burden of Illness Across Transmission Sources

To put the trends for the burdens of infectious diseases in the U.S. discussed herein in broader perspective, the top ten causes of mortality in the U.S. listed for 2019 by the WHO [57] included heart disease, pulmonary disease, stroke, and diabetes, all NCDs potentially linked to unhealthy diet [58], but not to infectious disease.

Expanding One Health research could move the world towards achieving more affordable healthy local diets that contribute to enhanced human health, reduced burdens of both infectious and non-communicable diseases, and lower rates of decline to land, water, and environmental resources. In the U.S., the National Academies’ recent report entitled Operationalizing Sustainable Development to Benefit People and the Planet [59] identified a similarly urgent need for holistic reforms to food systems crucial for addressing food insecurities, food waste, and ecological damages to land, water, and biodiversity, as well as the complex interrelationships of science, economics, and cultural or social science studies in policy-making, in the U.S. and globally.

Aspects of the burden of illness that particularly merit additional deliberation are the need to balance non-communicable and infectious disease burdens, particularly those related to dietary choices by consumers around the world. While pasteurized milk often appears to be assumed ‘zero risk’ in both popular media and the peer-reviewed literature, recent epidemiologic data (Fig. 10) documented a significant illness burden, and recent cohort studies documented increased risk of allergy, asthma, respiratory, and other diseases for pasteurized milk and no increase in diarrheal disease associated with raw milk, as summarized in Dietert and colleagues [21]. The effects of the natural milk microbiota on maintaining balanced gut, immune, neural, and respiratory systems should not be discounted or dismissed without deeper deliberation.

The CDC NORS data on infectious disease [27] included 14,021 foodborne illnesses associated with bacterial, parasitic, and viral pathogens for the recent 16-year period, including 12,781 hospitalizations and 295 deaths. Consistent with the work of Abe and colleagues [25], number of illnesses was considered more relevant than number of outbreaks for representing the burden of illness for risk management decisions. Disease severity and mortality did not appear as reliable metrics for diarrheal illnesses (likely from foodborne, waterborne, and perhaps person-to person transmission) since mortality associated with diarrheal illness in the U.S. for 2019 accounted for only 0.4% of the total U.S. disease burden for mortality [1]. Nevertheless, Supplementary Table S1 provides the full data sets for milkborne outbreaks, including hospitalizations and deaths. As previously mentioned, the persistent dominance of NCDs in burdens of illness for the U.S. and the world in recent years merits deeper and more transparent deliberation so that consumers and regulators are making well-informed decisions about the benefits and risks of foods.

Based on a graphical analysis and simple trend statistics for data on the number of illnesses (Fig. 4), no significant trends were identified for the burden of infectious foodborne illness. Trends for person-to-person transmission were strongly increasing initially, followed by more recent leveling off. This pattern is consistent with that described by Wikswo and colleagues [48], who noted that the initial increase in reporting may have been due to a learning curve for this transmission source rather than an actual increasing rate of illness. Strongly increasing trends were observed for indeterminate and waterborne transmission.

### Consideration of Hazard Identification and Data Quality for Foodborne Illness

The results reported in a previous study of CDC NORS data [48] are generally consistent with results reported herein (Figs. 6 and 7). However, Wikswo and colleagues [48] focused on enteric illness and thus excluded listeriosis. The primary bacterial burden of enteric disease [48] was associated with salmonellosis (51,383 illnesses, 8,038 hospitalizations, and 81 deaths), with 1,512 of 2,449 outbreaks foodborne. Pathogenic *E. coli*, although associated with lower numbers of illnesses, hospitalizations, and deaths than *Salmonella* in the Wikswo study, was associated with the highest case fatality ratio (0.47). In our consideration of Hazard Identification for foodborne pathogens, listeriosis, not salmonellosis or pathogenic *E. coli,* was the primary contributor to mortality. Listeriosis is associated with severe and fatal illness [47, 60], largely from foods, food contact surfaces in processing facilities, and environmental sources. The lowest case-fatality ratio (0.02) in the Wikswo study [48] was reported for campylobacteriosis, consistent with the analysis herein.

Regarding the process of Hazard Identification, the EFSA [61] considered three criteria: (1) high burden of illness in humans; (2) high disease severity in confirmed cases in multiple years; and (3) microbiologic and epidemiologic evidence as important risk factors. For the four major foodborne bacterial hazards considered herein for the CDC NORS dataset, greater than 90% of the burden of illness for the period 2005–2020 was attributed as follows. Due to the rarity of foodborne deaths in this dataset, data on mortality by food-pathogen pair reflects outbreaks with more than 2 deaths in this period.

- The majority of total campylobacteriosis illnesses (4,598) were attributable to pasteurized milk (41%), raw milk (34%), poultry (13%) and mollusks (9%). No campylobacteriosis outbreaks were associated with more than 2 deaths.
  - The majority of the total pathogenic *E. coli* illnesses (4,126) were attributable to leafy-vine-stalk vegetables (53%) and beef (36%), with 6.5% of total illnesses associated raw milk (267 cases). A total of 10 deaths were associated with the leafy-vine-vegetable group, 5 each with outbreaks in spinach in 2006 and in romaine lettuce in 2018.
  - The majority of the total salmonellosis illnesses (22,943) were attributable to poultry (27%), leafy-vine-stalk vegetables (26%), pork (12%), beef (8%), melons (7%), root vegetables (7%), and processed nuts (7%), with < 1% of total illnesses associated raw milk (162 cases). A total of 21 deaths were associated with salmonellosis, 9 with peanut butter, 6 with cucumber, 3 with cantaloupe, and 3 with pot pie, reflecting one outbreak-year per food.
  - The majority of total listeriosis illnesses (532) were associated with melons (29%) and cheeses that were not produced from raw milk (27%), with < 1% of total illnesses (2 cases) purportedly associated with raw milk. We note that although Nichols and colleagues [54] reported similar *L. monocytogenes* stains in raw chocolate milk sampled in 2015 and two human clinical samples from 2014, consumption of raw milk was not confirmed nor were spatial and temporal links of the cases to raw milk established. A total of 78 deaths were associated with listeriosis, 33 with cantaloupe in 2012, 7 with caramel apples in 2014, 3 with prepackaged leafy greens in both 2014 and 2017, 5 with ricotta salata cheese in 2012, 4 with Enoki mushrooms in 2016, and 3 each with pasteurized milk in 2007, ice cream prepared from pasteurized milk in 2010, tuna salad in 2008, and hummus in 2013. No food was identified for an addition 4 listeriosis deaths in both 2011 and 2013.

In addition to CDC NORS data, two recent high-quality studies on listeriosis are relevant to both Hazard Identification work discussed herein and the need to update the existing QMRA from 2003 that estimated relative risks for 13 raw and pasteurized dairy foods [36]: a systematic review [47]; and a report of longterm contamination of ice cream [60]. Sebastianski and colleagues [47] reported *L. monocytogenes* was more likely to be the causative agent in pasteurized dairy outbreaks (p < 0.001) and the proportions of hospitalizations and deaths were higher in pasteurized than in unpasteurized outbreaks (p < 0.01). Conrad and colleagues [60] reported that sanitation deficiencies at ice cream production facilities contributed to 10 listeriosis illnesses in hospitalized patients, of whom 3 developed fatal infections. An earlier study by Pouillot and colleagues [24], documented widespread tolerance or resistance to infection in the general population after ingestion of contaminated ice cream at high pathogen numbers (10^9–10^ cells), though 3 of 10 hospitalized and highly susceptible patients developed fatal infections after ingesting lower estimated pathogen doses (10^6–7^ cells). Together, this evidence suggests that despite estimation of very low risk for ice cream in 2003 [36], different dose–response relationships are essential to predicting the likelihood and severity of cases for resistant and highly susceptible consumers, consistent with mechanistic data on immunological thresholds of resistance [62]. In the twenty years since completion of the 2003 FDA/FSIS listeriosis QMRA [36], little progress has been made in filling knowledge gaps for the overly simplistic dose–response models used [63]. Recent mechanistic studies [62, 64, 65] point to the urgent need for generating biologically and ecologically relevant relationships for doses of pathogens and likelihood and severity of illness in QMRAs. Further, Waller et al. [29] noted shortfalls in risk analysis quality for this QMRA [36].

The EFSA [9, 66] emphasized the importance of evaluating the quality of outbreak evidence for use in trend analysis. Examples of strong evidence might include statistically significant associations of foods with cases, definitive analytical evidence such as attack rate (numbers of cases/numbers exposed), identification of the same strain of causative agent in human cases and a food, food component, and/or environment, quantification of levels of pathogen in food and amounts consumed by cases and others exposed but asymptomatic, and spatial and temporal data identifying points for exposure along the food production and distribution chains.

For 2021, EFSA reported that of 249 campylobacteriosis outbreaks, only 20 were classified as strong-evidence outbreaks. The foods associated with those outbreaks were broiler meats, bovine meats, mixed meats, and mixed foods. The numbers of illnesses, hospitalizations, and deaths associated with campylobacteriosis outbreaks were 1,051, 134, and 6, respectively, in 2021. However, campylobacteriosis did not rank in the top ten food-pathogen pairs based on strong-evidence outbreaks for 2021 (Table 65) [9]. One reason that campylobacteriosis in raw milk was not ranked highly for burden of illness may relate to the largely unacknowledged evidence that healthy consumers appear protected from illness by exposures to low densities of pathogens in raw foods including raw milk prepared for direct human consumption, essential primers for developing and maintaining proper balance between innate and adaptive immune systems [21, 67].

Unfortunately, the U.S. CDC Microsoft Access® database includes sparse or no information about the quality of this evidence, as noted previously by Zhang and colleagues [2]. In the case of milkborne illness, the Interagency Food Safety Analytics Collaboration (IFSAC) annual reports [68, 69] note conflicting data for campylobacteriosis attributed to dairy foods and chicken. For 2017, NORS data attributed over 60% of campylobacteriosis to dairy and less than 20% was attributed to chicken. Other more reliable sources (38 case–control studies; 4 structured expert judgement studies) were cited, with attribution of 1.5% of campylobacteriosis cases to raw milk, compared to 24% to chicken prepared in a restaurant for FoodNet active surveillance sites.

Dairy foods, particularly fluid milk, appear over-represented as a source of campylobacteriosis in the NORS database, perhaps reflecting incomplete investigations (e.g., failure to confirm the presence of clinical strains in suspect food samples; failure to document attack rates and doses of clinical strains consumed that caused and did not cause illness; failure to rule out other foods and other transmission sources) and biased inquiry (e.g., survey questions; consideration of one or multiple suspect transmission sources and foods). EFSA [61] concluded that the epidemiologic evidence for raw milk was insufficient for risk evaluation. Thus, including another table in the Microsoft Access^®^ database documenting the quality of the outbreak evidence would greatly enhance the value of CDC NORS data to future risk analysis applications.

Both One Health approaches and benefit-risk analysis for food systems merit consideration for future work to balance food safety, food security, food quality including sensory characteristics, nutritional content, and, importantly, sustainability in the U.S. and around the world. One of the most influential factors for extending food quality (lengthening shelf-life) and food safety (preventing pathogen growth, survival, infectivity, or virulence) is maintaining proper temperatures for perishable foods throughout supply chains. Yet, refrigeration alone is unlikely to provide reliable control for foodborne pathogens, particularly in the developing world. Rather, we point to an extensive body of literature that demonstrates the synergistic effects of multiple intrinsic and extrinsic factors or hurdles (chemical, physical, and microbial) that, in combination, can function synergistically, exceeding the sum of the effects of individual hurdles to reduce or prevent growth and survival of potential pathogens in foods along the supply chain [31, 70–75]. Ideally, future food systems might incorporate combinations of hurdles acting via different mechanisms or targets designed to improve both the safety and quality of perishable foods while retaining desirable sensory attributes of the raw foods [73]. Further, largely unacknowledged protective mechanisms merit further scrutiny: immunity against development of illness for regular consumers of raw milk [67] and the contribution of the natural milk microbiota to proper balance between innate and adaptive immune systems [21, 76].

An early WHO study in this area [77] related the expanding burden of human NCDs largely to environmental stressors that are typically excluded from consideration in QMRAs and epidemiologic investigations. Further, One Health approaches incorporating sustainable or regenerative agricultural practices and technologies may be essential to reduce ecological damages to land, water, and biodiversity of animals, plants, and microbes in ecosystems stressed by industrial-scale agricultural practices [59] and promote healthy humans and ecosystems. Key studies suggest a need to deliberate not merely the evidence on infectious disease surveillance for foodborne disease, but to profoundly re-envision more optimal structures and functions for small dairy farms in the U.S. and around the world, including regenerative agricultural practices and silvo-pasturing [78–81]. Recent studies point to enhanced health benefits for dairy cows, human dairy consumers, and the environment by expanding grazing, rather than standard commercial grain-based rations, and thereby increasing nutritional benefits (enriched bioactive components, including fatty acid profiles and antioxidant content), increased animal health and welfare, and broader sustainability [13, 82–84].

### Risk Perceptions, Trends, and Conflicting Studies on Raw and Pasteurized Milks

A somewhat perplexing result for U.S. milkborne outbreaks is the predominant association with a particularly fastidious, even fragile, micro-aerophile under laboratory conditions, *Campylobacter jejuni,* that typically caused self-resolving campylobacteriosis. In this period, *Campylobacter* was associated with 1,873 pasteurized milk illnesses and 1,570 raw milk illnesses (Fig. 7A) [27]. The mechanism by which such a pathogen that cannot grow in milk or culture broth at refrigerated temperatures under aerobic conditions has infected thousands of U.S. consumers in this period, more than half associated with pasteurized milk, is uncertain.

It is possible that failures in pasteurization or post-pasteurization contamination could be root causes of the six campylobacteriosis outbreaks in pasteurized milk (one each in 2005, 2006, 2007, 2010, 2012, and 2013). Of note is that one pasteurized milk outbreak from 2006 dominates the numbers of campylobacteriosis illnesses (1,644 of 1,873), likely due to the nature of the pasteurized dairy industry and the scale of distribution not only to local but to wider groups of regional consumers. Although 4 subsequent campylobacteriosis outbreaks in pasteurized milk were recorded after 2006, none exceeded 200 illnesses. It is unclear from information provided in the CDC NORS database what system failures contributed to the 6 outbreaks and what controls might have limited or prevented future outbreaks.

Another possibility that merits deeper investigation is the role of biofilms in persistence and transmission of *Campylobacter,* and other pathogens, that may represent the root cause of milkborne (and other foodborne) diseases. Multiple studies [85–88] document the resistance of many zoonotic pathogens that adhere and aggregate into biofilms, including *Campylobacter,* to chemical and physical (including thermal and non-thermal) interventions and hygienic practices that are typically effective for killing or reducing levels of planktonic or suspended bacteria. Recent research, including One Health approaches, document promising results for chemical, phytochemical, and microbial interventions that can disrupt *Campylobacter* biofilms and perhaps significantly reduce milkborne and foodborne campylobacteriosis in the future [89–92].

Evidence from CDC NORS [27] that pasteurized milk accounted for more than half of milkborne illnesses from 2005 to 2020 (2099/3795 illnesses; 55%; Table 2) may surprise readers of this manuscript. Of course, the disease burden would ideally be adjusted for differential magnitudes of consumption of pasteurized and raw milks for direct risk–risk comparisons in QMRA studies, if data were available for estimating raw milk consumption. Despite the data gap for raw milk consumption, these epidemiologic data support the assertion that neither pasteurized nor raw milk is risk-free.

Opposing positions on the risks and benefits of raw and pasteurized milk abound in the media, on websites, and in the peer-reviewed literature [21, 42]. Certainly, claims about raw milk may arise from two opposing world views: that raw milk is inherently dangerous and always will be, or that raw milk is perfectly safe and always will be. Claims about raw milk risks also invoke fear and dread and appear likely to propagate confirmation bias, excluding evidence that conflicts with particular world views [93]. Claims that raw milk is inherently dangerous appear founded in ideology and dogmas based on late 19th-century science, not the recent data structured as an ‘evidence-map’ by Dietert and colleagues [21] and the analyses reported herein to support dialogue on balancing benefits and risks.

Past studies of raw milk outbreak data for less recent datasets from NORS (1993–2006 [37]; 2007–2012 [39]; 2004–2014 [40]; 2005–2016 [41]; 1998–2018 [42]) provided evidence on numbers of illnesses, outbreaks, hospitalizations, and deaths attributed to raw milk outbreaks in these periods, but limited statistical characterization of the data and models. None of these studies assessed ‘root cause’ as described by Pang and colleagues [18]. The inferences about the data quality of descriptive epidemiologic studies on raw and pasteurized milk merit additional scrutiny.

Langer and colleagues [37] applied the Poisson model for outbreak rates for NORS data for the years 1993–2006 which may be inappropriate for estimating trends due to overdispersion demonstrated for more recent NORS data. In addition, Langer and colleagues [37] noted, “the number of reported dairy-associated outbreaks increased in 1998 after surveillance for foodborne disease outbreaks was enhanced,” suggesting a potential confounding effect for comparing foodborne disease outbreak data collected before and after electronic formats were initiated in 1998. However, we note that the lack of statistical significance for the incidence density ratio for illnesses associated with raw versus pasteurized milk reported by Langer [37] is consistent with the findings from more recent data analyzed herein and by Whitehead and Lake [41].

For the study of Mungai et al. [39], no statistical analysis for trends was provided. For Costard et al. [40], fluid raw milk was not analyzed, but was inappropriately pooled with data for all processed raw dairy commodities (soft and hard cheeses, ice creams, yogurts). The authors did not address the influence of pooling across dairy commodities as a confounding effect for drawing inferences about fluid milk risk separate from processed dairy. In contrast, results from a previous U.S. government study [36] reported risks for 11 separate dairy products ranging from very low to very high risk, challenging the notion that dairy products can be pooled for estimating risks. Neither epidemiologic study provides rigorous statistical analysis for fluid raw milk data.

For the Whitehead and Lake study [41] for years 2005–2016, we note that although the polynomial trend line included in the authors’ Fig. 1 may not be appropriate, these data are consistent with the more recent dataset analyzed herein and reported by Koski and colleagues [42]. Further, a Pearson’s correlation coefficient was reported (0.26, 95% confidence interval − 0.40 to 0.74) as suggestive evidence that the population-adjusted outbreak rates do not support the assertion from previous studies that increased legal access to raw milk leads to higher outbreak rates. The authors reported a very slight decrease in outbreak rates per million people in states 4 years before (0.279) and after (0.272) legalization of access to raw milk in the 4 states for which data was available (Whitehead and Lake, Table 3), though no statistical analysis for this comparison was reported. These findings are consistent with the findings presented herein (Figs. 11, 12, 13; Table 3) using different statistical methods.

For the Koski study [42], data for 1998–2018 were combined from two surveillance systems, the older CDC Foodborne Disease Outbreak Reporting System (FDORS) and the current National Outbreak Reporting System (NORS). The validity of testing for trends across different surveillance systems that were not conformable is clearly questionable [94]. Koski’s use of the older surveillance system data as a reference for testing statistical significance for the recent system is thus problematic. In addition, the authors also did not adjust outbreak and illness numbers for population changes over this period as conducted in previous studies [37, 39, 41]. Again, conclusions about trends over time are of questionable validity.

Further, CDC NORS does not provide identification of sources of raw milk by the outbreak (e.g., from illegal (black market) access or from one of multiple legal status options for some states; see Supplemental Information, Table S2). Unstated assumptions by Koski and colleagues in effect assign a legal access category to over 100 different outbreaks, though NORS is incomplete on this point. In reality, great uncertainty exists about the validity of retrospectively assigning source legal classification and assuming consistency with state requirements for licensure to historic outbreaks without further documentation. Considering risk analysis principles, the claim of Koski and colleagues that retail legal status is associated with more outbreaks may be misleading, as adjustment for greater consumption in retail states than other states may be necessary for valid statistical comparisons, considering Fig. 13 from the present analysis. Also, the number of illnesses, not the number of outbreaks, is the relevant metric for the burden of illness and risk analysis [25]. Interestingly, Koski and colleagues reported that numbers of raw milk illnesses did not increase significantly over the years 1998–2018, consistent with data in Fig. 11. Thus, the conclusion of the Koski study, that ‘state laws resulting in increased availability of unpasteurized milk are associated with more outbreak-associated illnesses and outbreaks’, is not supported by the available evidence. The conclusion of the Koski study [42] conflicts with the statistical analyses herein (Table 3) that does not support change in state legal status as an important determinate or predictor of outbreak rates for raw milk.

Although Koski and colleagues [42] claimed increasing trends in the number of outbreaks, the authors’ Fig. 2 spans two surveillance systems. Four of six selected models comparing numbers of illnesses and outbreaks by assigned legal status demonstrated a lack of significantly different P-values (authors’ Table 5). Further, other studies that considered such data spanning the two surveillance systems applied adjustments, weighting more recent data higher and historic data lower in estimating trends and confidence intervals [68, 69]. Further, gaps in data quality (e.g., assigned but unverified legal status by outbreak) and lack of weighting or separating the data from the two surveillance systems require more consideration before the reliability of the trends reported by Koski and colleagues can be evaluated.

In our view, the available CDC NORS data do not support the claim that raw milk is an inherently dangerous food. Nor do current microbiology data support this claim, as data from monitoring programs for raw milk produced for direct human consumption are rarely positive for the presence of any of the major foodborne pathogens (≤ 0.01% positive) [21]. Further, suppression of growth of the major bacterial pathogens associated with the burden of illness for raw milk was demonstrated in a recent pilot study at the recommended refrigeration temperature [95].

### Transparency About Basis of Knowledge for Burdens of Infectious and Non-communicable Disease

Dietert [96] cautioned that rigid adherence to seven ‘outdated twentieth-century scientific dogmas’ continues to mischaracterize human health and assessments of risks to human health and safety well into the twenty-first century. Perhaps the most relevant of these outdated dogmas for readers of the work described herein is: ‘most microbes are a threat to human health’ (authors’ Table 1, Dogma 3) [96]. Indeed, the characterization of microbes as ‘germs’ is inconsistent with extensive 21st-century evidence that microbes typically function as partners in healthy ecosystems, including healthy human superorganisms [97].

Consumption of a largely over-processed diet, as well as malnutrition and food insecurity, likely contribute to the global disease burden, particularly regarding NCDs [15, 98]. In contrast, nutrient-dense raw foods including their natural microbiota might strengthen innate colonization resistance against pathobionts typical of healthy human superorganisms, complete with the microbiota, their partners in heath. Recent perspectives suggest the need to expand our paradigms about dietary intakes to consider developing Recommended Daily Allowances for microbes, not just for vitamins and nutrients [99, 100].

Further, raw and pasteurized milks are clearly associated with both risks and benefits. Dietert and colleagues [21] compiled and structured the evidence for raw and pasteurized milk using a formal benefit-risk analysis method that Wiedemann and colleagues [101] described as ‘linking two opposing world views’ regarding another controversial topic at the time (nanotechnology) as ‘information’. Further, evidence maps on nanotechnology studies, designed as structured argumentation, provided a path to increasing reliability and transparency for resolving mixed messages that invoke fear and dread, and likely bias, about poorly characterized benefits and risks.

Unfortunately for society, it seems that rigid adherence to paradigms based on science from the nineteenth and twentieth centuries may be exacerbating the epidemic of NCDs, as well as contributing to the loss of human microbiota. Such paradigms appear to contribute to intentional dismissal or exclusion of confounding factors inherent in observational studies and the documented societal effects of urbanization of dairies by an unscrupulous distillery industry at the turn of the nineteenth century [43, 44, 102–107].

Claims that raw milk is inherently dangerous appear founded in ideology and 19th-century science, not the data and comprehensive analysis of the burden of disease described herein from recent U.S. data. Our findings are consistent with one previous study [41] and inconsistent with some aspects of others [39, 42]. We invite others to explore the available evidence from the U.S. and other countries using high quality data and rigorous statistical methods for improved transparency and wider deliberation of the evidence on a global scale.

Transparency is needed in communications to the public about the quality and safety of raw milk produced for direct human consumption in the twenty-first century using best practices [21, Table 1]. Interestingly, five U.S. states (GA, IO, ND, UT, WY) passed laws that expanded access to raw milk and raw milk products in 2023, despite unsupported and misleading claims that raw milk poses high risk to human health. It seems that a dramatic paradigm shift is beginning that supports broader public discourse and deepening of the common knowledge base among diverse stakeholders, from analysts and regulators to raw milk producers and consumers and their legislators.

Of critical importance from our perspective is the development and validation of transdisciplinary evidence-based models incorporating data for the dense and diverse natural milk microbiota of healthy cows. For example, bacterial densities often exceed 10^4^ counts per mL, and diverse genera are frequently represented in raw milk, including: *Aerococcus, Bacteroides, Brevundimonas, Burkholderia, Clostridiales, Corynebacterium, Cupriavidus, Enhydrobacter, Enterococcus, Faecalibacterium, Fusobacterium, Lactobacillus, Lactococcus, Leuconostoc, Janthinobacterium, Pediococcus, Prevotella, Propionibaterium, Pseudomonas, Rhodocyclaceae, Ruminococcus, Sediminibacterium, Staphylococcus, Stenotrophomonas, Streptococcus, Succiniclasticum, Succinivibrionaceae,* and *Xanthomonadaceae* [21, 45]. Yet foodborne pathogens are detected in less than one in a thousand monitoring samples of raw milk produced for direct human consumption, in contrast to studies of pre-pasteurized milk documented by Dietert and colleagues [21] and Williams and colleagues [108]. The dense and diverse natural microbiota of milk, and other raw foods, likely contribute to declining pathogen survival and competitive exclusion of foodborne pathogens from foods [95] and colonization resistance in consumers, particularly at the low levels documented in naturally contaminated milk samples [109]. The lack or reduction of the dense and diverse natural milk microbiota may account for higher rates of *L. monocytogenes* growth with increasing milk pasteurization temperatures [110].

The trend analysis for fluid milk in the U.S. described herein and the body of evidence for the benefit-risk analysis of milk [21] are intended to increase transparency about the available body of evidence regarding the controversial issues around benefits and risks of raw foods, and more importantly, to promote education and research to fill data gaps for benefit-risk assessment critical to developing evidence-based regulations and policies that balance benefits and risks in the near future. Analyses of U.S. CDC NORS data for cheese and leafy greens are underway. Long-term, we seek potential partners for an international workshop to launch a series of exercises of analytic-deliberative process [28, 111] on balancing food safety and food security in future years.

## Conclusions

The lack of increasing trends in the burden of foodborne disease for U.S. outbreak data versus strongly increasing trends for other transmission sources documented herein provides a comprehensive perspective that can inform quality analysis for benefits and risks of communicable diseases and focus limited global resources on significant challenges for human health and well-being, and the health of the planet.

The available evidence does not support the assumption of zero risk for pasteurized milk nor the assumption of an increasing trend in the burden of illness after a change in state legal status for raw milk.

The need identified by the WHO 75th World Health Assembly [15] to strengthen risk analysis for foods is consistent with the implications of our work. Further, our work aligns with the need for comprehensive, radical transformations of current oversimplified and often dysfunctional food systems into multi-sector sustainable food systems that incorporate complex interdependencies to optimize the six dimensions of food security [8]. However, epidemiologic evidence alone, particularly the descriptive epidemiologic evidence of undocumented quality discussed herein [27], is insufficient to characterize complex food systems and the variabilities and uncertainties inherent in predicting risks to human and environmental health. For future evidence-based risk management, transdisciplinary risk analysis methodologies are essential to balance both communicable and non-communicable diseases, as well as both food safety and food security, considering scientific, sustainable, economic, cultural, social, and political factors to support health and wellness for humans and ecosystems.

## Supplementary Information

Below is the link to the electronic supplementary material.

Supplementary file1 (PDF 2022 KB)

## Data Availability

Data used in the analyses were obtained from publicly available US governmental sources (CDC and Census Bureau). Key portions of the data are provided in supplementary information so that others may repeat the analyses.
