# Supplementary material for: Trends in Burdens of Disease by Transmission Source (USA, 2005–2020) and Hazard Identification for Foods: Focus on Milkborne Disease
Source: J Epidemiol Glob Health. 2024 Mar 28;14(3):787–816. doi: 10.1007/s44197-024-00216-6 (PMC11442898; doi:10.1007/s44197-024-00216-6)
Supplement: Supplementary file 1 — Supplementary file1 (PDF 2022 KB) [file 44197_2024_216_MOESM1_ESM.pdf]

1. SUPPLEMENTAL INFORMATION

1.1. Information on NORS database tables and relationships

The major NORS database tables and relationships used for this analysis are depicted below: NORSMain; 5 tables on food; 3 tables on water; and a table each on etiology, symptoms, and animal contact. The tables are linked by the unique identifier CDCID.

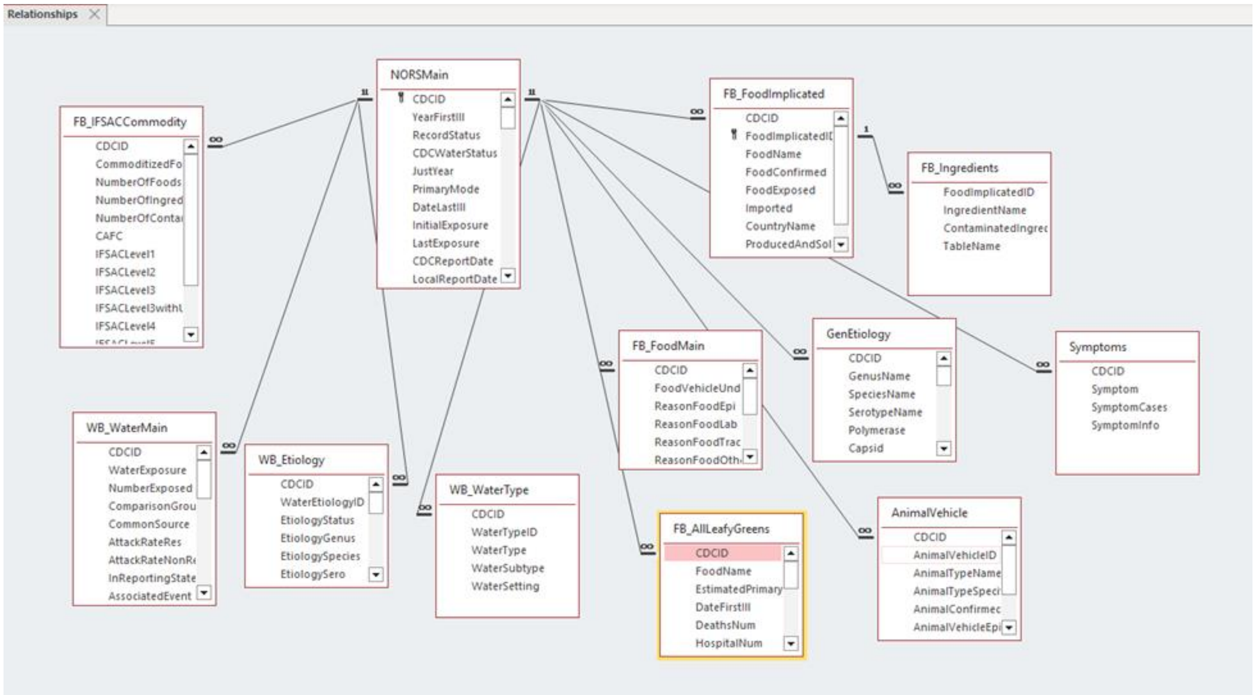

Fig. S1 NORS database tables used in this analysis and their relationships.

1.2 Time-Series Trends in Outbreak, Illness, Hospitalization Rates Associated with Raw Milk

Figures S2, S3, and S4 show bar charts of illness, outbreak, and hospitalization rates, respectively, as a function of time and grouped by state.

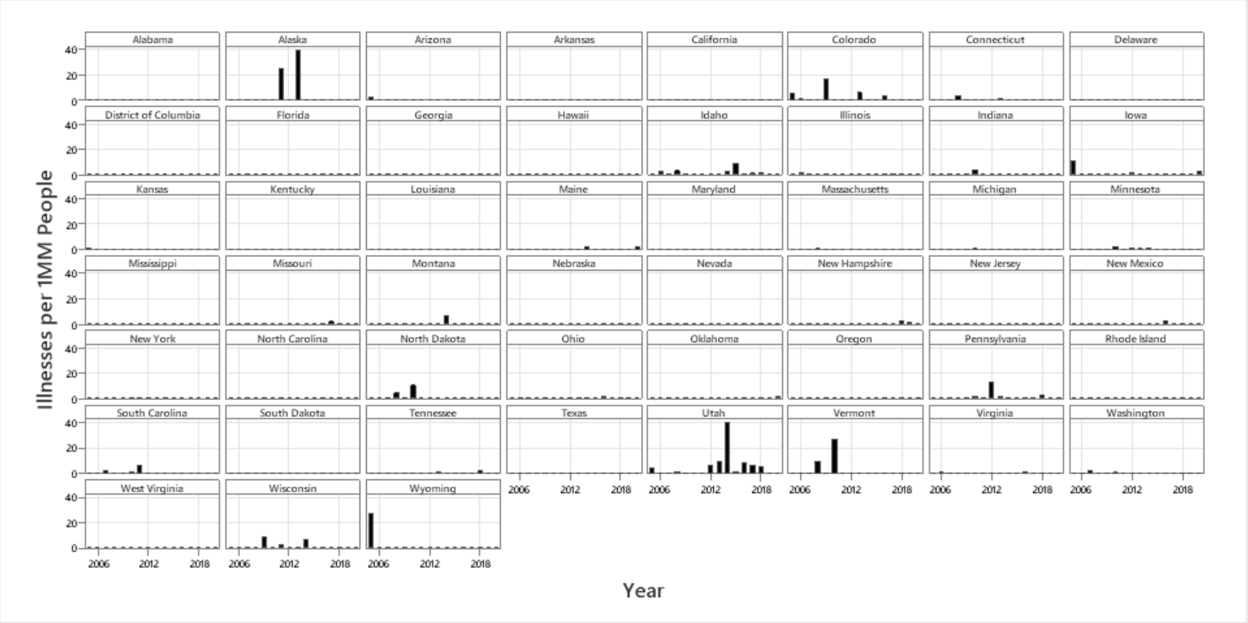

Fig. S2. State-level bar charts of raw milk-related illness incidence rates (illnesses per 1MM people) on the y-axis as a function of time (year) on the x-axis.

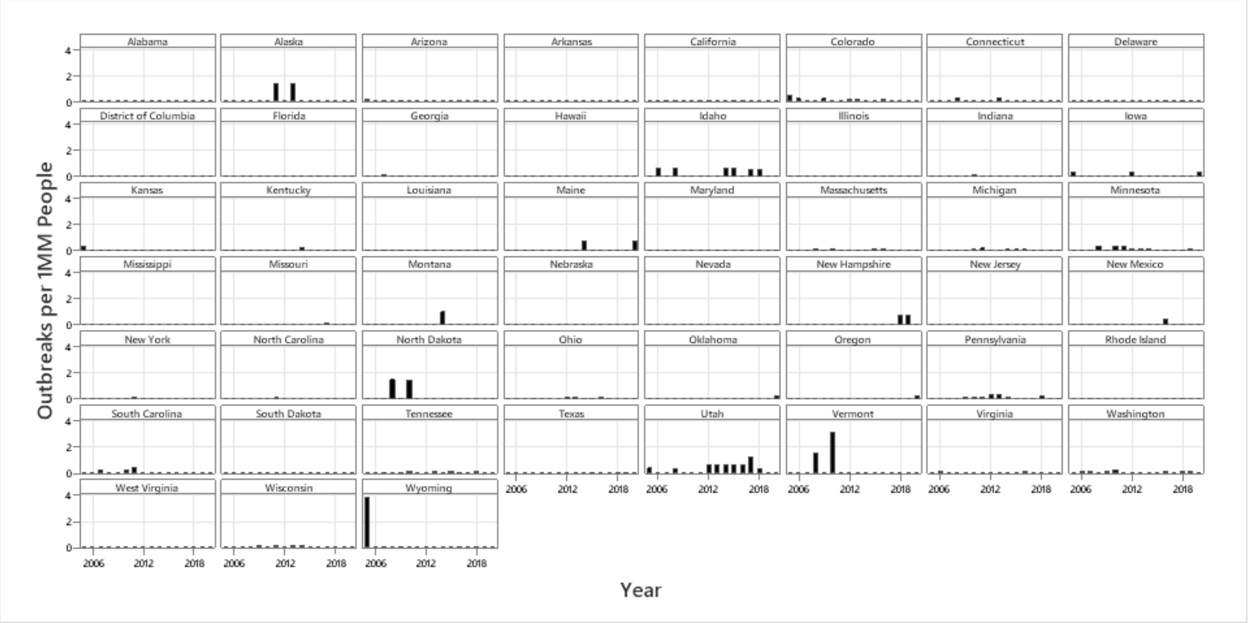

Fig. S3. State-level bar charts of raw milk-related outbreak incidence rates (outbreaks per 1MM people) on the y-axis as a function of time (year) on the x-axis.

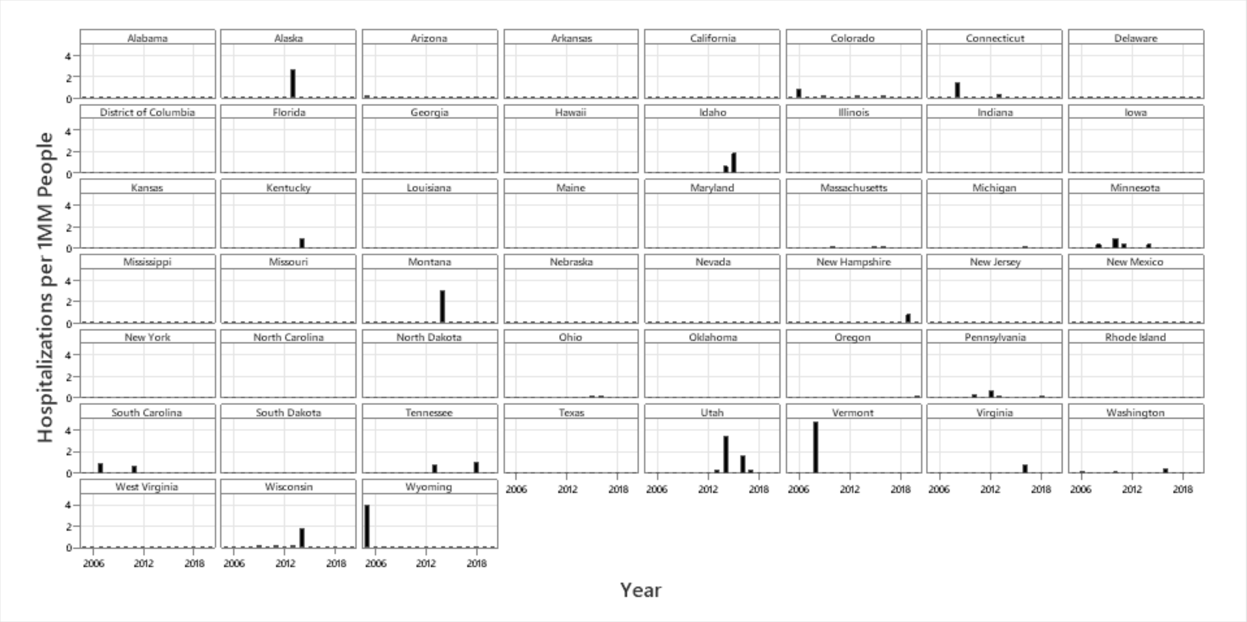

Fig. S4. State-level bar charts of raw milk-related hospitalization incidence rates (hospitalizations per 1MM people) on the y-axis as a function of time (year) on the x-axis.

1.3 Time-Series Trends in Legal Status and Registrations for Raw Milk

Figures S5 and S6 show the time-series trends in legal status. Only nine of the 50 states observed a switch in legal status somewhere between 2005 and 2020 (Kentucky, Maryland, Michigan, Montana, North Dakota, Ohio, Tennessee, West Virginia, and Wyoming) according to Definition 1 (only "I" is illegal). Under Definition 2, Maryland did not switch legal status and North Carolina switched legal status (from "P" to "H" in 2018). Fig. S6 shows the time-series trends in the number of new registrations issued each year for the seven states (California, Colorado, Maine, Massachusetts, New York, Texas, and Utah) that responded to Freedom of Information Act (FOIA) requests.

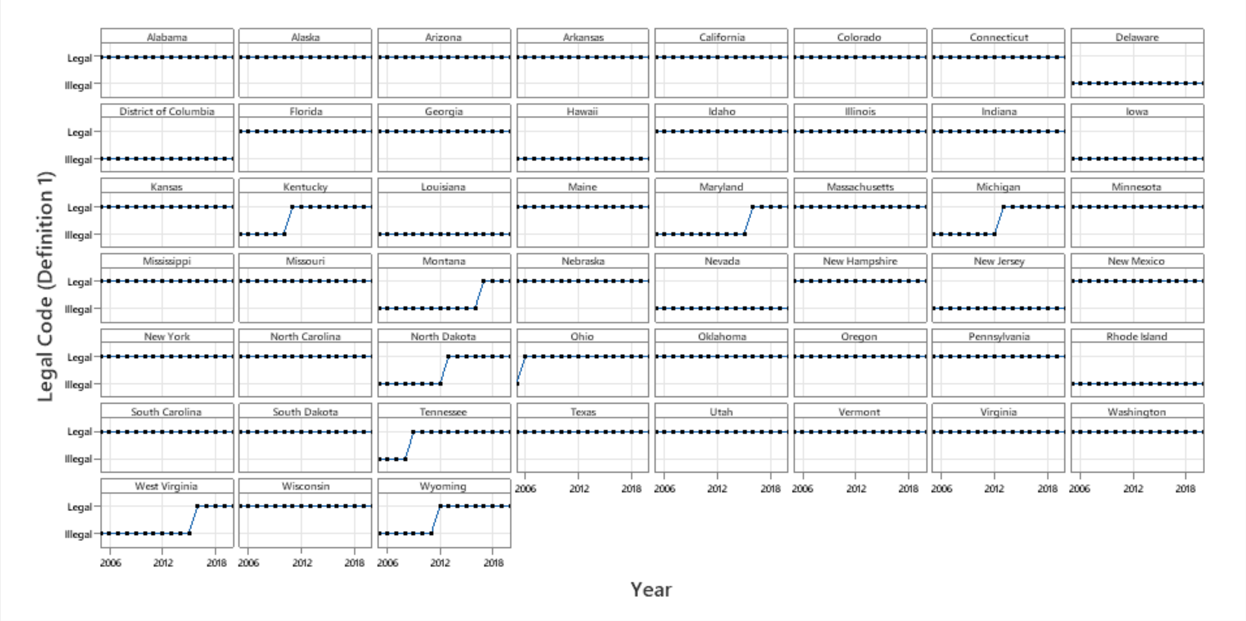

Fig. S5. State-level scatterplots of binary legal status (0=Illegal, 1=Legal) on the y-axis as a function of time (year) on the x-axis. The legal code classification uses Definition 1 (only "I" is illegal).

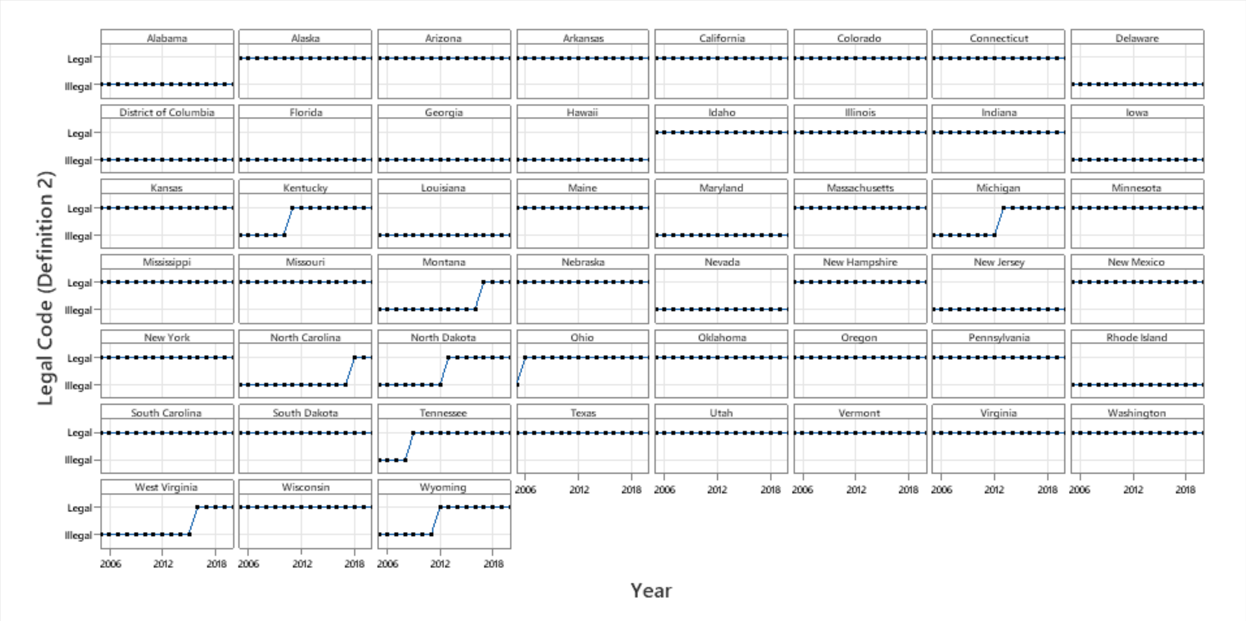

Fig. S6. State-level scatterplots of binary legal status (0=Illegal, 1=Legal) on the y-axis as a function of time (year) on the x-axis. The legal code classification uses Definition 2 ("I" or "P" illegal).

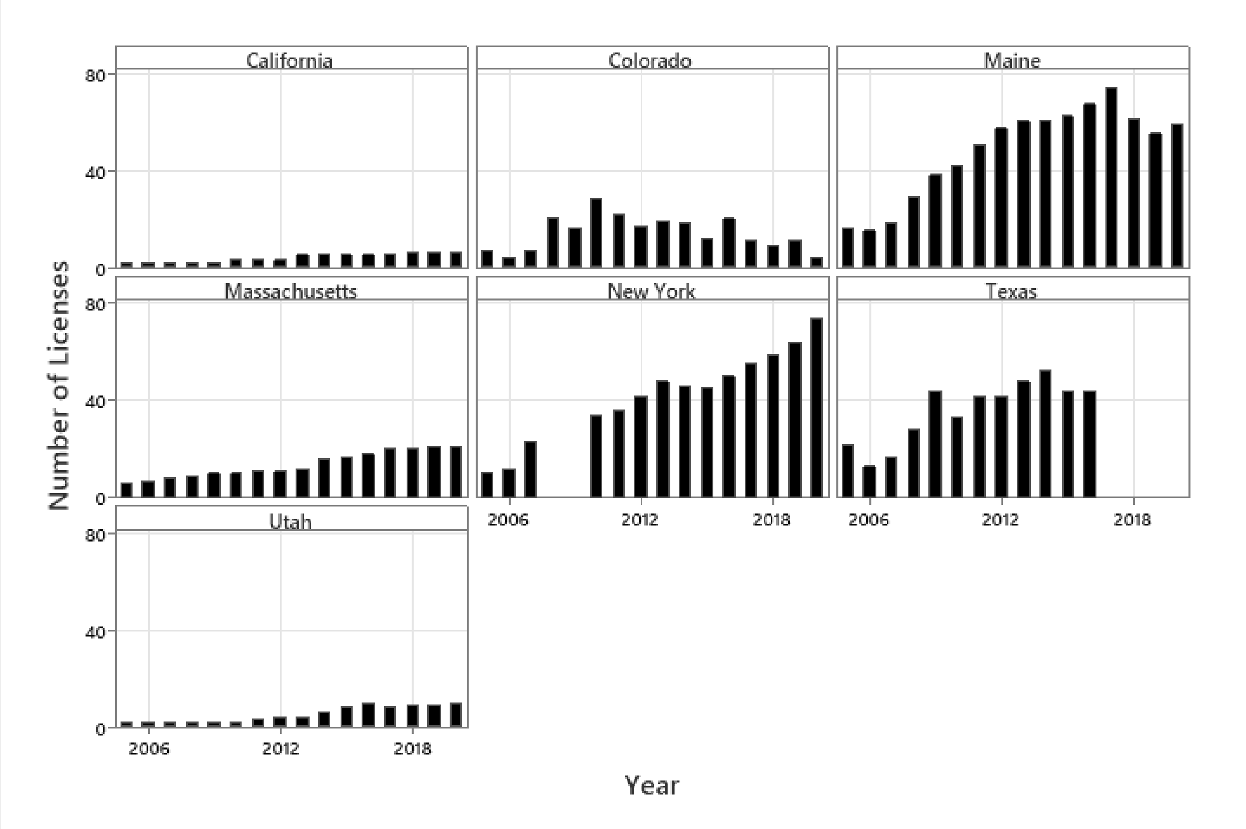

Fig. S7. State-level bar charts of number of licenses/permits issued on the y-axis as a function of time (year) on the x-axis for the seven states (California, Colorado, Maine, Massachusetts, New York, Texas, and Utah) that responded to Freedom of Information Act (FOIA) requests for the number of new raw milk licenses (registrations) issued each year from 2005 to 2020. Data were not available for New York in years 2008 and 2009, or Texas from 2017 to 2022.

1.4. Supporting Tables

Table S1. Data table of raw milk-related outbreaks, illnesses, hospitalizations, and deaths in the United States for the years 2005-2020. The data table combines the raw milk-related outbreaks, illnesses, and hospitalizations, U.S. census populations for each state and year (state-year) (see Section 2.3), state legal classifications by state-year (see Section 2.4), and the reported numbers of licenses/permits issued to dairies approved to sell raw milk (see Section 2.4). The outbreak, illness, and hospitalization counts in each year were expressed as rates per one million persons, i.e., outbreaks/1MM, illnesses/1MM, and hospitalizations/1MM, respectively. Many of the cells under "Number Licenses" are blank (missing data) due to us not having the information for 43 of the 50 states. The data table was then used as input to the statistical analysis.

State	Year	Population	Legal Class	Def 1	Def 2	Number Licenses	Outbreaks	Illnesses	Hospitalizations	Deaths	Outbreak Rate Per 1MM	Illnesses Rate Per 1MM	Hospitalization Rate Per 1MM
Alabama	2005	4,569,805	P	Legal	Illegal		0	0	0	0	0.000	0.000	0.000
Alabama	2006	4,628,981	P	Legal	Illegal		0	0	0	0	0.000	0.000	0.000
Alabama	2007	4,672,840	P	Legal	Illegal		0	0	0	0	0.000	0.000	0.000
Alabama	2008	4,718,206	P	Legal	Illegal		0	0	0	0	0.000	0.000	0.000
Alabama	2009	4,757,938	P	Legal	Illegal		0	0	0	0	0.000	0.000	0.000
Alabama	2010	4,785,514	P	Legal	Illegal		0	0	0	0	0.000	0.000	0.000
Alabama	2011	4,799,642	P	Legal	Illegal		0	0	0	0	0.000	0.000	0.000
Alabama	2012	4,816,632	P	Legal	Illegal		0	0	0	0	0.000	0.000	0.000
Alabama	2013	4,831,586	P	Legal	Illegal		0	0	0	0	0.000	0.000	0.000
Alabama	2014	4,843,737	P	Legal	Illegal		0	0	0	0	0.000	0.000	0.000
Alabama	2015	4,854,803	P	Legal	Illegal		0	0	0	0	0.000	0.000	0.000
Alabama	2016	4,866,824	P	Legal	Illegal		0	0	0	0	0.000	0.000	0.000
Alabama	2017	4,877,989	P	Legal	Illegal		0	0	0	0	0.000	0.000	0.000
Alabama	2018	4,891,628	P	Legal	Illegal		0	0	0	0	0.000	0.000	0.000
Alabama	2019	4,907,965	P	Legal	Illegal		0	0	0	0	0.000	0.000	0.000
Alabama	2020	5,031,362	P	Legal	Illegal		0	0	0	0	0.000	0.000	0.000
Alaska	2005	666,946	H	Legal	Legal		0	0	0	0	0.000	0.000	0.000
Alaska	2006	675,302	H	Legal	Legal		0	0	0	0	0.000	0.000	0.000
Alaska	2007	680,300	H	Legal	Legal		0	0	0	0	0.000	0.000	0.000
Alaska	2008	687,455	H	Legal	Legal		0	0	0	0	0.000	0.000	0.000
Alaska	2009	698,895	H	Legal	Legal		0	0	0	0	0.000	0.000	0.000
Alaska	2010	713,982	H	Legal	Legal		0	0	0	0	0.000	0.000	0.000
Alaska	2011	722,349	H	Legal	Legal		1	18	0	0	1.384	24.919	0.000
Alaska	2012	730,810	H	Legal	Legal		0	0	0	0	0.000	0.000	0.000
Alaska	2013	737,626	H	Legal	Legal		1	29	2	0	1.356	39.315	2.711
Alaska	2014	737,075	H	Legal	Legal		0	0	0	0	0.000	0.000	0.000
Alaska	2015	738,430	H	Legal	Legal		0	0	0	0	0.000	0.000	0.000

State	Year	Population	Legal Class	Def 1	Def 2	Number Licenses	Outbreaks	Illnesses	Hospitalizations	Deaths	Outbreak Rate Per 1MM	Illnesses Rate Per 1MM	Hospitalization Rate Per 1MM
Alaska	2016	742,575	H	Legal	Legal		0	0	0	0	0.000	0.000	0.000
Alaska	2017	740,983	H	Legal	Legal		0	0	0	0	0.000	0.000	0.000
Alaska	2018	736,624	H	Legal	Legal		0	0	0	0	0.000	0.000	0.000
Alaska	2019	733,603	H	Legal	Legal		0	0	0	0	0.000	0.000	0.000
Alaska	2020	732,923	H	Legal	Legal		0	0	0	0	0.000	0.000	0.000
Arizona	2005	5,839,077	R	Legal	Legal		1	13	1	0	0.171	2.226	0.171
Arizona	2006	6,029,141	R	Legal	Legal		0	0	0	0	0.000	0.000	0.000
Arizona	2007	6,167,681	R	Legal	Legal		0	0	0	0	0.000	0.000	0.000
Arizona	2008	6,280,362	R	Legal	Legal		0	0	0	0	0.000	0.000	0.000
Arizona	2009	6,343,154	R	Legal	Legal		0	0	0	0	0.000	0.000	0.000
Arizona	2010	6,407,342	R	Legal	Legal		0	0	0	0	0.000	0.000	0.000
Arizona	2011	6,473,416	R	Legal	Legal		0	0	0	0	0.000	0.000	0.000
Arizona	2012	6,556,344	R	Legal	Legal		0	0	0	0	0.000	0.000	0.000
Arizona	2013	6,634,690	R	Legal	Legal		0	0	0	0	0.000	0.000	0.000
Arizona	2014	6,732,873	R	Legal	Legal		0	0	0	0	0.000	0.000	0.000
Arizona	2015	6,832,810	R	Legal	Legal		0	0	0	0	0.000	0.000	0.000
Arizona	2016	6,944,767	R	Legal	Legal		0	0	0	0	0.000	0.000	0.000
Arizona	2017	7,048,088	R	Legal	Legal		0	0	0	0	0.000	0.000	0.000
Arizona	2018	7,164,228	R	Legal	Legal		0	0	0	0	0.000	0.000	0.000
Arizona	2019	7,291,843	R	Legal	Legal		0	0	0	0	0.000	0.000	0.000
Arizona	2020	7,179,943	R	Legal	Legal		0	0	0	0	0.000	0.000	0.000
Arkansas	2005	2,781,097	F	Legal	Legal		0	0	0	0	0.000	0.000	0.000
Arkansas	2006	2,821,761	F	Legal	Legal		0	0	0	0	0.000	0.000	0.000
Arkansas	2007	2,848,650	F	Legal	Legal		0	0	0	0	0.000	0.000	0.000
Arkansas	2008	2,874,554	F	Legal	Legal		0	0	0	0	0.000	0.000	0.000
Arkansas	2009	2,896,843	F	Legal	Legal		0	0	0	0	0.000	0.000	0.000
Arkansas	2010	2,921,998	F	Legal	Legal		0	0	0	0	0.000	0.000	0.000
Arkansas	2011	2,941,038	F	Legal	Legal		0	0	0	0	0.000	0.000	0.000
Arkansas	2012	2,952,876	F	Legal	Legal		0	0	0	0	0.000	0.000	0.000
Arkansas	2013	2,960,459	F	Legal	Legal		0	0	0	0	0.000	0.000	0.000
Arkansas	2014	2,968,759	F	Legal	Legal		0	0	0	0	0.000	0.000	0.000
Arkansas	2015	2,979,732	F	Legal	Legal		0	0	0	0	0.000	0.000	0.000
Arkansas	2016	2,991,815	F	Legal	Legal		0	0	0	0	0.000	0.000	0.000
Arkansas	2017	3,003,855	F	Legal	Legal		0	0	0	0	0.000	0.000	0.000

State	Year	Population	Legal Class	Def 1	Def 2	Number Licenses	Outbreaks	Illnesses	Hospitalizations	Deaths	Outbreak Rate Per 1MM	Illnesses Rate Per 1MM	Hospitalization Rate Per 1MM
Arkansas	2018	3,012,161	F	Legal	Legal		0	0	0	0	0.000	0.000	0.000
Arkansas	2019	3,020,985	F	Legal	Legal		0	0	0	0	0.000	0.000	0.000
Arkansas	2020	3,014,195	F	Legal	Legal		0	0	0	0	0.000	0.000	0.000
California	2005	35,827,943	R	Legal	Legal	2	0	0	0	0	0.000	0.000	0.000
California	2006	36,021,202	R	Legal	Legal	2	0	0	0	0	0.000	0.000	0.000
California	2007	36,250,311	R	Legal	Legal	2	1	11	0	0	0.028	0.303	0.000
California	2008	36,604,337	R	Legal	Legal	2	1	16	0	0	0.027	0.437	0.000
California	2009	36,961,229	R	Legal	Legal	2	0	0	0	0	0.000	0.000	0.000
California	2010	37,319,550	R	Legal	Legal	3	0	0	0	0	0.000	0.000	0.000
California	2011	37,636,311	R	Legal	Legal	3	1	5	3	0	0.027	0.133	0.080
California	2012	37,944,551	R	Legal	Legal	3	1	33	2	0	0.026	0.870	0.053
California	2013	38,253,768	R	Legal	Legal	5	0	0	0	0	0.000	0.000	0.000
California	2014	38,586,706	R	Legal	Legal	5	0	0	0	0	0.000	0.000	0.000
California	2015	38,904,296	R	Legal	Legal	5	1	8	2	0	0.026	0.206	0.051
California	2016	39,149,186	R	Legal	Legal	5	1	10	4	0	0.026	0.255	0.102
California	2017	39,337,785	R	Legal	Legal	5	0	0	0	0	0.000	0.000	0.000
California	2018	39,437,463	R	Legal	Legal	6	0	0	0	0	0.000	0.000	0.000
California	2019	39,437,610	R	Legal	Legal	6	0	0	0	0	0.000	0.000	0.000
California	2020	39,501,653	R	Legal	Legal	6	0	0	0	0	0.000	0.000	0.000
Colorado	2005	4,631,888	H	Legal	Legal	7	2	27	0	0	0.432	5.829	0.000
Colorado	2006	4,720,423	H	Legal	Legal	4	1	5	4	0	0.212	1.059	0.847
Colorado	2007	4,803,868	H	Legal	Legal	7	0	0	0	0	0.000	0.000	0.000
Colorado	2008	4,889,730	H	Legal	Legal	20	0	0	0	0	0.000	0.000	0.000
Colorado	2009	4,972,195	H	Legal	Legal	16	1	81	1	0	0.201	16.291	0.201
Colorado	2010	5,047,539	H	Legal	Legal	28	0	0	0	0	0.000	0.000	0.000
Colorado	2011	5,121,900	H	Legal	Legal	22	0	0	0	0	0.000	0.000	0.000
Colorado	2012	5,193,660	H	Legal	Legal	17	1	2	0	0	0.193	0.385	0.000
Colorado	2013	5,270,774	H	Legal	Legal	19	1	32	1	0	0.190	6.071	0.190
Colorado	2014	5,352,637	H	Legal	Legal	18	0	0	0	0	0.000	0.000	0.000
Colorado	2015	5,454,328	H	Legal	Legal	12	0	0	0	0	0.000	0.000	0.000
Colorado	2016	5,543,844	H	Legal	Legal	20	1	17	1	0	0.180	3.066	0.180
Colorado	2017	5,617,421	H	Legal	Legal	11	0	0	0	0	0.000	0.000	0.000
Colorado	2018	5,697,155	H	Legal	Legal	9	0	0	0	0	0.000	0.000	0.000
Colorado	2019	5,758,486	H	Legal	Legal	11	0	0	0	0	0.000	0.000	0.000

State	Year	Population	Legal Class	Def 1	Def 2	Number Licenses	Outbreaks	Illnesses	Hospitalizations	Deaths	Outbreak Rate Per 1MM	Illnesses Rate Per 1MM	Hospitalization Rate Per 1MM
Colorado	2020	5,784,865	H	Legal	Legal	4	0	0	0	0	0.000	0.000	0.000
Connecticut	2005	3,506,956	R	Legal	Legal		0	0	0	0	0.000	0.000	0.000
Connecticut	2006	3,517,460	R	Legal	Legal		0	0	0	0	0.000	0.000	0.000
Connecticut	2007	3,527,270	R	Legal	Legal		0	0	0	0	0.000	0.000	0.000
Connecticut	2008	3,545,579	R	Legal	Legal		1	14	5	0	0.282	3.949	1.410
Connecticut	2009	3,561,807	R	Legal	Legal		0	0	0	0	0.000	0.000	0.000
Connecticut	2010	3,579,173	R	Legal	Legal		0	0	0	0	0.000	0.000	0.000
Connecticut	2011	3,588,632	R	Legal	Legal		0	0	0	0	0.000	0.000	0.000
Connecticut	2012	3,595,211	R	Legal	Legal		0	0	0	0	0.000	0.000	0.000
Connecticut	2013	3,595,792	R	Legal	Legal		1	7	1	0	0.278	1.947	0.278
Connecticut	2014	3,595,697	R	Legal	Legal		0	0	0	0	0.000	0.000	0.000
Connecticut	2015	3,588,561	R	Legal	Legal		0	0	0	0	0.000	0.000	0.000
Connecticut	2016	3,579,830	R	Legal	Legal		0	0	0	0	0.000	0.000	0.000
Connecticut	2017	3,575,324	R	Legal	Legal		0	0	0	0	0.000	0.000	0.000
Connecticut	2018	3,574,561	R	Legal	Legal		0	0	0	0	0.000	0.000	0.000
Connecticut	2019	3,566,022	R	Legal	Legal		0	0	0	0	0.000	0.000	0.000
Connecticut	2020	3,597,362	R	Legal	Legal		0	0	0	0	0.000	0.000	0.000
Delaware	2005	845,150	I	Illegal	Illegal		0	0	0	0	0.000	0.000	0.000
Delaware	2006	859,268	I	Illegal	Illegal		0	0	0	0	0.000	0.000	0.000
Delaware	2007	871,749	I	Illegal	Illegal		0	0	0	0	0.000	0.000	0.000
Delaware	2008	883,874	I	Illegal	Illegal		0	0	0	0	0.000	0.000	0.000
Delaware	2009	891,730	I	Illegal	Illegal		0	0	0	0	0.000	0.000	0.000
Delaware	2010	899,647	I	Illegal	Illegal		0	0	0	0	0.000	0.000	0.000
Delaware	2011	907,590	I	Illegal	Illegal		0	0	0	0	0.000	0.000	0.000
Delaware	2012	915,518	I	Illegal	Illegal		0	0	0	0	0.000	0.000	0.000
Delaware	2013	924,062	I	Illegal	Illegal		0	0	0	0	0.000	0.000	0.000
Delaware	2014	933,131	I	Illegal	Illegal		0	0	0	0	0.000	0.000	0.000
Delaware	2015	942,065	I	Illegal	Illegal		0	0	0	0	0.000	0.000	0.000
Delaware	2016	949,989	I	Illegal	Illegal		0	0	0	0	0.000	0.000	0.000
Delaware	2017	957,942	I	Illegal	Illegal		0	0	0	0	0.000	0.000	0.000
Delaware	2018	966,985	I	Illegal	Illegal		0	0	0	0	0.000	0.000	0.000
Delaware	2019	976,668	I	Illegal	Illegal		0	0	0	0	0.000	0.000	0.000
Delaware	2020	992,114	I	Illegal	Illegal		0	0	0	0	0.000	0.000	0.000
District of Columbia	2005	567,136	I	Illegal	Illegal		0	0	0	0	0.000	0.000	0.000

State	Year	Population	Legal Class	Def 1	Def 2	Number Licenses	Outbreaks	Illnesses	Hospitalizations	Deaths	Outbreak Rate Per 1MM	Illnesses Rate Per 1MM	Hospitalization Rate Per 1MM
District of Columbia	2006	570,681	I	Illegal	Illegal		0	0	0	0	0.000	0.000	0.000
District of Columbia	2007	574,404	I	Illegal	Illegal		0	0	0	0	0.000	0.000	0.000
District of Columbia	2008	580,236	I	Illegal	Illegal		0	0	0	0	0.000	0.000	0.000
District of Columbia	2009	592,228	I	Illegal	Illegal		0	0	0	0	0.000	0.000	0.000
District of Columbia	2010	605,282	I	Illegal	Illegal		0	0	0	0	0.000	0.000	0.000
District of Columbia	2011	620,290	I	Illegal	Illegal		0	0	0	0	0.000	0.000	0.000
District of Columbia	2012	635,737	I	Illegal	Illegal		0	0	0	0	0.000	0.000	0.000
District of Columbia	2013	651,559	I	Illegal	Illegal		0	0	0	0	0.000	0.000	0.000
District of Columbia	2014	663,603	I	Illegal	Illegal		0	0	0	0	0.000	0.000	0.000
District of Columbia	2015	677,014	I	Illegal	Illegal		0	0	0	0	0.000	0.000	0.000
District of Columbia	2016	687,576	I	Illegal	Illegal		0	0	0	0	0.000	0.000	0.000
District of Columbia	2017	697,079	I	Illegal	Illegal		0	0	0	0	0.000	0.000	0.000
District of Columbia	2018	704,147	I	Illegal	Illegal		0	0	0	0	0.000	0.000	0.000
District of Columbia	2019	708,253	I	Illegal	Illegal		0	0	0	0	0.000	0.000	0.000
District of Columbia	2020	670,868	I	Illegal	Illegal		0	0	0	0	0.000	0.000	0.000
Florida	2005	17,842,038	P	Legal	Illegal		0	0	0	0	0.000	0.000	0.000
Florida	2006	18,166,990	P	Legal	Illegal		0	0	0	0	0.000	0.000	0.000
Florida	2007	18,367,842	P	Legal	Illegal		0	0	0	0	0.000	0.000	0.000
Florida	2008	18,527,305	P	Legal	Illegal		0	0	0	0	0.000	0.000	0.000
Florida	2009	18,652,644	P	Legal	Illegal		0	0	0	0	0.000	0.000	0.000
Florida	2010	18,846,143	P	Legal	Illegal		0	0	0	0	0.000	0.000	0.000
Florida	2011	19,055,607	P	Legal	Illegal		0	0	0	0	0.000	0.000	0.000
Florida	2012	19,302,016	P	Legal	Illegal		0	0	0	0	0.000	0.000	0.000
Florida	2013	19,551,678	P	Legal	Illegal		0	0	0	0	0.000	0.000	0.000
Florida	2014	19,853,880	P	Legal	Illegal		0	0	0	0	0.000	0.000	0.000
Florida	2015	20,219,111	P	Legal	Illegal		0	0	0	0	0.000	0.000	0.000
Florida	2016	20,627,237	P	Legal	Illegal		1	3	0	0	0.048	0.145	0.000
Florida	2017	20,977,089	P	Legal	Illegal		0	0	0	0	0.000	0.000	0.000
Florida	2018	21,254,926	P	Legal	Illegal		0	0	0	0	0.000	0.000	0.000
Florida	2019	21,492,056	P	Legal	Illegal		0	0	0	0	0.000	0.000	0.000
Florida	2020	21,589,602	P	Legal	Illegal		0	0	0	0	0.000	0.000	0.000
Georgia	2005	8,925,922	P	Legal	Illegal		0	0	0	0	0.000	0.000	0.000
Georgia	2006	9,155,813	P	Legal	Illegal		0	0	0	0	0.000	0.000	0.000
Georgia	2007	9,349,988	P	Legal	Illegal		1	8	0	0	0.107	0.856	0.000

State	Year	Population	Legal Class	Def 1	Def 2	Number Licenses	Outbreaks	Illnesses	Hospitalizations	Deaths	Outbreak Rate Per 1MM	Illnesses Rate Per 1MM	Hospitalization Rate Per 1MM
Georgia	2008	9,504,843	P	Legal	Illegal		0	0	0	0	0.000	0.000	0.000
Georgia	2009	9,620,846	P	Legal	Illegal		0	0	0	0	0.000	0.000	0.000
Georgia	2010	9,712,209	P	Legal	Illegal		0	0	0	0	0.000	0.000	0.000
Georgia	2011	9,803,630	P	Legal	Illegal		0	0	0	0	0.000	0.000	0.000
Georgia	2012	9,903,580	P	Legal	Illegal		0	0	0	0	0.000	0.000	0.000
Georgia	2013	9,975,592	P	Legal	Illegal		0	0	0	0	0.000	0.000	0.000
Georgia	2014	10,071,204	P	Legal	Illegal		0	0	0	0	0.000	0.000	0.000
Georgia	2015	10,183,353	P	Legal	Illegal		0	0	0	0	0.000	0.000	0.000
Georgia	2016	10,308,442	P	Legal	Illegal		0	0	0	0	0.000	0.000	0.000
Georgia	2017	10,417,031	P	Legal	Illegal		0	0	0	0	0.000	0.000	0.000
Georgia	2018	10,519,389	P	Legal	Illegal		0	0	0	0	0.000	0.000	0.000
Georgia	2019	10,628,020	P	Legal	Illegal		0	0	0	0	0.000	0.000	0.000
Georgia	2020	10,729,828	P	Legal	Illegal		0	0	0	0	0.000	0.000	0.000
Hawaii	2005	1,292,729	I	Illegal	Illegal		0	0	0	0	0.000	0.000	0.000
Hawaii	2006	1,309,731	I	Illegal	Illegal		0	0	0	0	0.000	0.000	0.000
Hawaii	2007	1,315,675	I	Illegal	Illegal		0	0	0	0	0.000	0.000	0.000
Hawaii	2008	1,332,213	I	Illegal	Illegal		0	0	0	0	0.000	0.000	0.000
Hawaii	2009	1,346,717	I	Illegal	Illegal		0	0	0	0	0.000	0.000	0.000
Hawaii	2010	1,364,004	I	Illegal	Illegal		0	0	0	0	0.000	0.000	0.000
Hawaii	2011	1,379,562	I	Illegal	Illegal		0	0	0	0	0.000	0.000	0.000
Hawaii	2012	1,395,199	I	Illegal	Illegal		0	0	0	0	0.000	0.000	0.000
Hawaii	2013	1,408,822	I	Illegal	Illegal		0	0	0	0	0.000	0.000	0.000
Hawaii	2014	1,415,335	I	Illegal	Illegal		0	0	0	0	0.000	0.000	0.000
Hawaii	2015	1,422,999	I	Illegal	Illegal		0	0	0	0	0.000	0.000	0.000
Hawaii	2016	1,428,885	I	Illegal	Illegal		0	0	0	0	0.000	0.000	0.000
Hawaii	2017	1,425,763	I	Illegal	Illegal		0	0	0	0	0.000	0.000	0.000
Hawaii	2018	1,423,102	I	Illegal	Illegal		0	0	0	0	0.000	0.000	0.000
Hawaii	2019	1,415,615	I	Illegal	Illegal		0	0	0	0	0.000	0.000	0.000
Hawaii	2020	1,451,043	I	Illegal	Illegal		0	0	0	0	0.000	0.000	0.000
Idaho	2005	1,428,241	R	Legal	Legal		0	0	0	0	0.000	0.000	0.000
Idaho	2006	1,468,669	R	Legal	Legal		1	4	0	0	0.681	2.724	0.000
Idaho	2007	1,505,105	R	Legal	Legal		0	0	0	0	0.000	0.000	0.000
Idaho	2008	1,534,320	R	Legal	Legal		1	5	0	0	0.652	3.259	0.000
Idaho	2009	1,554,439	R	Legal	Legal		0	0	0	0	0.000	0.000	0.000

State	Year	Population	Legal Class	Def 1	Def 2	Number Licenses	Outbreaks	Illnesses	Hospitalizations	Deaths	Outbreak Rate Per 1MM	Illnesses Rate Per 1MM	Hospitalization Rate Per 1MM
Idaho	2010	1,570,819	R	Legal	Legal		0	0	0	0	0.000	0.000	0.000
Idaho	2011	1,584,272	R	Legal	Legal		0	0	0	0	0.000	0.000	0.000
Idaho	2012	1,595,910	R	Legal	Legal		0	0	0	0	0.000	0.000	0.000
Idaho	2013	1,612,053	R	Legal	Legal		0	0	0	0	0.000	0.000	0.000
Idaho	2014	1,632,248	R	Legal	Legal		1	4	1	0	0.613	2.451	0.613
Idaho	2015	1,652,495	R	Legal	Legal		1	15	3	0	0.605	9.077	1.815
Idaho	2016	1,684,036	R	Legal	Legal		0	0	0	0	0.000	0.000	0.000
Idaho	2017	1,719,745	R	Legal	Legal		1	2	0	0	0.581	1.163	0.000
Idaho	2018	1,752,074	R	Legal	Legal		1	2	0	0	0.571	1.142	0.000
Idaho	2019	1,789,060	R	Legal	Legal		0	0	0	0	0.000	0.000	0.000
Idaho	2020	1,849,202	R	Legal	Legal		0	0	0	0	0.000	0.000	0.000
Illinois	2005	12,609,903	H	Legal	Legal		0	0	0	0	0.000	0.000	0.000
Illinois	2006	12,643,955	H	Legal	Legal		1	18	0	0	0.079	1.424	0.000
Illinois	2007	12,695,866	H	Legal	Legal		0	0	0	0	0.000	0.000	0.000
Illinois	2008	12,747,038	H	Legal	Legal		0	0	0	0	0.000	0.000	0.000
Illinois	2009	12,796,778	H	Legal	Legal		0	0	0	0	0.000	0.000	0.000
Illinois	2010	12,840,545	H	Legal	Legal		1	2	0	0	0.078	0.156	0.000
Illinois	2011	12,867,783	H	Legal	Legal		0	0	0	0	0.000	0.000	0.000
Illinois	2012	12,883,029	H	Legal	Legal		0	0	0	0	0.000	0.000	0.000
Illinois	2013	12,895,778	H	Legal	Legal		0	0	0	0	0.000	0.000	0.000
Illinois	2014	12,885,092	H	Legal	Legal		0	0	0	0	0.000	0.000	0.000
Illinois	2015	12,859,585	H	Legal	Legal		0	0	0	0	0.000	0.000	0.000
Illinois	2016	12,821,709	F	Legal	Legal		1	7	1	0	0.078	0.546	0.078
Illinois	2017	12,779,893	F	Legal	Legal		1	11	1	0	0.078	0.861	0.078
Illinois	2018	12,724,685	F	Legal	Legal		0	0	0	0	0.000	0.000	0.000
Illinois	2019	12,667,017	F	Legal	Legal		0	0	0	0	0.000	0.000	0.000
Illinois	2020	12,786,580	F	Legal	Legal		0	0	0	0	0.000	0.000	0.000
Indiana	2005	6,278,616	H	Legal	Legal		0	0	0	0	0.000	0.000	0.000
Indiana	2006	6,332,669	H	Legal	Legal		0	0	0	0	0.000	0.000	0.000
Indiana	2007	6,379,599	H	Legal	Legal		0	0	0	0	0.000	0.000	0.000
Indiana	2008	6,424,806	H	Legal	Legal		0	0	0	0	0.000	0.000	0.000
Indiana	2009	6,459,325	H	Legal	Legal		0	0	0	0	0.000	0.000	0.000
Indiana	2010	6,490,555	H	Legal	Legal		1	25	0	0	0.154	3.852	0.000
Indiana	2011	6,517,250	H	Legal	Legal		0	0	0	0	0.000	0.000	0.000

State	Year	Population	Legal Class	Def 1	Def 2	Number Licenses	Outbreaks	Illnesses	Hospitalizations	Deaths	Outbreak Rate Per 1MM	Illnesses Rate Per 1MM	Hospitalization Rate Per 1MM
Indiana	2012	6,538,989	H	Legal	Legal		0	0	0	0	0.000	0.000	0.000
Indiana	2013	6,570,575	H	Legal	Legal		0	0	0	0	0.000	0.000	0.000
Indiana	2014	6,596,019	H	Legal	Legal		0	0	0	0	0.000	0.000	0.000
Indiana	2015	6,611,442	H	Legal	Legal		0	0	0	0	0.000	0.000	0.000
Indiana	2016	6,637,898	H	Legal	Legal		0	0	0	0	0.000	0.000	0.000
Indiana	2017	6,662,068	H	Legal	Legal		0	0	0	0	0.000	0.000	0.000
Indiana	2018	6,698,481	H	Legal	Legal		0	0	0	0	0.000	0.000	0.000
Indiana	2019	6,731,010	H	Legal	Legal		0	0	0	0	0.000	0.000	0.000
Indiana	2020	6,788,799	H	Legal	Legal		0	0	0	0	0.000	0.000	0.000
Iowa	2005	2,964,454	I	Illegal	Illegal		1	33	0	0	0.337	11.132	0.000
Iowa	2006	2,982,644	I	Illegal	Illegal		0	0	0	0	0.000	0.000	0.000
Iowa	2007	2,999,212	I	Illegal	Illegal		0	0	0	0	0.000	0.000	0.000
Iowa	2008	3,016,734	I	Illegal	Illegal		0	0	0	0	0.000	0.000	0.000
Iowa	2009	3,032,870	I	Illegal	Illegal		0	0	0	0	0.000	0.000	0.000
Iowa	2010	3,050,819	I	Illegal	Illegal		0	0	0	0	0.000	0.000	0.000
Iowa	2011	3,066,772	I	Illegal	Illegal		0	0	0	0	0.000	0.000	0.000
Iowa	2012	3,076,844	I	Illegal	Illegal		1	5	0	0	0.325	1.625	0.000
Iowa	2013	3,093,935	I	Illegal	Illegal		0	0	0	0	0.000	0.000	0.000
Iowa	2014	3,110,643	I	Illegal	Illegal		0	0	0	0	0.000	0.000	0.000
Iowa	2015	3,122,541	I	Illegal	Illegal		0	0	0	0	0.000	0.000	0.000
Iowa	2016	3,133,210	I	Illegal	Illegal		0	0	0	0	0.000	0.000	0.000
Iowa	2017	3,143,734	I	Illegal	Illegal		0	0	0	0	0.000	0.000	0.000
Iowa	2018	3,149,900	I	Illegal	Illegal		0	0	0	0	0.000	0.000	0.000
Iowa	2019	3,159,596	I	Illegal	Illegal		0	0	0	0	0.000	0.000	0.000
Iowa	2020	3,190,571	I	Illegal	Illegal		1	9	0	0	0.313	2.821	0.000
Kansas	2005	2,745,299	F	Legal	Legal		1	4	0	0	0.364	1.457	0.000
Kansas	2006	2,762,931	F	Legal	Legal		0	0	0	0	0.000	0.000	0.000
Kansas	2007	2,783,785	F	Legal	Legal		0	0	0	0	0.000	0.000	0.000
Kansas	2008	2,808,076	F	Legal	Legal		0	0	0	0	0.000	0.000	0.000
Kansas	2009	2,832,704	F	Legal	Legal		0	0	0	0	0.000	0.000	0.000
Kansas	2010	2,858,266	F	Legal	Legal		0	0	0	0	0.000	0.000	0.000
Kansas	2011	2,869,677	F	Legal	Legal		0	0	0	0	0.000	0.000	0.000
Kansas	2012	2,886,024	F	Legal	Legal		0	0	0	0	0.000	0.000	0.000
Kansas	2013	2,894,306	F	Legal	Legal		0	0	0	0	0.000	0.000	0.000

State	Year	Population	Legal Class	Def 1	Def 2	Number Licenses	Outbreaks	Illnesses	Hospitalizations	Deaths	Outbreak Rate Per 1MM	Illnesses Rate Per 1MM	Hospitalization Rate Per 1MM
Kansas	2014	2,901,861	F	Legal	Legal		0	0	0	0	0.000	0.000	0.000
Kansas	2015	2,910,717	F	Legal	Legal		0	0	0	0	0.000	0.000	0.000
Kansas	2016	2,912,977	F	Legal	Legal		0	0	0	0	0.000	0.000	0.000
Kansas	2017	2,910,892	F	Legal	Legal		0	0	0	0	0.000	0.000	0.000
Kansas	2018	2,912,748	F	Legal	Legal		0	0	0	0	0.000	0.000	0.000
Kansas	2019	2,912,635	F	Legal	Legal		0	0	0	0	0.000	0.000	0.000
Kansas	2020	2,937,919	F	Legal	Legal		0	0	0	0	0.000	0.000	0.000
Kentucky	2005	4,182,742	I	Illegal	Illegal		0	0	0	0	0.000	0.000	0.000
Kentucky	2006	4,219,239	I	Illegal	Illegal		0	0	0	0	0.000	0.000	0.000
Kentucky	2007	4,256,672	I	Illegal	Illegal		0	0	0	0	0.000	0.000	0.000
Kentucky	2008	4,289,878	I	Illegal	Illegal		0	0	0	0	0.000	0.000	0.000
Kentucky	2009	4,317,074	I	Illegal	Illegal		0	0	0	0	0.000	0.000	0.000
Kentucky	2010	4,348,464	I	Illegal	Illegal		0	0	0	0	0.000	0.000	0.000
Kentucky	2011	4,370,817	H	Legal	Legal		0	0	0	0	0.000	0.000	0.000
Kentucky	2012	4,387,865	H	Legal	Legal		0	0	0	0	0.000	0.000	0.000
Kentucky	2013	4,406,906	H	Legal	Legal		0	0	0	0	0.000	0.000	0.000
Kentucky	2014	4,416,992	H	Legal	Legal		1	4	4	0	0.226	0.906	0.906
Kentucky	2015	4,429,126	H	Legal	Legal		0	0	0	0	0.000	0.000	0.000
Kentucky	2016	4,440,306	H	Legal	Legal		0	0	0	0	0.000	0.000	0.000
Kentucky	2017	4,455,590	H	Legal	Legal		0	0	0	0	0.000	0.000	0.000
Kentucky	2018	4,464,273	H	Legal	Legal		0	0	0	0	0.000	0.000	0.000
Kentucky	2019	4,472,345	H	Legal	Legal		0	0	0	0	0.000	0.000	0.000
Kentucky	2020	4,507,445	H	Legal	Legal		0	0	0	0	0.000	0.000	0.000
Louisiana	2005	4,576,628	I	Illegal	Illegal		0	0	0	0	0.000	0.000	0.000
Louisiana	2006	4,302,665	I	Illegal	Illegal		0	0	0	0	0.000	0.000	0.000
Louisiana	2007	4,375,581	I	Illegal	Illegal		0	0	0	0	0.000	0.000	0.000
Louisiana	2008	4,435,586	I	Illegal	Illegal		0	0	0	0	0.000	0.000	0.000
Louisiana	2009	4,491,648	I	Illegal	Illegal		0	0	0	0	0.000	0.000	0.000
Louisiana	2010	4,544,635	I	Illegal	Illegal		0	0	0	0	0.000	0.000	0.000
Louisiana	2011	4,576,244	I	Illegal	Illegal		0	0	0	0	0.000	0.000	0.000
Louisiana	2012	4,602,067	I	Illegal	Illegal		0	0	0	0	0.000	0.000	0.000
Louisiana	2013	4,626,040	I	Illegal	Illegal		0	0	0	0	0.000	0.000	0.000
Louisiana	2014	4,645,938	I	Illegal	Illegal		0	0	0	0	0.000	0.000	0.000
Louisiana	2015	4,666,998	I	Illegal	Illegal		0	0	0	0	0.000	0.000	0.000

State	Year	Population	Legal Class	Def 1	Def 2	Number Licenses	Outbreaks	Illnesses	Hospitalizations	Deaths	Outbreak Rate Per 1MM	Illnesses Rate Per 1MM	Hospitalization Rate Per 1MM
Louisiana	2016	4,681,346	I	Illegal	Illegal		0	0	0	0	0.000	0.000	0.000
Louisiana	2017	4,673,673	I	Illegal	Illegal		0	0	0	0	0.000	0.000	0.000
Louisiana	2018	4,664,450	I	Illegal	Illegal		0	0	0	0	0.000	0.000	0.000
Louisiana	2019	4,658,285	I	Illegal	Illegal		0	0	0	0	0.000	0.000	0.000
Louisiana	2020	4,651,664	I	Illegal	Illegal		0	0	0	0	0.000	0.000	0.000
Maine	2005	1,318,787	R	Legal	Legal	16	0	0	0	0	0.000	0.000	0.000
Maine	2006	1,323,619	R	Legal	Legal	15	0	0	0	0	0.000	0.000	0.000
Maine	2007	1,327,040	R	Legal	Legal	18	0	0	0	0	0.000	0.000	0.000
Maine	2008	1,330,509	R	Legal	Legal	29	0	0	0	0	0.000	0.000	0.000
Maine	2009	1,329,590	R	Legal	Legal	38	0	0	0	0	0.000	0.000	0.000
Maine	2010	1,327,651	R	Legal	Legal	42	0	0	0	0	0.000	0.000	0.000
Maine	2011	1,328,473	R	Legal	Legal	50	0	0	0	0	0.000	0.000	0.000
Maine	2012	1,328,094	R	Legal	Legal	57	0	0	0	0	0.000	0.000	0.000
Maine	2013	1,328,543	R	Legal	Legal	60	0	0	0	0	0.000	0.000	0.000
Maine	2014	1,331,217	R	Legal	Legal	60	1	4	0	0	0.751	3.005	0.000
Maine	2015	1,329,098	R	Legal	Legal	62	0	0	0	0	0.000	0.000	0.000
Maine	2016	1,332,348	R	Legal	Legal	67	0	0	0	0	0.000	0.000	0.000
Maine	2017	1,335,743	R	Legal	Legal	74	0	0	0	0	0.000	0.000	0.000
Maine	2018	1,340,123	R	Legal	Legal	61	0	0	0	0	0.000	0.000	0.000
Maine	2019	1,345,770	R	Legal	Legal	55	0	0	0	0	0.000	0.000	0.000
Maine	2020	1,363,557	R	Legal	Legal	59	1	4	0	0	0.733	2.934	0.000
Maryland	2005	5,592,379	I	Illegal	Illegal		0	0	0	0	0.000	0.000	0.000
Maryland	2006	5,627,367	I	Illegal	Illegal		0	0	0	0	0.000	0.000	0.000
Maryland	2007	5,653,408	I	Illegal	Illegal		0	0	0	0	0.000	0.000	0.000
Maryland	2008	5,684,965	I	Illegal	Illegal		0	0	0	0	0.000	0.000	0.000
Maryland	2009	5,730,388	I	Illegal	Illegal		0	0	0	0	0.000	0.000	0.000
Maryland	2010	5,788,784	I	Illegal	Illegal		0	0	0	0	0.000	0.000	0.000
Maryland	2011	5,840,241	I	Illegal	Illegal		0	0	0	0	0.000	0.000	0.000
Maryland	2012	5,888,375	I	Illegal	Illegal		0	0	0	0	0.000	0.000	0.000
Maryland	2013	5,925,197	I	Illegal	Illegal		0	0	0	0	0.000	0.000	0.000
Maryland	2014	5,960,064	I	Illegal	Illegal		0	0	0	0	0.000	0.000	0.000
Maryland	2015	5,988,528	I	Illegal	Illegal		0	0	0	0	0.000	0.000	0.000
Maryland	2016	6,007,014	P	Legal	Illegal		0	0	0	0	0.000	0.000	0.000
Maryland	2017	6,028,186	P	Legal	Illegal		0	0	0	0	0.000	0.000	0.000

State	Year	Population	Legal Class	Def 1	Def 2	Number Licenses	Outbreaks	Illnesses	Hospitalizations	Deaths	Outbreak Rate Per 1MM	Illnesses Rate Per 1MM	Hospitalization Rate Per 1MM
Maryland	2018	6,042,153	P	Legal	Illegal		0	0	0	0	0.000	0.000	0.000
Maryland	2019	6,054,954	P	Legal	Illegal		0	0	0	0	0.000	0.000	0.000
Maryland	2020	6,173,205	P	Legal	Illegal		0	0	0	0	0.000	0.000	0.000
Massachusetts	2005	6,403,290	F	Legal	Legal	6	0	0	0	0	0.000	0.000	0.000
Massachusetts	2006	6,410,084	F	Legal	Legal	7	0	0	0	0	0.000	0.000	0.000
Massachusetts	2007	6,431,559	F	Legal	Legal	8	0	0	0	0	0.000	0.000	0.000
Massachusetts	2008	6,468,967	F	Legal	Legal	9	1	8	0	0	0.155	1.237	0.000
Massachusetts	2009	6,517,613	F	Legal	Legal	10	0	0	0	0	0.000	0.000	0.000
Massachusetts	2010	6,566,440	F	Legal	Legal	10	1	2	1	0	0.152	0.305	0.152
Massachusetts	2011	6,614,218	F	Legal	Legal	11	0	0	0	0	0.000	0.000	0.000
Massachusetts	2012	6,664,269	F	Legal	Legal	11	0	0	0	0	0.000	0.000	0.000
Massachusetts	2013	6,715,158	F	Legal	Legal	12	0	0	0	0	0.000	0.000	0.000
Massachusetts	2014	6,764,864	F	Legal	Legal	16	0	0	0	0	0.000	0.000	0.000
Massachusetts	2015	6,797,484	F	Legal	Legal	17	1	2	1	0	0.147	0.294	0.147
Massachusetts	2016	6,827,280	F	Legal	Legal	18	1	2	1	0	0.146	0.293	0.146
Massachusetts	2017	6,863,560	F	Legal	Legal	20	0	0	0	0	0.000	0.000	0.000
Massachusetts	2018	6,885,720	F	Legal	Legal	20	0	0	0	0	0.000	0.000	0.000
Massachusetts	2019	6,894,883	F	Legal	Legal	21	0	0	0	0	0.000	0.000	0.000
Massachusetts	2020	6,995,729	F	Legal	Legal	21	0	0	0	0	0.000	0.000	0.000
Michigan	2005	10,051,137	I	Illegal	Illegal		0	0	0	0	0.000	0.000	0.000
Michigan	2006	10,036,081	I	Illegal	Illegal		0	0	0	0	0.000	0.000	0.000
Michigan	2007	10,001,284	I	Illegal	Illegal		0	0	0	0	0.000	0.000	0.000
Michigan	2008	9,946,889	I	Illegal	Illegal		0	0	0	0	0.000	0.000	0.000
Michigan	2009	9,901,591	I	Illegal	Illegal		0	0	0	0	0.000	0.000	0.000
Michigan	2010	9,877,597	I	Illegal	Illegal		1	11	0	0	0.101	1.114	0.000
Michigan	2011	9,883,053	I	Illegal	Illegal		2	7	1	0	0.202	0.708	0.101
Michigan	2012	9,898,289	I	Illegal	Illegal		0	0	0	0	0.000	0.000	0.000
Michigan	2013	9,914,802	H	Legal	Legal		0	0	0	0	0.000	0.000	0.000
Michigan	2014	9,932,033	H	Legal	Legal		1	2	0	0	0.101	0.201	0.000
Michigan	2015	9,934,483	H	Legal	Legal		1	6	1	0	0.101	0.604	0.101
Michigan	2016	9,954,117	H	Legal	Legal		1	4	2	0	0.100	0.402	0.201
Michigan	2017	9,976,752	H	Legal	Legal		0	0	0	0	0.000	0.000	0.000
Michigan	2018	9,987,286	H	Legal	Legal		0	0	0	0	0.000	0.000	0.000
Michigan	2019	9,984,795	H	Legal	Legal		0	0	0	0	0.000	0.000	0.000

State	Year	Population	Legal Class	Def 1	Def 2	Number Licenses	Outbreaks	Illnesses	Hospitalizations	Deaths	Outbreak Rate Per 1MM	Illnesses Rate Per 1MM	Hospitalization Rate Per 1MM
Michigan	2020	10,069,577	H	Legal	Legal		0	0	0	0	0.000	0.000	0.000
Minnesota	2005	5,119,598	F	Legal	Legal		0	0	0	0	0.000	0.000	0.000
Minnesota	2006	5,163,555	F	Legal	Legal		0	0	0	0	0.000	0.000	0.000
Minnesota	2007	5,207,203	F	Legal	Legal		0	0	0	0	0.000	0.000	0.000
Minnesota	2008	5,247,018	F	Legal	Legal		2	4	2	0	0.381	0.762	0.381
Minnesota	2009	5,281,203	F	Legal	Legal		0	0	0	0	0.000	0.000	0.000
Minnesota	2010	5,310,934	F	Legal	Legal		2	15	5	0	0.377	2.824	0.941
Minnesota	2011	5,346,620	F	Legal	Legal		2	5	2	0	0.374	0.935	0.374
Minnesota	2012	5,377,500	F	Legal	Legal		1	7	0	0	0.186	1.302	0.000
Minnesota	2013	5,414,722	F	Legal	Legal		1	6	0	0	0.185	1.108	0.000
Minnesota	2014	5,452,665	F	Legal	Legal		1	9	2	0	0.183	1.651	0.367
Minnesota	2015	5,484,002	F	Legal	Legal		0	0	0	0	0.000	0.000	0.000
Minnesota	2016	5,525,360	F	Legal	Legal		0	0	0	0	0.000	0.000	0.000
Minnesota	2017	5,569,283	F	Legal	Legal		0	0	0	0	0.000	0.000	0.000
Minnesota	2018	5,608,762	F	Legal	Legal		0	0	0	0	0.000	0.000	0.000
Minnesota	2019	5,640,053	F	Legal	Legal		1	5	0	0	0.177	0.887	0.000
Minnesota	2020	5,709,852	F	Legal	Legal		0	0	0	0	0.000	0.000	0.000
Mississippi	2005	2,905,943	F	Legal	Legal		0	0	0	0	0.000	0.000	0.000
Mississippi	2006	2,904,978	F	Legal	Legal		0	0	0	0	0.000	0.000	0.000
Mississippi	2007	2,928,350	F	Legal	Legal		0	0	0	0	0.000	0.000	0.000
Mississippi	2008	2,947,806	F	Legal	Legal		0	0	0	0	0.000	0.000	0.000
Mississippi	2009	2,958,774	F	Legal	Legal		0	0	0	0	0.000	0.000	0.000
Mississippi	2010	2,970,615	F	Legal	Legal		0	0	0	0	0.000	0.000	0.000
Mississippi	2011	2,979,147	F	Legal	Legal		0	0	0	0	0.000	0.000	0.000
Mississippi	2012	2,984,599	F	Legal	Legal		0	0	0	0	0.000	0.000	0.000
Mississippi	2013	2,989,839	F	Legal	Legal		0	0	0	0	0.000	0.000	0.000
Mississippi	2014	2,991,892	F	Legal	Legal		0	0	0	0	0.000	0.000	0.000
Mississippi	2015	2,990,231	F	Legal	Legal		0	0	0	0	0.000	0.000	0.000
Mississippi	2016	2,990,595	F	Legal	Legal		0	0	0	0	0.000	0.000	0.000
Mississippi	2017	2,990,674	F	Legal	Legal		0	0	0	0	0.000	0.000	0.000
Mississippi	2018	2,982,879	F	Legal	Legal		0	0	0	0	0.000	0.000	0.000
Mississippi	2019	2,978,227	F	Legal	Legal		0	0	0	0	0.000	0.000	0.000
Mississippi	2020	2,958,141	F	Legal	Legal		0	0	0	0	0.000	0.000	0.000
Missouri	2005	5,790,300	F	Legal	Legal		0	0	0	0	0.000	0.000	0.000

State	Year	Population	Legal Class	Def 1	Def 2	Number Licenses	Outbreaks	Illnesses	Hospitalizations	Deaths	Outbreak Rate Per 1MM	Illnesses Rate Per 1MM	Hospitalization Rate Per 1MM
Missouri	2006	5,842,704	F	Legal	Legal		0	0	0	0	0.000	0.000	0.000
Missouri	2007	5,887,612	F	Legal	Legal		0	0	0	0	0.000	0.000	0.000
Missouri	2008	5,923,916	F	Legal	Legal		0	0	0	0	0.000	0.000	0.000
Missouri	2009	5,961,088	F	Legal	Legal		0	0	0	0	0.000	0.000	0.000
Missouri	2010	5,996,089	F	Legal	Legal		0	0	0	0	0.000	0.000	0.000
Missouri	2011	6,011,182	F	Legal	Legal		0	0	0	0	0.000	0.000	0.000
Missouri	2012	6,026,027	F	Legal	Legal		0	0	0	0	0.000	0.000	0.000
Missouri	2013	6,042,989	F	Legal	Legal		0	0	0	0	0.000	0.000	0.000
Missouri	2014	6,059,130	F	Legal	Legal		0	0	0	0	0.000	0.000	0.000
Missouri	2015	6,075,411	F	Legal	Legal		0	0	0	0	0.000	0.000	0.000
Missouri	2016	6,091,384	F	Legal	Legal		0	0	0	0	0.000	0.000	0.000
Missouri	2017	6,111,382	F	Legal	Legal		1	13	0	0	0.164	2.127	0.000
Missouri	2018	6,125,986	F	Legal	Legal		0	0	0	0	0.000	0.000	0.000
Missouri	2019	6,140,475	F	Legal	Legal		0	0	0	0	0.000	0.000	0.000
Missouri	2020	6,153,998	F	Legal	Legal		0	0	0	0	0.000	0.000	0.000
Montana	2005	940,102	I	Illegal	Illegal		0	0	0	0	0.000	0.000	0.000
Montana	2006	952,692	I	Illegal	Illegal		0	0	0	0	0.000	0.000	0.000
Montana	2007	964,706	I	Illegal	Illegal		0	0	0	0	0.000	0.000	0.000
Montana	2008	976,415	I	Illegal	Illegal		0	0	0	0	0.000	0.000	0.000
Montana	2009	983,982	I	Illegal	Illegal		0	0	0	0	0.000	0.000	0.000
Montana	2010	990,730	I	Illegal	Illegal		0	0	0	0	0.000	0.000	0.000
Montana	2011	997,518	I	Illegal	Illegal		0	0	0	0	0.000	0.000	0.000
Montana	2012	1,004,168	I	Illegal	Illegal		0	0	0	0	0.000	0.000	0.000
Montana	2013	1,014,158	I	Illegal	Illegal		0	0	0	0	0.000	0.000	0.000
Montana	2014	1,022,657	I	Illegal	Illegal		1	7	3	0	0.978	6.845	2.934
Montana	2015	1,031,495	I	Illegal	Illegal		0	0	0	0	0.000	0.000	0.000
Montana	2016	1,042,137	I	Illegal	Illegal		0	0	0	0	0.000	0.000	0.000
Montana	2017	1,053,862	H	Legal	Legal		0	0	0	0	0.000	0.000	0.000
Montana	2018	1,061,818	H	Legal	Legal		0	0	0	0	0.000	0.000	0.000
Montana	2019	1,070,123	H	Legal	Legal		0	0	0	0	0.000	0.000	0.000
Montana	2020	1,087,075	H	Legal	Legal		0	0	0	0	0.000	0.000	0.000
Multistate	2005						1	18	5	0			
Multistate	2006						0	0	0	0			
Multistate	2007						0	0	0	0			

State	Year	Population	Legal Class	Def 1	Def 2	Number Licenses	Outbreaks	Illnesses	Hospitalizations	Deaths	Outbreak Rate Per 1MM	Illnesses Rate Per 1MM	Hospitalization Rate Per 1MM
Multistate	2008						1	65	1	0			
Multistate	2009						0	0	0	0			
Multistate	2010						0	0	0	0			
Multistate	2011						0	0	0	0			
Multistate	2012						1	16	4	0			
Multistate	2013						0	0	0	0			
Multistate	2014						1	2	2	1			
Multistate	2015						0	0	0	0			
Multistate	2016						0	0	0	0			
Multistate	2017						0	0	0	0			
Multistate	2018						0	0	0	0			
Multistate	2019						0	0	0	0			
Multistate	2020						0	0	0	0			
Nebraska	2005	1,761,497	F	Legal	Legal		0	0	0	0	0.000	0.000	0.000
Nebraska	2006	1,772,693	F	Legal	Legal		0	0	0	0	0.000	0.000	0.000
Nebraska	2007	1,783,440	F	Legal	Legal		0	0	0	0	0.000	0.000	0.000
Nebraska	2008	1,796,378	F	Legal	Legal		0	0	0	0	0.000	0.000	0.000
Nebraska	2009	1,812,683	F	Legal	Legal		0	0	0	0	0.000	0.000	0.000
Nebraska	2010	1,829,591	F	Legal	Legal		0	0	0	0	0.000	0.000	0.000
Nebraska	2011	1,840,914	F	Legal	Legal		0	0	0	0	0.000	0.000	0.000
Nebraska	2012	1,853,691	F	Legal	Legal		0	0	0	0	0.000	0.000	0.000
Nebraska	2013	1,865,813	F	Legal	Legal		0	0	0	0	0.000	0.000	0.000
Nebraska	2014	1,879,955	F	Legal	Legal		0	0	0	0	0.000	0.000	0.000
Nebraska	2015	1,892,059	F	Legal	Legal		0	0	0	0	0.000	0.000	0.000
Nebraska	2016	1,906,483	F	Legal	Legal		0	0	0	0	0.000	0.000	0.000
Nebraska	2017	1,916,998	F	Legal	Legal		0	0	0	0	0.000	0.000	0.000
Nebraska	2018	1,925,512	F	Legal	Legal		0	0	0	0	0.000	0.000	0.000
Nebraska	2019	1,932,571	F	Legal	Legal		0	0	0	0	0.000	0.000	0.000
Nebraska	2020	1,962,642	F	Legal	Legal		0	0	0	0	0.000	0.000	0.000
Nevada	2005	2,432,143	I	Illegal	Illegal		0	0	0	0	0.000	0.000	0.000
Nevada	2006	2,522,658	I	Illegal	Illegal		0	0	0	0	0.000	0.000	0.000
Nevada	2007	2,601,072	I	Illegal	Illegal		0	0	0	0	0.000	0.000	0.000
Nevada	2008	2,653,630	I	Illegal	Illegal		0	0	0	0	0.000	0.000	0.000
Nevada	2009	2,684,665	I	Illegal	Illegal		0	0	0	0	0.000	0.000	0.000

State	Year	Population	Legal Class	Def 1	Def 2	Number Licenses	Outbreaks	Illnesses	Hospitalizations	Deaths	Outbreak Rate Per 1MM	Illnesses Rate Per 1MM	Hospitalization Rate Per 1MM
Nevada	2010	2,702,483	I	Illegal	Illegal		0	0	0	0	0.000	0.000	0.000
Nevada	2011	2,713,114	I	Illegal	Illegal		0	0	0	0	0.000	0.000	0.000
Nevada	2012	2,744,670	I	Illegal	Illegal		0	0	0	0	0.000	0.000	0.000
Nevada	2013	2,776,956	I	Illegal	Illegal		0	0	0	0	0.000	0.000	0.000
Nevada	2014	2,818,935	I	Illegal	Illegal		0	0	0	0	0.000	0.000	0.000
Nevada	2015	2,868,531	I	Illegal	Illegal		0	0	0	0	0.000	0.000	0.000
Nevada	2016	2,919,555	I	Illegal	Illegal		0	0	0	0	0.000	0.000	0.000
Nevada	2017	2,972,097	I	Illegal	Illegal		0	0	0	0	0.000	0.000	0.000
Nevada	2018	3,030,725	I	Illegal	Illegal		0	0	0	0	0.000	0.000	0.000
Nevada	2019	3,090,771	I	Illegal	Illegal		0	0	0	0	0.000	0.000	0.000
Nevada	2020	3,115,648	I	Illegal	Illegal		0	0	0	0	0.000	0.000	0.000
New Hampshire	2005	1,298,492	R	Legal	Legal		0	0	0	0	0.000	0.000	0.000
New Hampshire	2006	1,308,389	R	Legal	Legal		0	0	0	0	0.000	0.000	0.000
New Hampshire	2007	1,312,540	R	Legal	Legal		0	0	0	0	0.000	0.000	0.000
New Hampshire	2008	1,315,906	R	Legal	Legal		0	0	0	0	0.000	0.000	0.000
New Hampshire	2009	1,316,102	R	Legal	Legal		0	0	0	0	0.000	0.000	0.000
New Hampshire	2010	1,316,807	R	Legal	Legal		0	0	0	0	0.000	0.000	0.000
New Hampshire	2011	1,320,444	R	Legal	Legal		0	0	0	0	0.000	0.000	0.000
New Hampshire	2012	1,324,677	R	Legal	Legal		0	0	0	0	0.000	0.000	0.000
New Hampshire	2013	1,327,272	R	Legal	Legal		0	0	0	0	0.000	0.000	0.000
New Hampshire	2014	1,334,257	R	Legal	Legal		0	0	0	0	0.000	0.000	0.000
New Hampshire	2015	1,337,480	R	Legal	Legal		0	0	0	0	0.000	0.000	0.000
New Hampshire	2016	1,343,694	R	Legal	Legal		0	0	0	0	0.000	0.000	0.000
New Hampshire	2017	1,350,395	R	Legal	Legal		0	0	0	0	0.000	0.000	0.000
New Hampshire	2018	1,355,064	R	Legal	Legal		1	3	0	0	0.738	2.214	0.000
New Hampshire	2019	1,360,783	R	Legal	Legal		1	2	1	0	0.735	1.470	0.735
New Hampshire	2020	1,378,587	R	Legal	Legal		0	0	0	0	0.000	0.000	0.000
New Jersey	2005	8,651,974	I	Illegal	Illegal		0	0	0	0	0.000	0.000	0.000
New Jersey	2006	8,661,679	I	Illegal	Illegal		0	0	0	0	0.000	0.000	0.000
New Jersey	2007	8,677,885	I	Illegal	Illegal		0	0	0	0	0.000	0.000	0.000
New Jersey	2008	8,711,090	I	Illegal	Illegal		0	0	0	0	0.000	0.000	0.000
New Jersey	2009	8,755,602	I	Illegal	Illegal		0	0	0	0	0.000	0.000	0.000
New Jersey	2010	8,799,451	I	Illegal	Illegal		0	0	0	0	0.000	0.000	0.000
New Jersey	2011	8,828,552	I	Illegal	Illegal		0	0	0	0	0.000	0.000	0.000

State	Year	Population	Legal Class	Def 1	Def 2	Number Licenses	Outbreaks	Illnesses	Hospitalizations	Deaths	Outbreak Rate Per 1MM	Illnesses Rate Per 1MM	Hospitalization Rate Per 1MM
New Jersey	2012	8,845,671	I	Illegal	Illegal		0	0	0	0	0.000	0.000	0.000
New Jersey	2013	8,857,821	I	Illegal	Illegal		0	0	0	0	0.000	0.000	0.000
New Jersey	2014	8,867,277	I	Illegal	Illegal		0	0	0	0	0.000	0.000	0.000
New Jersey	2015	8,870,312	I	Illegal	Illegal		0	0	0	0	0.000	0.000	0.000
New Jersey	2016	8,873,584	I	Illegal	Illegal		0	0	0	0	0.000	0.000	0.000
New Jersey	2017	8,888,147	I	Illegal	Illegal		0	0	0	0	0.000	0.000	0.000
New Jersey	2018	8,891,730	I	Illegal	Illegal		0	0	0	0	0.000	0.000	0.000
New Jersey	2019	8,891,258	I	Illegal	Illegal		0	0	0	0	0.000	0.000	0.000
New Jersey	2020	9,271,689	I	Illegal	Illegal		0	0	0	0	0.000	0.000	0.000
New Mexico	2005	1,932,274	R	Legal	Legal		0	0	0	0	0.000	0.000	0.000
New Mexico	2006	1,962,137	R	Legal	Legal		0	0	0	0	0.000	0.000	0.000
New Mexico	2007	1,990,070	R	Legal	Legal		0	0	0	0	0.000	0.000	0.000
New Mexico	2008	2,010,662	R	Legal	Legal		0	0	0	0	0.000	0.000	0.000
New Mexico	2009	2,036,802	R	Legal	Legal		0	0	0	0	0.000	0.000	0.000
New Mexico	2010	2,064,614	R	Legal	Legal		0	0	0	0	0.000	0.000	0.000
New Mexico	2011	2,080,707	R	Legal	Legal		0	0	0	0	0.000	0.000	0.000
New Mexico	2012	2,087,715	R	Legal	Legal		0	0	0	0	0.000	0.000	0.000
New Mexico	2013	2,092,833	R	Legal	Legal		0	0	0	0	0.000	0.000	0.000
New Mexico	2014	2,090,236	R	Legal	Legal		0	0	0	0	0.000	0.000	0.000
New Mexico	2015	2,090,071	R	Legal	Legal		0	0	0	0	0.000	0.000	0.000
New Mexico	2016	2,092,555	R	Legal	Legal		1	6	0	0	0.478	2.867	0.000
New Mexico	2017	2,092,844	R	Legal	Legal		0	0	0	0	0.000	0.000	0.000
New Mexico	2018	2,093,754	R	Legal	Legal		0	0	0	0	0.000	0.000	0.000
New Mexico	2019	2,099,634	R	Legal	Legal		0	0	0	0	0.000	0.000	0.000
New Mexico	2020	2,118,390	R	Legal	Legal		0	0	0	0	0.000	0.000	0.000
New York	2005	19,132,610	F	Legal	Legal	10	0	0	0	0	0.000	0.000	0.000
New York	2006	19,104,631	F	Legal	Legal	12	1	2	0	0	0.052	0.105	0.000
New York	2007	19,132,335	F	Legal	Legal	23	1	2	1	0	0.052	0.105	0.052
New York	2008	19,212,436	F	Legal	Legal	0	0	0	0	0	0.000	0.000	0.000
New York	2009	19,307,066	F	Legal	Legal	0	0	0	0	0	0.000	0.000	0.000
New York	2010	19,399,956	F	Legal	Legal	34	1	20	1	0	0.052	1.031	0.052
New York	2011	19,499,921	F	Legal	Legal	36	3	20	0	0	0.154	1.026	0.000
New York	2012	19,574,362	F	Legal	Legal	42	1	6	2	0	0.051	0.307	0.102
New York	2013	19,626,488	F	Legal	Legal	48	0	0	0	0	0.000	0.000	0.000

State	Year	Population	Legal Class	Def 1	Def 2	Number Licenses	Outbreaks	Illnesses	Hospitalizations	Deaths	Outbreak Rate Per 1MM	Illnesses Rate Per 1MM	Hospitalization Rate Per 1MM
New York	2014	19,653,431	F	Legal	Legal	46	1	8	0	0	0.051	0.407	0.000
New York	2015	19,657,321	F	Legal	Legal	45	0	0	0	0	0.000	0.000	0.000
New York	2016	19,636,391	F	Legal	Legal	50	0	0	0	0	0.000	0.000	0.000
New York	2017	19,593,849	F	Legal	Legal	55	0	0	0	0	0.000	0.000	0.000
New York	2018	19,544,098	F	Legal	Legal	59	0	0	0	0	0.000	0.000	0.000
New York	2019	19,463,131	F	Legal	Legal	64	0	0	0	0	0.000	0.000	0.000
New York	2020	20,108,296	F	Legal	Legal	74	0	0	0	0	0.000	0.000	0.000
North Carolina	2005	8,705,407	P	Legal	Illegal		0	0	0	0	0.000	0.000	0.000
North Carolina	2006	8,917,270	P	Legal	Illegal		0	0	0	0	0.000	0.000	0.000
North Carolina	2007	9,118,037	P	Legal	Illegal		0	0	0	0	0.000	0.000	0.000
North Carolina	2008	9,309,449	P	Legal	Illegal		0	0	0	0	0.000	0.000	0.000
North Carolina	2009	9,449,566	P	Legal	Illegal		0	0	0	0	0.000	0.000	0.000
North Carolina	2010	9,574,586	P	Legal	Illegal		0	0	0	0	0.000	0.000	0.000
North Carolina	2011	9,658,913	P	Legal	Illegal		1	3	0	0	0.104	0.311	0.000
North Carolina	2012	9,751,810	P	Legal	Illegal		0	0	0	0	0.000	0.000	0.000
North Carolina	2013	9,846,717	P	Legal	Illegal		0	0	0	0	0.000	0.000	0.000
North Carolina	2014	9,937,295	P	Legal	Illegal		0	0	0	0	0.000	0.000	0.000
North Carolina	2015	10,037,218	P	Legal	Illegal		0	0	0	0	0.000	0.000	0.000
North Carolina	2016	10,161,802	P	Legal	Illegal		0	0	0	0	0.000	0.000	0.000
North Carolina	2017	10,275,758	P	Legal	Illegal		0	0	0	0	0.000	0.000	0.000
North Carolina	2018	10,391,358	H	Legal	Legal		0	0	0	0	0.000	0.000	0.000
North Carolina	2019	10,501,384	H	Legal	Legal		0	0	0	0	0.000	0.000	0.000
North Carolina	2020	10,449,445	H	Legal	Legal		0	0	0	0	0.000	0.000	0.000
North Dakota	2005	646,089	I	Illegal	Illegal		0	0	0	0	0.000	0.000	0.000
North Dakota	2006	649,422	I	Illegal	Illegal		0	0	0	0	0.000	0.000	0.000
North Dakota	2007	652,822	I	Illegal	Illegal		0	0	0	0	0.000	0.000	0.000
North Dakota	2008	657,569	I	Illegal	Illegal		1	3	0	0	1.521	4.562	0.000
North Dakota	2009	664,968	I	Illegal	Illegal		0	0	0	0	0.000	0.000	0.000
North Dakota	2010	674,752	I	Illegal	Illegal		1	7	0	0	1.482	10.374	0.000
North Dakota	2011	685,526	I	Illegal	Illegal		0	0	0	0	0.000	0.000	0.000
North Dakota	2012	702,227	I	Illegal	Illegal		0	0	0	0	0.000	0.000	0.000
North Dakota	2013	723,149	H	Legal	Legal		0	0	0	0	0.000	0.000	0.000
North Dakota	2014	738,736	H	Legal	Legal		0	0	0	0	0.000	0.000	0.000
North Dakota	2015	755,537	H	Legal	Legal		0	0	0	0	0.000	0.000	0.000

State	Year	Population	Legal Class	Def 1	Def 2	Number Licenses	Outbreaks	Illnesses	Hospitalizations	Deaths	Outbreak Rate Per 1MM	Illnesses Rate Per 1MM	Hospitalization Rate Per 1MM
North Dakota	2016	756,114	H	Legal	Legal		0	0	0	0	0.000	0.000	0.000
North Dakota	2017	756,755	H	Legal	Legal		0	0	0	0	0.000	0.000	0.000
North Dakota	2018	760,062	H	Legal	Legal		0	0	0	0	0.000	0.000	0.000
North Dakota	2019	763,724	H	Legal	Legal		0	0	0	0	0.000	0.000	0.000
North Dakota	2020	779,518	H	Legal	Legal		0	0	0	0	0.000	0.000	0.000
Ohio	2005	11,463,320	I	Illegal	Illegal		0	0	0	0	0.000	0.000	0.000
Ohio	2006	11,481,213	H	Legal	Legal		1	3	1	0	0.087	0.261	0.087
Ohio	2007	11,500,468	H	Legal	Legal		0	0	0	0	0.000	0.000	0.000
Ohio	2008	11,515,391	H	Legal	Legal		1	3	0	0	0.087	0.261	0.000
Ohio	2009	11,528,896	H	Legal	Legal		0	0	0	0	0.000	0.000	0.000
Ohio	2010	11,539,449	H	Legal	Legal		0	0	0	0	0.000	0.000	0.000
Ohio	2011	11,545,735	H	Legal	Legal		0	0	0	0	0.000	0.000	0.000
Ohio	2012	11,550,971	H	Legal	Legal		2	6	0	0	0.173	0.519	0.000
Ohio	2013	11,579,692	H	Legal	Legal		2	11	1	0	0.173	0.950	0.086
Ohio	2014	11,606,573	H	Legal	Legal		1	3	0	0	0.086	0.258	0.000
Ohio	2015	11,622,315	H	Legal	Legal		1	2	2	0	0.086	0.172	0.172
Ohio	2016	11,640,060	H	Legal	Legal		2	13	2	0	0.172	1.117	0.172
Ohio	2017	11,665,706	H	Legal	Legal		1	2	0	0	0.086	0.171	0.000
Ohio	2018	11,680,892	H	Legal	Legal		0	0	0	0	0.000	0.000	0.000
Ohio	2019	11,696,507	H	Legal	Legal		1	7	1	0	0.085	0.598	0.085
Ohio	2020	11,797,517	H	Legal	Legal		0	0	0	0	0.000	0.000	0.000
Oklahoma	2005	3,548,597	F	Legal	Legal		0	0	0	0	0.000	0.000	0.000
Oklahoma	2006	3,594,090	F	Legal	Legal		0	0	0	0	0.000	0.000	0.000
Oklahoma	2007	3,634,349	F	Legal	Legal		0	0	0	0	0.000	0.000	0.000
Oklahoma	2008	3,668,976	F	Legal	Legal		0	0	0	0	0.000	0.000	0.000
Oklahoma	2009	3,717,572	F	Legal	Legal		0	0	0	0	0.000	0.000	0.000
Oklahoma	2010	3,760,014	F	Legal	Legal		0	0	0	0	0.000	0.000	0.000
Oklahoma	2011	3,788,824	F	Legal	Legal		0	0	0	0	0.000	0.000	0.000
Oklahoma	2012	3,819,320	F	Legal	Legal		0	0	0	0	0.000	0.000	0.000
Oklahoma	2013	3,853,891	F	Legal	Legal		0	0	0	0	0.000	0.000	0.000
Oklahoma	2014	3,879,187	F	Legal	Legal		0	0	0	0	0.000	0.000	0.000
Oklahoma	2015	3,910,518	F	Legal	Legal		0	0	0	0	0.000	0.000	0.000
Oklahoma	2016	3,928,143	F	Legal	Legal		0	0	0	0	0.000	0.000	0.000
Oklahoma	2017	3,933,602	F	Legal	Legal		0	0	0	0	0.000	0.000	0.000

State	Year	Population	Legal Class	Def 1	Def 2	Number Licenses	Outbreaks	Illnesses	Hospitalizations	Deaths	Outbreak Rate Per 1MM	Illnesses Rate Per 1MM	Hospitalization Rate Per 1MM
Oklahoma	2018	3,943,488	F	Legal	Legal		0	0	0	0	0.000	0.000	0.000
Oklahoma	2019	3,960,676	F	Legal	Legal		0	0	0	0	0.000	0.000	0.000
Oklahoma	2020	3,964,912	F	Legal	Legal		1	8	0	0	0.252	2.018	0.000
Oregon	2005	3,613,202	R	Legal	Legal		0	0	0	0	0.000	0.000	0.000
Oregon	2006	3,670,883	R	Legal	Legal		0	0	0	0	0.000	0.000	0.000
Oregon	2007	3,722,417	R	Legal	Legal		0	0	0	0	0.000	0.000	0.000
Oregon	2008	3,768,748	R	Legal	Legal		0	0	0	0	0.000	0.000	0.000
Oregon	2009	3,808,600	R	Legal	Legal		0	0	0	0	0.000	0.000	0.000
Oregon	2010	3,837,614	R	Legal	Legal		0	0	0	0	0.000	0.000	0.000
Oregon	2011	3,872,672	R	Legal	Legal		0	0	0	0	0.000	0.000	0.000
Oregon	2012	3,900,102	R	Legal	Legal		0	0	0	0	0.000	0.000	0.000
Oregon	2013	3,924,110	R	Legal	Legal		0	0	0	0	0.000	0.000	0.000
Oregon	2014	3,965,447	R	Legal	Legal		0	0	0	0	0.000	0.000	0.000
Oregon	2015	4,018,542	R	Legal	Legal		0	0	0	0	0.000	0.000	0.000
Oregon	2016	4,093,271	R	Legal	Legal		0	0	0	0	0.000	0.000	0.000
Oregon	2017	4,147,294	R	Legal	Legal		0	0	0	0	0.000	0.000	0.000
Oregon	2018	4,183,538	R	Legal	Legal		0	0	0	0	0.000	0.000	0.000
Oregon	2019	4,216,116	R	Legal	Legal		0	0	0	0	0.000	0.000	0.000
Oregon	2020	4,244,795	R	Legal	Legal		1	4	1	0	0.236	0.942	0.236
Pennsylvania	2005	12,449,990	R	Legal	Legal		0	0	0	0	0.000	0.000	0.000
Pennsylvania	2006	12,510,809	R	Legal	Legal		0	0	0	0	0.000	0.000	0.000
Pennsylvania	2007	12,563,937	R	Legal	Legal		1	4	0	0	0.080	0.318	0.000
Pennsylvania	2008	12,612,285	R	Legal	Legal		0	0	0	0	0.000	0.000	0.000
Pennsylvania	2009	12,666,858	R	Legal	Legal		2	11	1	0	0.158	0.868	0.079
Pennsylvania	2010	12,711,406	R	Legal	Legal		2	26	4	0	0.157	2.045	0.315
Pennsylvania	2011	12,747,052	R	Legal	Legal		2	8	0	0	0.157	0.628	0.000
Pennsylvania	2012	12,769,123	R	Legal	Legal		4	163	9	0	0.313	12.765	0.705
Pennsylvania	2013	12,779,538	R	Legal	Legal		4	23	3	0	0.313	1.800	0.235
Pennsylvania	2014	12,792,392	R	Legal	Legal		2	4	0	0	0.156	0.313	0.000
Pennsylvania	2015	12,789,838	R	Legal	Legal		0	0	0	0	0.000	0.000	0.000
Pennsylvania	2016	12,788,468	R	Legal	Legal		0	0	0	0	0.000	0.000	0.000
Pennsylvania	2017	12,794,679	R	Legal	Legal		1	5	0	0	0.078	0.391	0.000
Pennsylvania	2018	12,809,107	R	Legal	Legal		3	39	3	0	0.234	3.045	0.234
Pennsylvania	2019	12,798,883	R	Legal	Legal		0	0	0	0	0.000	0.000	0.000

State	Year	Population	Legal Class	Def 1	Def 2	Number Licenses	Outbreaks	Illnesses	Hospitalizations	Deaths	Outbreak Rate Per 1MM	Illnesses Rate Per 1MM	Hospitalization Rate Per 1MM
Pennsylvania	2020	12,994,440	R	Legal	Legal		1	3	0	0	0.077	0.231	0.000
Rhode Island	2005	1,067,916	I	Illegal	Illegal		0	0	0	0	0.000	0.000	0.000
Rhode Island	2006	1,063,096	I	Illegal	Illegal		0	0	0	0	0.000	0.000	0.000
Rhode Island	2007	1,057,315	I	Illegal	Illegal		0	0	0	0	0.000	0.000	0.000
Rhode Island	2008	1,055,003	I	Illegal	Illegal		0	0	0	0	0.000	0.000	0.000
Rhode Island	2009	1,053,646	I	Illegal	Illegal		0	0	0	0	0.000	0.000	0.000
Rhode Island	2010	1,053,994	I	Illegal	Illegal		0	0	0	0	0.000	0.000	0.000
Rhode Island	2011	1,053,829	I	Illegal	Illegal		0	0	0	0	0.000	0.000	0.000
Rhode Island	2012	1,054,893	I	Illegal	Illegal		0	0	0	0	0.000	0.000	0.000
Rhode Island	2013	1,055,560	I	Illegal	Illegal		0	0	0	0	0.000	0.000	0.000
Rhode Island	2014	1,056,511	I	Illegal	Illegal		0	0	0	0	0.000	0.000	0.000
Rhode Island	2015	1,056,886	I	Illegal	Illegal		0	0	0	0	0.000	0.000	0.000
Rhode Island	2016	1,057,816	I	Illegal	Illegal		0	0	0	0	0.000	0.000	0.000
Rhode Island	2017	1,056,554	I	Illegal	Illegal		0	0	0	0	0.000	0.000	0.000
Rhode Island	2018	1,059,338	I	Illegal	Illegal		0	0	0	0	0.000	0.000	0.000
Rhode Island	2019	1,058,158	I	Illegal	Illegal		0	0	0	0	0.000	0.000	0.000
Rhode Island	2020	1,096,345	I	Illegal	Illegal		0	0	0	0	0.000	0.000	0.000
South Carolina	2005	4,270,150	F	Legal	Legal		0	0	0	0	0.000	0.000	0.000
South Carolina	2006	4,357,847	F	Legal	Legal		0	0	0	0	0.000	0.000	0.000
South Carolina	2007	4,444,110	F	Legal	Legal		1	11	4	0	0.225	2.475	0.900
South Carolina	2008	4,528,996	R	Legal	Legal		0	0	0	0	0.000	0.000	0.000
South Carolina	2009	4,589,872	R	Legal	Legal		0	0	0	0	0.000	0.000	0.000
South Carolina	2010	4,635,846	R	Legal	Legal		1	7	0	0	0.216	1.510	0.000
South Carolina	2011	4,672,655	R	Legal	Legal		2	33	3	0	0.428	7.062	0.642
South Carolina	2012	4,719,027	R	Legal	Legal		0	0	0	0	0.000	0.000	0.000
South Carolina	2013	4,766,469	R	Legal	Legal		0	0	0	0	0.000	0.000	0.000
South Carolina	2014	4,826,858	R	Legal	Legal		0	0	0	0	0.000	0.000	0.000
South Carolina	2015	4,896,006	R	Legal	Legal		0	0	0	0	0.000	0.000	0.000
South Carolina	2016	4,963,031	R	Legal	Legal		0	0	0	0	0.000	0.000	0.000
South Carolina	2017	5,027,102	R	Legal	Legal		0	0	0	0	0.000	0.000	0.000
South Carolina	2018	5,091,702	R	Legal	Legal		0	0	0	0	0.000	0.000	0.000
South Carolina	2019	5,157,702	R	Legal	Legal		0	0	0	0	0.000	0.000	0.000
South Carolina	2020	5,131,848	R	Legal	Legal		0	0	0	0	0.000	0.000	0.000
South Dakota	2005	775,493	F	Legal	Legal		0	0	0	0	0.000	0.000	0.000

State	Year	Population	Legal Class	Def 1	Def 2	Number Licenses	Outbreaks	Illnesses	Hospitalizations	Deaths	Outbreak Rate Per 1MM	Illnesses Rate Per 1MM	Hospitalization Rate Per 1MM
South Dakota	2006	783,033	F	Legal	Legal		0	0	0	0	0.000	0.000	0.000
South Dakota	2007	791,623	F	Legal	Legal		0	0	0	0	0.000	0.000	0.000
South Dakota	2008	799,124	F	Legal	Legal		0	0	0	0	0.000	0.000	0.000
South Dakota	2009	807,067	F	Legal	Legal		0	0	0	0	0.000	0.000	0.000
South Dakota	2010	816,193	F	Legal	Legal		0	0	0	0	0.000	0.000	0.000
South Dakota	2011	823,740	F	Legal	Legal		0	0	0	0	0.000	0.000	0.000
South Dakota	2012	833,859	F	Legal	Legal		0	0	0	0	0.000	0.000	0.000
South Dakota	2013	842,751	F	Legal	Legal		0	0	0	0	0.000	0.000	0.000
South Dakota	2014	849,670	F	Legal	Legal		0	0	0	0	0.000	0.000	0.000
South Dakota	2015	854,663	F	Legal	Legal		0	0	0	0	0.000	0.000	0.000
South Dakota	2016	863,693	F	Legal	Legal		0	0	0	0	0.000	0.000	0.000
South Dakota	2017	873,732	F	Legal	Legal		0	0	0	0	0.000	0.000	0.000
South Dakota	2018	879,386	F	Legal	Legal		0	0	0	0	0.000	0.000	0.000
South Dakota	2019	887,127	F	Legal	Legal		0	0	0	0	0.000	0.000	0.000
South Dakota	2020	887,799	F	Legal	Legal		0	0	0	0	0.000	0.000	0.000
Tennessee	2005	5,991,057	I	Illegal	Illegal		0	0	0	0	0.000	0.000	0.000
Tennessee	2006	6,088,766	I	Illegal	Illegal		0	0	0	0	0.000	0.000	0.000
Tennessee	2007	6,175,727	I	Illegal	Illegal		0	0	0	0	0.000	0.000	0.000
Tennessee	2008	6,247,411	I	Illegal	Illegal		0	0	0	0	0.000	0.000	0.000
Tennessee	2009	6,306,019	H	Legal	Legal		0	0	0	0	0.000	0.000	0.000
Tennessee	2010	6,355,518	H	Legal	Legal		1	3	0	0	0.157	0.472	0.000
Tennessee	2011	6,400,298	H	Legal	Legal		0	0	0	0	0.000	0.000	0.000
Tennessee	2012	6,455,752	H	Legal	Legal		0	0	0	0	0.000	0.000	0.000
Tennessee	2013	6,496,943	H	Legal	Legal		1	9	5	0	0.154	1.385	0.770
Tennessee	2014	6,544,617	H	Legal	Legal		0	0	0	0	0.000	0.000	0.000
Tennessee	2015	6,595,354	H	Legal	Legal		1	2	0	0	0.152	0.303	0.000
Tennessee	2016	6,651,277	H	Legal	Legal		0	0	0	0	0.000	0.000	0.000
Tennessee	2017	6,714,748	H	Legal	Legal		0	0	0	0	0.000	0.000	0.000
Tennessee	2018	6,778,180	H	Legal	Legal		1	17	7	0	0.148	2.508	1.033
Tennessee	2019	6,830,325	H	Legal	Legal		0	0	0	0	0.000	0.000	0.000
Tennessee	2020	6,925,619	H	Legal	Legal		0	0	0	0	0.000	0.000	0.000
Texas	2005	22,778,123	F	Legal	Legal	22	0	0	0	0	0.000	0.000	0.000
Texas	2006	23,359,580	F	Legal	Legal	13	0	0	0	0	0.000	0.000	0.000
Texas	2007	23,831,983	F	Legal	Legal	17	0	0	0	0	0.000	0.000	0.000

State	Year	Population	Legal Class	Def 1	Def 2	Number Licenses	Outbreaks	Illnesses	Hospitalizations	Deaths	Outbreak Rate Per 1MM	Illnesses Rate Per 1MM	Hospitalization Rate Per 1MM
Texas	2008	24,309,039	F	Legal	Legal	28	0	0	0	0	0.000	0.000	0.000
Texas	2009	24,801,761	F	Legal	Legal	44	0	0	0	0	0.000	0.000	0.000
Texas	2010	25,241,897	F	Legal	Legal	33	0	0	0	0	0.000	0.000	0.000
Texas	2011	25,645,504	F	Legal	Legal	42	0	0	0	0	0.000	0.000	0.000
Texas	2012	26,084,120	F	Legal	Legal	42	0	0	0	0	0.000	0.000	0.000
Texas	2013	26,479,646	F	Legal	Legal	48	0	0	0	0	0.000	0.000	0.000
Texas	2014	26,963,092	F	Legal	Legal	52	0	0	0	0	0.000	0.000	0.000
Texas	2015	27,468,531	F	Legal	Legal	44	2	7	1	0	0.073	0.255	0.036
Texas	2016	27,914,064	F	Legal	Legal	44	0	0	0	0	0.000	0.000	0.000
Texas	2017	28,291,024	F	Legal	Legal	0	0	0	0	0	0.000	0.000	0.000
Texas	2018	28,624,564	F	Legal	Legal	0	1	2	0	0	0.035	0.070	0.000
Texas	2019	28,986,794	F	Legal	Legal	0	1	6	0	0	0.034	0.207	0.000
Texas	2020	29,232,474	F	Legal	Legal	0	0	0	0	0	0.000	0.000	0.000
Utah	2005	2,457,719	F	Legal	Legal	2	1	11	0	0	0.407	4.476	0.000
Utah	2006	2,525,507	F	Legal	Legal	2	0	0	0	0	0.000	0.000	0.000
Utah	2007	2,597,746	R	Legal	Legal	2	0	0	0	0	0.000	0.000	0.000
Utah	2008	2,663,029	R	Legal	Legal	2	1	4	0	0	0.376	1.502	0.000
Utah	2009	2,723,421	R	Legal	Legal	2	0	0	0	0	0.000	0.000	0.000
Utah	2010	2,775,413	R	Legal	Legal	2	0	0	0	0	0.000	0.000	0.000
Utah	2011	2,814,797	R	Legal	Legal	3	0	0	0	0	0.000	0.000	0.000
Utah	2012	2,854,146	R	Legal	Legal	4	2	20	0	0	0.701	7.007	0.000
Utah	2013	2,898,773	R	Legal	Legal	4	2	28	1	0	0.690	9.659	0.345
Utah	2014	2,938,327	R	Legal	Legal	6	2	118	10	1	0.681	40.159	3.403
Utah	2015	2,983,626	R	Legal	Legal	8	2	6	0	0	0.670	2.011	0.000
Utah	2016	3,044,241	R	Legal	Legal	10	2	25	5	0	0.657	8.212	1.642
Utah	2017	3,103,540	R	Legal	Legal	8	4	21	1	0	1.289	6.766	0.322
Utah	2018	3,155,153	R	Legal	Legal	9	1	16	0	0	0.317	5.071	0.000
Utah	2019	3,203,383	R	Legal	Legal	9	0	0	0	0	0.000	0.000	0.000
Utah	2020	3,283,785	R	Legal	Legal	10	0	0	0	0	0.000	0.000	0.000
Vermont	2005	621,215	F	Legal	Legal		0	0	0	0	0.000	0.000	0.000
Vermont	2006	622,892	F	Legal	Legal		0	0	0	0	0.000	0.000	0.000
Vermont	2007	623,481	F	Legal	Legal		0	0	0	0	0.000	0.000	0.000
Vermont	2008	624,151	F	Legal	Legal		1	6	3	0	1.602	9.613	4.807
Vermont	2009	624,817	F	Legal	Legal		0	0	0	0	0.000	0.000	0.000

State	Year	Population	Legal Class	Def 1	Def 2	Number Licenses	Outbreaks	Illnesses	Hospitalizations	Deaths	Outbreak Rate Per 1MM	Illnesses Rate Per 1MM	Hospitalization Rate Per 1MM
Vermont	2010	625,886	F	Legal	Legal		2	17	0	0	3.195	27.161	0.000
Vermont	2011	627,197	F	Legal	Legal		0	0	0	0	0.000	0.000	0.000
Vermont	2012	626,361	F	Legal	Legal		0	0	0	0	0.000	0.000	0.000
Vermont	2013	626,603	F	Legal	Legal		0	0	0	0	0.000	0.000	0.000
Vermont	2014	625,693	F	Legal	Legal		0	0	0	0	0.000	0.000	0.000
Vermont	2015	625,810	F	Legal	Legal		0	0	0	0	0.000	0.000	0.000
Vermont	2016	624,366	F	Legal	Legal		0	0	0	0	0.000	0.000	0.000
Vermont	2017	625,132	F	Legal	Legal		0	0	0	0	0.000	0.000	0.000
Vermont	2018	624,802	F	Legal	Legal		0	0	0	0	0.000	0.000	0.000
Vermont	2019	624,046	F	Legal	Legal		0	0	0	0	0.000	0.000	0.000
Vermont	2020	642,893	F	Legal	Legal		0	0	0	0	0.000	0.000	0.000
Virginia	2005	7,577,105	H	Legal	Legal		0	0	0	0	0.000	0.000	0.000
Virginia	2006	7,673,725	H	Legal	Legal		1	9	0	0	0.130	1.173	0.000
Virginia	2007	7,751,000	H	Legal	Legal		0	0	0	0	0.000	0.000	0.000
Virginia	2008	7,833,496	H	Legal	Legal		0	0	0	0	0.000	0.000	0.000
Virginia	2009	7,925,937	H	Legal	Legal		0	0	0	0	0.000	0.000	0.000
Virginia	2010	8,024,004	H	Legal	Legal		0	0	0	0	0.000	0.000	0.000
Virginia	2011	8,102,437	H	Legal	Legal		0	0	0	0	0.000	0.000	0.000
Virginia	2012	8,187,456	H	Legal	Legal		0	0	0	0	0.000	0.000	0.000
Virginia	2013	8,255,861	H	Legal	Legal		0	0	0	0	0.000	0.000	0.000
Virginia	2014	8,315,430	H	Legal	Legal		0	0	0	0	0.000	0.000	0.000
Virginia	2015	8,367,303	H	Legal	Legal		0	0	0	0	0.000	0.000	0.000
Virginia	2016	8,417,651	H	Legal	Legal		1	14	7	0	0.119	1.663	0.832
Virginia	2017	8,471,011	H	Legal	Legal		0	0	0	0	0.000	0.000	0.000
Virginia	2018	8,510,920	H	Legal	Legal		0	0	0	0	0.000	0.000	0.000
Virginia	2019	8,556,642	H	Legal	Legal		0	0	0	0	0.000	0.000	0.000
Virginia	2020	8,636,471	H	Legal	Legal		0	0	0	0	0.000	0.000	0.000
Washington	2005	6,257,305	R	Legal	Legal		0	0	0	0	0.000	0.000	0.000
Washington	2006	6,370,753	R	Legal	Legal		1	2	1	0	0.157	0.314	0.157
Washington	2007	6,461,587	R	Legal	Legal		1	18	0	0	0.155	2.786	0.000
Washington	2008	6,562,231	R	Legal	Legal		0	0	0	0	0.000	0.000	0.000
Washington	2009	6,667,426	R	Legal	Legal		1	3	0	0	0.150	0.450	0.000
Washington	2010	6,743,009	R	Legal	Legal		2	8	1	0	0.297	1.186	0.148
Washington	2011	6,827,479	R	Legal	Legal		0	0	0	0	0.000	0.000	0.000

State	Year	Population	Legal Class	Def 1	Def 2	Number Licenses	Outbreaks	Illnesses	Hospitalizations	Deaths	Outbreak Rate Per 1MM	Illnesses Rate Per 1MM	Hospitalization Rate Per 1MM
Washington	2012	6,898,599	R	Legal	Legal		0	0	0	0	0.000	0.000	0.000
Washington	2013	6,966,252	R	Legal	Legal		0	0	0	0	0.000	0.000	0.000
Washington	2014	7,057,531	R	Legal	Legal		0	0	0	0	0.000	0.000	0.000
Washington	2015	7,167,287	R	Legal	Legal		0	0	0	0	0.000	0.000	0.000
Washington	2016	7,299,961	R	Legal	Legal		1	3	3	0	0.137	0.411	0.411
Washington	2017	7,427,951	R	Legal	Legal		0	0	0	0	0.000	0.000	0.000
Washington	2018	7,526,793	R	Legal	Legal		1	2	0	0	0.133	0.266	0.000
Washington	2019	7,614,024	R	Legal	Legal		1	2	0	0	0.131	0.263	0.000
Washington	2020	7,724,031	R	Legal	Legal		0	0	0	0	0.000	0.000	0.000
West Virginia	2005	1,820,492	I	Illegal	Illegal		0	0	0	0	0.000	0.000	0.000
West Virginia	2006	1,827,912	I	Illegal	Illegal		0	0	0	0	0.000	0.000	0.000
West Virginia	2007	1,834,052	I	Illegal	Illegal		0	0	0	0	0.000	0.000	0.000
West Virginia	2008	1,840,310	I	Illegal	Illegal		0	0	0	0	0.000	0.000	0.000
West Virginia	2009	1,847,775	I	Illegal	Illegal		0	0	0	0	0.000	0.000	0.000
West Virginia	2010	1,854,265	I	Illegal	Illegal		0	0	0	0	0.000	0.000	0.000
West Virginia	2011	1,856,606	I	Illegal	Illegal		0	0	0	0	0.000	0.000	0.000
West Virginia	2012	1,857,446	I	Illegal	Illegal		0	0	0	0	0.000	0.000	0.000
West Virginia	2013	1,854,768	I	Illegal	Illegal		0	0	0	0	0.000	0.000	0.000
West Virginia	2014	1,850,569	I	Illegal	Illegal		0	0	0	0	0.000	0.000	0.000
West Virginia	2015	1,843,332	I	Illegal	Illegal		0	0	0	0	0.000	0.000	0.000
West Virginia	2016	1,832,435	H	Legal	Legal		0	0	0	0	0.000	0.000	0.000
West Virginia	2017	1,818,683	H	Legal	Legal		0	0	0	0	0.000	0.000	0.000
West Virginia	2018	1,805,953	H	Legal	Legal		0	0	0	0	0.000	0.000	0.000
West Virginia	2019	1,795,263	H	Legal	Legal		0	0	0	0	0.000	0.000	0.000
West Virginia	2020	1,791,420	H	Legal	Legal		0	0	0	0	0.000	0.000	0.000
Wisconsin	2005	5,546,166	F	Legal	Legal		0	0	0	0	0.000	0.000	0.000
Wisconsin	2006	5,577,655	F	Legal	Legal		0	0	0	0	0.000	0.000	0.000
Wisconsin	2007	5,610,775	F	Legal	Legal		0	0	0	0	0.000	0.000	0.000
Wisconsin	2008	5,640,996	F	Legal	Legal		0	0	0	0	0.000	0.000	0.000
Wisconsin	2009	5,669,264	F	Legal	Legal		1	52	1	0	0.176	9.172	0.176
Wisconsin	2010	5,690,538	F	Legal	Legal		0	0	0	0	0.000	0.000	0.000
Wisconsin	2011	5,705,840	F	Legal	Legal		1	16	1	0	0.175	2.804	0.175
Wisconsin	2012	5,720,825	F	Legal	Legal		0	0	0	0	0.000	0.000	0.000
Wisconsin	2013	5,738,012	F	Legal	Legal		1	3	1	0	0.174	0.523	0.174

State	Year	Population	Legal Class	Def 1	Def 2	Number Licenses	Outbreaks	Illnesses	Hospitalizations	Deaths	Outbreak Rate Per 1MM	Illnesses Rate Per 1MM	Hospitalization Rate Per 1MM
Wisconsin	2014	5,753,199	F	Legal	Legal		1	38	10	0	0.174	6.605	1.738
Wisconsin	2015	5,762,927	F	Legal	Legal		0	0	0	0	0.000	0.000	0.000
Wisconsin	2016	5,775,170	F	Legal	Legal		0	0	0	0	0.000	0.000	0.000
Wisconsin	2017	5,793,147	F	Legal	Legal		0	0	0	0	0.000	0.000	0.000
Wisconsin	2018	5,809,319	F	Legal	Legal		0	0	0	0	0.000	0.000	0.000
Wisconsin	2019	5,824,581	F	Legal	Legal		0	0	0	0	0.000	0.000	0.000
Wisconsin	2020	5,896,271	F	Legal	Legal		0	0	0	0	0.000	0.000	0.000
Wyoming	2005	514,157	I	Illegal	Illegal		2	14	2	0	3.890	27.229	3.890
Wyoming	2006	522,667	I	Illegal	Illegal		0	0	0	0	0.000	0.000	0.000
Wyoming	2007	534,876	I	Illegal	Illegal		0	0	0	0	0.000	0.000	0.000
Wyoming	2008	546,043	I	Illegal	Illegal		0	0	0	0	0.000	0.000	0.000
Wyoming	2009	559,851	I	Illegal	Illegal		0	0	0	0	0.000	0.000	0.000
Wyoming	2010	564,531	I	Illegal	Illegal		0	0	0	0	0.000	0.000	0.000
Wyoming	2011	567,491	I	Illegal	Illegal		0	0	0	0	0.000	0.000	0.000
Wyoming	2012	576,656	H	Legal	Legal		0	0	0	0	0.000	0.000	0.000
Wyoming	2013	582,620	H	Legal	Legal		0	0	0	0	0.000	0.000	0.000
Wyoming	2014	583,159	H	Legal	Legal		0	0	0	0	0.000	0.000	0.000
Wyoming	2015	586,389	F	Legal	Legal		0	0	0	0	0.000	0.000	0.000
Wyoming	2016	585,243	F	Legal	Legal		0	0	0	0	0.000	0.000	0.000
Wyoming	2017	579,994	F	Legal	Legal		0	0	0	0	0.000	0.000	0.000
Wyoming	2018	579,054	F	Legal	Legal		0	0	0	0	0.000	0.000	0.000
Wyoming	2019	580,116	F	Legal	Legal		0	0	0	0	0.000	0.000	0.000
Wyoming	2020	577,605	F	Legal	Legal		0	0	0	0	0.000	0.000	0.000

Table S2. Data table of pasteurized milk-related outbreaks listing illnesses, hospitalizations, and deaths in the United States for the years 2005-2020.

CDCID Outbreak	State	Year	Illnesses	Hospitalizations	Deaths	Agent/Pathogen Genus	Pathogen species
256570	Colorado	2005	200	1	0	Campylobacter	jejuni
261955	California	2006	1644	7	0	Campylobacter	jejuni
258315	Connecticut	2007	11	0	0	Norovirus	Genogroup II
259704	Pennsylvania	2007	3	0	0	Campylobacter	unknown
260049	Massachusetts	2007	5	5	3	Listeria	monocytogenes
4345	Wyoming	2009	22	0	0	Norovirus	Genogroup II
3822	Colorado	2010	3	0	0	Campylobacter	jejuni
5290	Florida	2010	4	0	0	Salmonella	unknown
8270	Pennsylvania	2011	16	7	1	Yersinia	enterocolitica
9044	Washington	2011	3	3	0	Escherichia	coli, Shiga toxin-producing
9805	Kansas	2012	18	0	0	Campylobacter	jejuni
14657	Maryland	2012	6	0	0	Other - Chemical/Toxin	
15143	Alaska	2013	5	1	0	Campylobacter	jejuni
18884	Florida	2014	6	0	0	Cleaning agents	
19133	Virginia	2014	32	0	0	Other - Bacterium	
280366	Florida	2018	4	0	0	Unknown	
282360	Nebraska	2018	20	1	0	Salmonella	enterica
289346	Pennsylvania	2019	109	7	0	Yersinia	enterocolitica

Table S3. Raw milk legal classifications for all states by state-year according to the Table 1 definitions: R (Legal off-farm sales in retail stores, at farm markets, or both), F (Farm-gate sales legal, but no off-farm sales), H (Herdshares permitted by law or policy or no law prohibits herdshares), P (Farm-gate sales legal with "pet food" license), and I (Both herdshares and other sales illegal.).

State	2004	2005	2006	2007	2008	2009	2010	2011	2012	2013	2014	2015	2016	2017	2018	2019	2020
Alabama	P	P	P	P	P	P	P	P	P	P	P	P	P	P	P	P	P
Alaska	H	H	H	H	H	H	H	H	H	H	H	H	H	H	H	H	H
Arizona	R	R	R	R	R	R	R	R	R	R	R	R	R	R	R	R	R
Arkansas	F	F	F	F	F	F	F	F	F	F	F	F	F	F	F	F	F
California	R	R	R	R	R	R	R	R	R	R	R	R	R	R	R	R	R
Colorado	I	H	H	H	H	H	H	H	H	H	H	H	H	H	H	H	H
Connecticut	R	R	R	R	R	R	R	R	R	R	R	R	R	R	R	R	R
Delaware	I	I	I	I	I	I	I	I	I	I	I	I	I	I	I	I	I
District of Columbia	I	I	I	I	I	I	I	I	I	I	I	I	I	I	I	I	I
Florida	P	P	P	P	P	P	P	P	P	P	P	P	P	P	P	P	P
Georgia	P	P	P	P	P	P	P	P	P	P	P	P	P	P	P	P	P
Hawaii	I	I	I	I	I	I	I	I	I	I	I	I	I	I	I	I	I
Idaho	R	R	R	R	R	R	R	R	R	R	R	R	R	R	R	R	R
Illinois	H	H	H	H	H	H	H	H	H	H	H	H	F	F	F	F	F
Indiana	H	H	H	H	H	H	H	H	H	H	H	H	H	H	H	H	H
Iowa	I	I	I	I	I	I	I	I	I	I	I	I	I	I	I	I	I
Kansas	F	F	F	F	F	F	F	F	F	F	F	F	F	F	F	F	F
Kentucky	I	I	I	I	I	I	I	H	H	H	H	H	H	H	H	H	H
Louisiana	I	I	I	I	I	I	I	I	I	I	I	I	I	I	I	I	I
Maine	R	R	R	R	R	R	R	R	R	R	R	R	R	R	R	R	R
Maryland	I	I	I	I	I	I	I	I	I	I	I	I	P	P	P	P	P
Massachusetts	F	F	F	F	F	F	F	F	F	F	F	F	F	F	F	F	F
Michigan	I	I	I	I	I	I	I	I	I	H	H	H	H	H	H	H	H
Minnesota	F	F	F	F	F	F	F	F	F	F	F	F	F	F	F	F	F
Mississippi	F	F	F	F	F	F	F	F	F	F	F	F	F	F	F	F	F
Missouri	F	F	F	F	F	F	F	F	F	F	F	F	F	F	F	F	F
Montana	I	I	I	I	I	I	I	I	I	I	I	I	I	H	H	H	H
Nebraska	F	F	F	F	F	F	F	F	F	F	F	F	F	F	F	F	F
Nevada	I	I	I	I	I	I	I	I	I	I	I	I	I	I	I	I	I
New Hampshire	R	R	R	R	R	R	R	R	R	R	R	R	R	R	R	R	R
New Jersey	I	I	I	I	I	I	I	I	I	I	I	I	I	I	I	I	I
New Mexico	R	R	R	R	R	R	R	R	R	R	R	R	R	R	R	R	R

State	2004	2005	2006	2007	2008	2009	2010	2011	2012	2013	2014	2015	2016	2017	2018	2019	2020
New York	F	F	F	F	F	F	F	F	F	F	F	F	F	F	F	F	F
North Carolina	P	P	P	P	P	P	P	P	P	P	P	P	P	P	H	H	H
North Dakota	I	I	I	I	I	I	I	I	I	H	H	H	H	H	H	H	H
Ohio	I	I	H	H	H	H	H	H	H	H	H	H	H	H	H	H	H
Oklahoma	F	F	F	F	F	F	F	F	F	F	F	F	F	F	F	F	F
Oregon	R	R	R	R	R	R	R	R	R	R	R	R	R	R	R	R	R
Pennsylvania	R	R	R	R	R	R	R	R	R	R	R	R	R	R	R	R	R
Rhode Island	I	I	I	I	I	I	I	I	I	I	I	I	I	I	I	I	I
South Carolina	F	F	F	F	R	R	R	R	R	R	R	R	R	R	R	R	R
South Dakota	F	F	F	F	F	F	F	F	F	F	F	F	F	F	F	F	F
Tennessee	I	I	I	I	I	H	H	H	H	H	H	H	H	H	H	H	H
Texas	F	F	F	F	F	F	F	F	F	F	F	F	F	F	F	F	F
Utah	F	F	F	R	R	R	R	R	R	R	R	R	R	R	R	R	R
Vermont	F	F	F	F	F	F	F	F	F	F	F	F	F	F	F	F	F
Virginia	H	H	H	H	H	H	H	H	H	H	H	H	H	H	H	H	H
Washington	R	R	R	R	R	R	R	R	R	R	R	R	R	R	R	R	R
West Virginia	I	I	I	I	I	I	I	I	I	I	I	I	H	H	H	H	H
Wisconsin	I	F	F	F	F	F	F	F	F	F	F	F	F	F	F	F	F
Wyoming	I	I	I	I	I	I	I	I	H	H	H	F	F	F	F	F	F

Table S4. State Level Data on Legal Status with Production and Population Estimates for 2018.

State	Year of Legal Status Change	Legal Status Code	**Text description of legal access from RealMilk legal map** (11 Nov 2022 version) with updates by personal communication	Number Milk Cows 2018 (thousands)	2018 State Milk Production (million pounds)	2018 State Human Population
Alabama	(P since 2004)	P	Sales of raw pet milk are legal.	5	73	4,891,628
Alaska	Since 2004	H	Distribution of raw milk through herdshares is legal by regulation. State interprets regulation to mean no other raw dairy products can be distributed through herdshare agreements; 2021 raw dairy products through herdshare	<1	3	736,624
Arizona	Since 2004	R	Retail sales of raw milk are legal as well as sales of cream butter yogurt and kefir; a license is required. The state department of agriculture takes the position that herdshares are illegal.	208	4978	7,164,228
Arkansas	Since 2004	F	On-farm sales of raw cow, goat, or sheep milk are legal; no license is required. Producers can sell up to 500 gallons per month.	6	74	3,012,161
California	Since 2004	R	Retail sales of raw milk are legal as well as sales of raw butter, cream, and kefir; a license is required.	1,734	40,413	39,437,463
Colorado	Since 2005	H	Distribution of raw milk through herdshares is legal; producers must register with the state. Delivery from the farm is allowed by other herdshare co-owners.	176	4,557	5,697,155
Connecticut	Since 2004	H, R	Retail sales of raw milk are legal; registration with the commissioner of agriculture is required. Herdshares are legal by statute.	19	427	3,574,561
Delaware	P since 2022;<br>I prior	P	The sale or distribution of raw milk for human consumption is illegal. Sales of raw pet milk are legal if the producer is registered with the Delaware Department of Agriculture.	5	92	966,985
District of Columbia	I since 2004	PNF	The sale or distribution of raw milk for human consumption is illegal. National manufacturers sell raw pet dairy in retail stores.	Not Reported	Not Reported	704,147
Florida	P since 2004	P	Sales of raw pet milk are legal; a Master Feed Registration is required, and sales of other raw pet dairy products by producers and distributors are allowed.	120	2,381	21,254,926

State	Year of Legal Status Change	Legal Status Code	**Text description of legal access from RealMilk legal map** (11 Nov 2022 version) with updates by personal communication	**Number Milk Cows 2018** (thousands)	**2018 State Milk Production** (million pounds)	**2018 State Human Population**
Georgia	P since 2004;<br>F as of July 2023	P, F	Sales of raw pet milk are legal. A commercial feed permit is required; no other raw dairy products can be sold under permit. As of 1 July 2023, on-farm access and delivery of raw milk are legal.[d]	83	1,766	10,519,389
Hawaii	I since 2004	NLH	Raw milk sales for human consumption are illegal. There's no law on herdshares but the state claims they are illegal. Currently, no farmers are selling raw pet milk, and national manufacturers are no longer selling raw pet milk in retail stores. The statute on raw pet milk is unclear; however, the health department raided stores, seized products and banned raw pet milk sales in 2021.	2	34	1,423,102
Idaho	Since 2004	H, F, R	Retail sales of raw milk are legal as well as sales of raw butter, yogurt, kefir, and unaged cheese; a permit is required. Herdshares are legal if the producer is registered and if the herd is no larger than 7 cows, 15 goats, or 15 sheep. Non-retail (on-farm) sales are legal if the producer is registered with the state and has no more than 3 cows, 7 goats, or 7 sheep.	609	15,149	1,752,074
Illinois	2016 (H prior)	F	On-farm sales of raw milk are legal; two permits are required: a dairy farm permit and a distribution point permit.	90	1,878	12,724,685
Indiana	H since 2004	NLH, P	There's no law on herdshares; the state is aware that herdshares exist but has taken no action to stop them. Sales of raw pet dairy are legal.	83	4,161	6,698,481
Iowa	I since 2004	NLH, F, PNF	Raw milk sales for human consumption are illegal. There's no law on herdshares but the state Department of Agriculture and Land Stewardship claims they are illegal. Currently, no farmers are selling raw pet milk, but national manufacturers are selling raw pet dairy in retail stores. As of 1 July 2023, legal access to raw milk and raw milk products is permitted on-farm for operations with 10 or fewer milk-producing cows. [d]	220	5,268	3,149,900
Kansas	Since 2004	F	On-farm raw milk sales are legal as well as sales of raw butter, cream, yogurt, kefir, and unaged cheese; no permit is required.	159	3,708	2,912,748

State	Year of Legal Status Change	Legal Status Code	**Text description of legal access from RealMilk legal map** (11 Nov 2022 version) with updates by personal communication	**Number Milk Cows 2018** (thousands)	**2018 State Milk Production** (million pounds)	**2018 State Human Population**
Kentucky	H 2011 (I prior)	NLH	There's no law on herdshares; the state is aware herdshares exist and has taken no action to stop them. Sales of raw goat milk are legal if the farmer has permit and if the consumer has written recommendation from physician.	55	1,009	4,464,273
Louisiana	I since 2004	PNF	Raw milk sales for human consumption are illegal. Currently, no farmers are selling pet milk, but national manufacturers are selling raw pet dairy in retail stores.	11	152	4,664,450
Maine	Since 2004	R	Retail sales of raw milk are legal as well as sales of raw butter, cream, yogurt, and kefir; a permit is required. 2017 Unlicensed, unregulated direct-to-consumer sales of all raw dairy are legal in towns that have passed the local food sovereignty ordinance.	30	618	1,340,123
Maryland	P 2016 (I prior)	P	Raw milk sales are illegal by statute, and herdshare distribution is prohibited by regulation. Sales of raw pet milk are legal if the producer is registered with the state.	45	925	6,042,153
Massachusetts	F since 2004?	F	On-farm sales of raw milk are legal; a certificate of registration is required.	11	202	6,885,720
Michigan	H 2013; I prior	H	Herdshares are legal by written policy 1.40 which says that only raw milk can be distributed and that there is to be no enforcement of pasteurization requirements against herdshare operations distributing raw milk. There is a court case, however, where the court ruled that the farmer distributing raw cream and butter to any shareowners was not in contempt of court as violating state dairy laws.	423	11,168	9,987,286
Minnesota	Since 2004	F	On farm sales of raw milk are legal as well as sales of raw cream; no permit is required.274 The law states that the pasteurization requirement doesn't "apply to milk, cream, or skim milk occasionally secured or purchased for personal use by a consumer at the place or farm" where the milk is produced.	452	9,868	5,608,762
Mississippi	F Since 2004!	F_g	On-farm sales of goat milk legal; the producer may have only nine lactating goats at one time.	9	129	2,982,879
Missouri	Since 2004	F, FM	On-farm sales of raw milk are legal as well as sales of raw cream; producers without a permit may sell through delivery. Producers with a permit may	83	1,194	6,125,986

State	Year of Legal Status Change	Legal Status Code	**Text description of legal access from RealMilk legal map** (11 Nov 2022 version) with updates by personal communication	Number Milk Cows 2018 (thousands)	2018 State Milk Production (million pounds)	2018 State Human Population
			additionally sell at farmers market and other direct-to-consumer venues, but no sales at retail stores.			
Montana	H 2017;<br>I prior	H	Sales of raw milk are legal to consumers who have purchased stock in the producer's dairy animals; sales are legal under the state's exemption to securities registration laws. Producers can sell securities to individual investors or to consumer cooperatives.<br>2021 raw milk and raw milk products for producers with <=5 lactating cows, <=10 lactating goats/sheep	12	274	1,061,818
Nebraska	Since 2004	F	On-farm sales of raw milk are legal as well as sales of raw cream; no permit is required.	60	1,440	1,925,512
Nevada	I since 2004	I	Raw milk sales are de facto illegal; sales are legal by statute but only if the county milk commission approves the producer. Only one county (Nye) has a milk commission and that county has not approved anyone. Raw pet milk sales are legal, but state law requires a toxic dye to denature the milk.	32	734	3,030,725
New Hampshire	Since 2004	F, FM, R	Retail sales of raw milk are legal as well as sales of raw butter, cream, yogurt, and kefir; a permit is required, but it is left to the county or municipality to allow retail sales and sales to restaurants. Unregulated producers can sell direct to consumers on-farm, farm stands, or at farmers markets; they may sell up to 20 gallons of raw milk a day or the equivalent processed into raw butter, cream, yogurt, or kefir as well as cheese aged 60 days or more.<br>2022 legalized unregulated sale raw ice cream; producers must meet labeling requirements and cap on daily sales	12	249	1,355,064
New Jersey	P 2021;<br>I prior	P	Sales of raw pet milk are legal if the producer is registered with the state department of agriculture; raw milk distribution or sale for human consumption is illegal.	6	110	8,891,730
New Mexico	Since 2004	R	Retail sales of raw milk are legal as well as sales of raw cream; a permit is required. Municipalities can ban sales, which Albuquerque has done. There is no law on herdshares	330	8,285	2,093,754
New York	Since 2004	F	On-farm sales of raw milk are legal; a permit is required.	623	14,882	19,544,098

State	Year of Legal Status Change	Legal Status Code	**Text description of legal access from RealMilk legal map** (11 Nov 2022 version) with updates by personal communication	Number Milk Cows 2018 (thousands)	**2018 State Milk Production** (million pounds)	2018 State Human Population
North Carolina	P since 2004; H since 2018	H, P	Distribution of any raw dairy product through herdshares is legal. Raw pet milk sales without a permit are legal; producers of pet milk must submit labels to the Department of Environment and Natural Resources.	44	937	10,391,358
North Dakota	H 2013; I prior	H	Distribution of raw milk through herdshares is legal. Statute also allows for distribution of other raw dairy products, but the state department of agriculture contests this, claiming shareowners can only obtain raw milk. As of 1 July 2023, shareowners can obtain any raw milk product.[d]	15	334	760,062
Ohio	H since 2006; I prior	H	Distribution of raw milk and raw milk products through herdshares is legal by court decision in Darke County and by policy for the rest of the state.	259	5,532	11,680,892
Oklahoma	Since 2004	F	On-farm sales of raw milk are legal; statute allows 'incidental sales'—the term is not defined—for raw cow milk or goat milk sales of no more than 100 gallons per month.	40	725	3,943,488
Oregon	R since 2004	F, R_g	Retail sales of raw goat milk and sheep milk are legal as well as sales of raw cream; a permit is required. Unlicensed on-farm sales of raw cow, goat, and sheep milk are allowed if there are no more than three lactating cows, nine goats, and nine sheep on the premises.	123	2,531	4,183,538
Pennsylvania	Since 2004	F, R	Retail sales of raw milk are legal as well as sales in restaurants; a permit is required. Producers selling in retail stores must have mechanical bottling–no handcapping allowed. Consumers can bring their own containers for on-farm purchases.	519	10,665	12,809,107
Rhode Island	I since 2004	PNF	Raw milk sales are illegal. There is an exception for consumers purchasing raw goat milk with a physician's prescription. Currently, no farmers are selling raw pet milk, but national manufacturers are selling raw pet dairy in retail stores.	<1	12	1,059,338
South Carolina	R since 2008; F2004-2007	R	Retail sales of raw milk are legal; a permit is required. The state issued proposed regulations legalizing the sale of raw cream and raw buttermilk in 2020.	14	242	5,091,702

State	Year of Legal Status Change	Legal Status Code	**Text description of legal access from RealMilk legal map** (11 Nov 2022 version) with updates by personal communication	**Number Milk Cows 2018** (thousands)	**2018 State Milk Production** (million pounds)	**2018 State Human Population**
South Dakota	Since 2004	F	On-farm sales of raw milk are legal as well as sales of raw cream; a permit is required. Delivery is also legal.	121	2,705	879,386
Tennessee	H since 2009;<br>I prior	H	Distribution of any raw dairy product through herdshares is legal. Sales of raw butter are legal by producers who have a dairy plant permit. Sales of raw pet dairy products are legal if the producer has a commercial feed permit.<br>2019 retail sale raw butter by licensed producers	37	634	6,778,180
Texas	Since 2004	F	On-farm sales of raw milk are legal as well as sales of raw cream; a permit is required. State policy allows an agent for the consumer to deliver raw milk.<br>2021 delivery by licensed producers, raw milk products (buttermilk, cream, kefir)	537	12,852	28,624,564
Utah	R since 2007;<br>F prior	H, FM, R	Retail sales of raw milk are legal if the licensed producer has a majority ownership in the retail store. Licensees may also sell raw milk, butter and cream on-farm as well as deliver and/or sell via refrigerated mobile unit. Unlicensed producers can sell up to 120 gallons of raw milk per month on the farm. Herdshares are legal by statute; producers with no more than 2 cows, 10 goats, and 10 sheep can distribute raw milk and any other raw dairy product to their shareowners. As of 1 July 2023, all raw dairy products can be sold at retail. [d]	100	2,322	3,155,153
Vermont	F since 2004;	F, FM	On-farm sales of raw milk are legal; producers meeting basic requirements can sell up to 87.5 gallons a week without a permit. Producers meeting additional requirements can sell up to 350 gallons per week and can deliver as well as sell at farmers markets.<br><br>2019 raw milk delivery and farmers markets;<br>2021 raw milk by CSAs and 3rd party farm stands	127	2,680	624,802
Virginia	H since 2004	HNL	There's no law on herdshares; distribution of raw milk and raw products through herdshares is legal by policy of the Virginia Department of Agriculture and Consumer Services.	83	1,635	8,510,920
Washington	Since 2004	R	Retail sales of raw milk are legal as well as sales of raw cream; a license is required, and sales to restaurants are also legal.	277	6,736	7,526,793

State	Year of Legal Status Change	Legal Status Code	**Text description of legal access from RealMilk legal map** (11 Nov 2022 version) with updates by personal communication	**Number Milk Cows 2018** (thousands)	**2018 State Milk Production** (million pounds)	**2018 State Human Population**
West Virginia	H since 2016;<br>I prior	H	Distribution of raw milk through herdshares is legal; herdshare producers must register with the state.	7	111	1,805,953
Wisconsin	F since 2005;<br>I prior	F	On-farm sales of raw milk are legal as ’incidental sales’, which is defined as ”not in the regular course of business” (the state has not clarified what the ”regular course of business” is).Raw milk sales also legal to those individuals who have a bona fide ownership interest in the milk producer license; farmers with a milk producer license can issue stock to those wanting milk as long as the sole purpose of the business isn’t to sell raw milk and the investors have the risk of loss for their investment.	1,274	30,579	5,809,319
Wyoming	F since 2015;<br>H 2012-2014;<br>I prior	H, F, FM, R	On-farm sales of raw milk direct to the consumer are legal under the Wyoming Food Freedom Act. These unregulated sales can be on-farm, through delivery, and at farmers markets. Distribution of raw milk through herdshares is legal by regulation. As of 1 July 2023, any raw milk product can be told at retail.[d]	6	142	579,054
**US TOTALS**	**9,398**	**217,575**	**326,838,199**			

Codes for legal status: **R**=retail; **F**=farm; **FM**=farmers market or other direct-to-consumer venues; **H**=herdshare legal; **NLH**=no law prohibiting herdshare; **HI**=herdshare illegal; **P**=pet food legal; **PNF**=pet food legal, no farmers selling; **I**=all sales illegal.

Notes on data sources: Data on legal status from Whitehead and Lake (2018), Supplementary Table 1 on legal status; 10.1371/currents.outbreaks.bae5a0fd685616839c9cf857792730d1; updates on state laws by Peter Kennedy (2023), personal communications, 15 Feb 2023, 27 June 2023; classifications on legal status by Weston A Price Foundation Real Milk Legal Map. Last updated on 11 November 2022. https://www.realmilk.com/real-milk-legal-map/; Census of Agriculture (2021) documents numbers of cows and production of fluid milk including milk consumed on farm by state (Table 8.3); US Census (2021) human populations by state

1.5 Autocorrelation Function Plots for Time-Series Counts

Autocorrelation function (ACF) plots were generated in Minitab v21.4 using the autocorrelation module (Minitab, 2022). The plots show the lags on the x-axis, which range from 1 to 4, and the ACF on the y-axis. The red horizontal lines show the 95% significance limits for autocorrelation. ACF plots were not generated for 14 states that reported no raw milk outbreaks over the entire period (AL, AR, DC, DE, HI, LA, MD, MS, NE, NJ, NV, RI, SD, and WV) as the time-series records were all zeros. Example plots are provided below. Additional plots will be provided upon request.

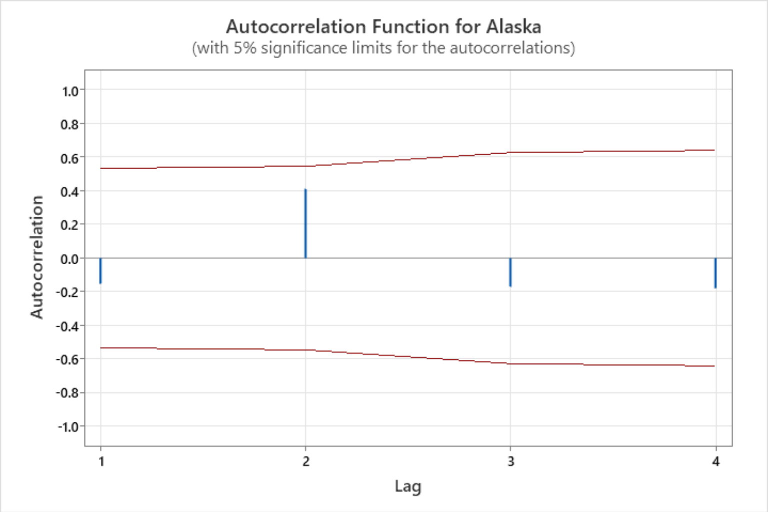

Fig. S7. Autocorrelation function (ACF) plot of raw milk outbreaks for Alaska.

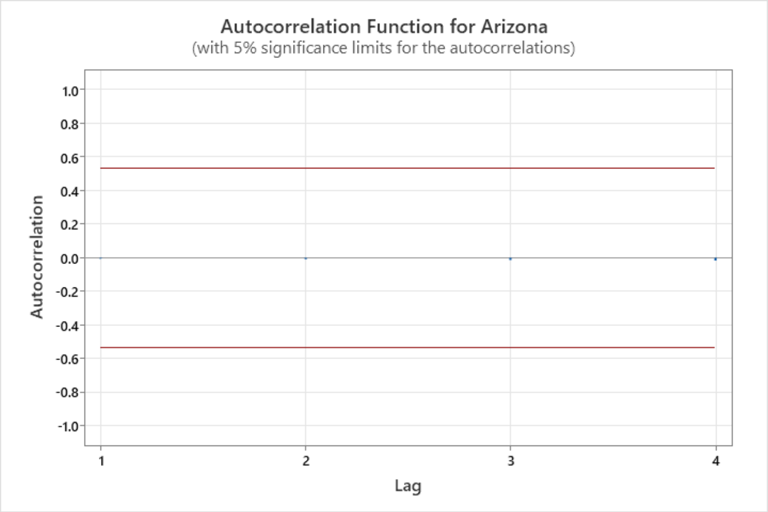

Fig. S8. Autocorrelation function (ACF) plot of raw milk outbreaks for Arizona.

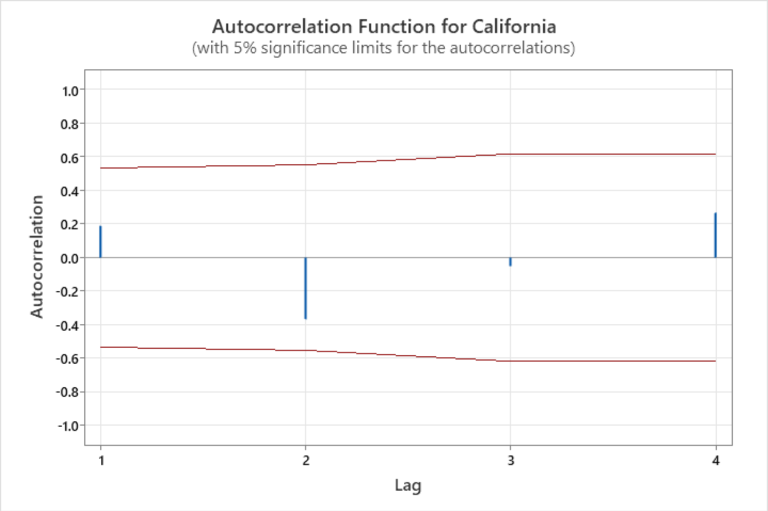

Fig. S9. Autocorrelation function (ACF) plot of raw milk outbreaks for California.

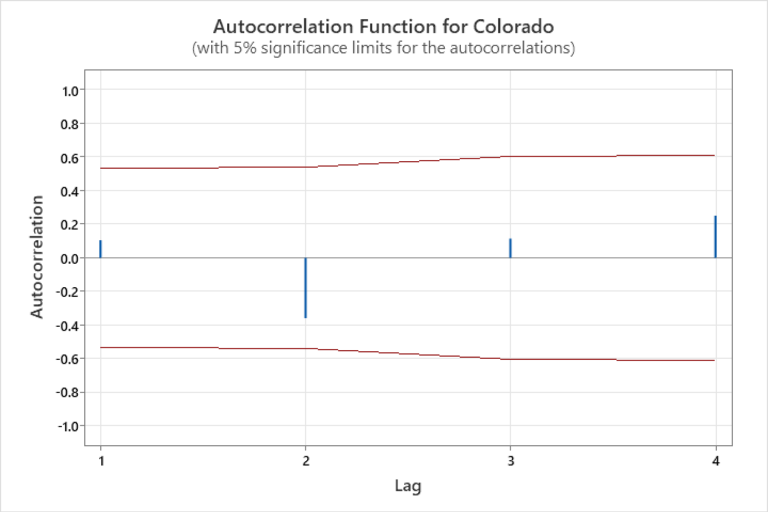

Fig. S10. Autocorrelation function (ACF) plot of raw milk outbreaks for Colorado.

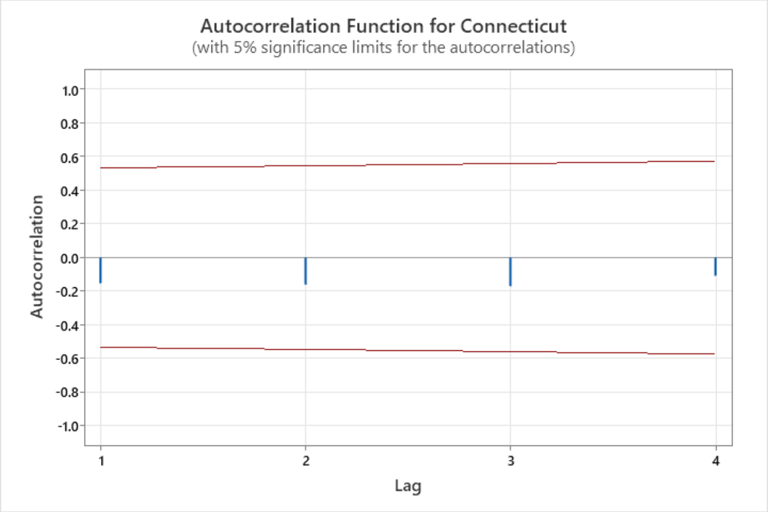

Fig. S11. Autocorrelation function (ACF) plot of raw milk outbreaks for Connecticut.

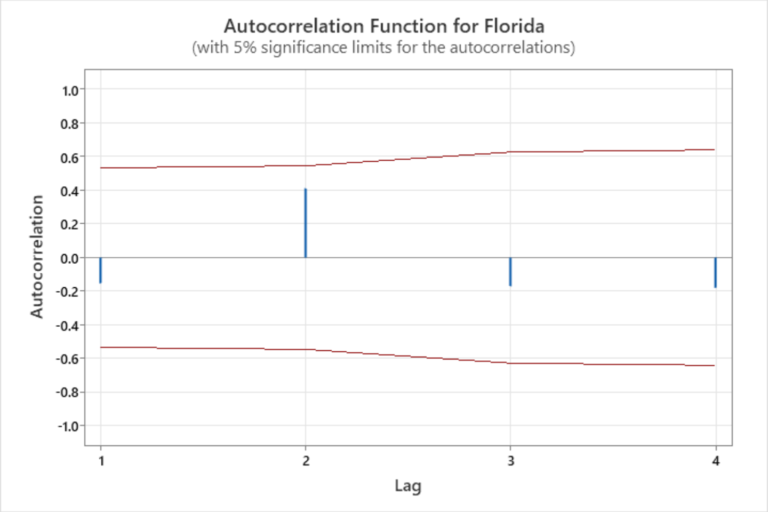

Fig. S12. Autocorrelation function (ACF) plot of raw milk outbreaks for Florida.

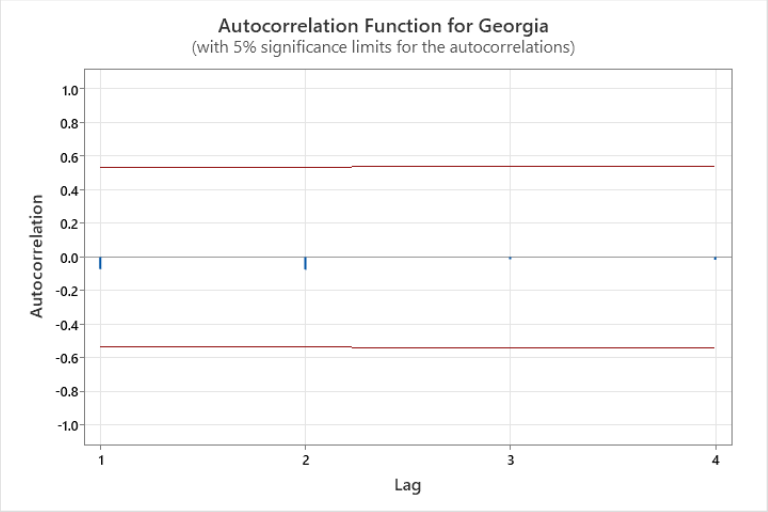

Fig. S13. Autocorrelation function (ACF) plot of raw milk outbreaks for Georgia.

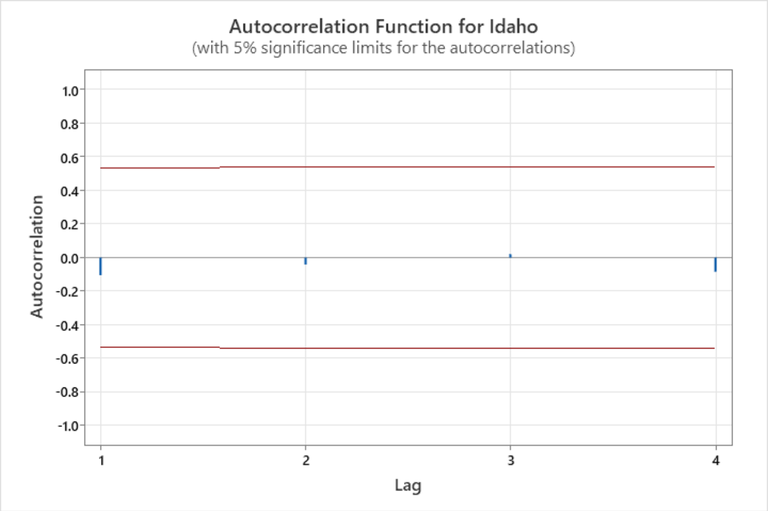

Fig. S14. Autocorrelation function (ACF) plot of raw milk outbreaks for Idaho.

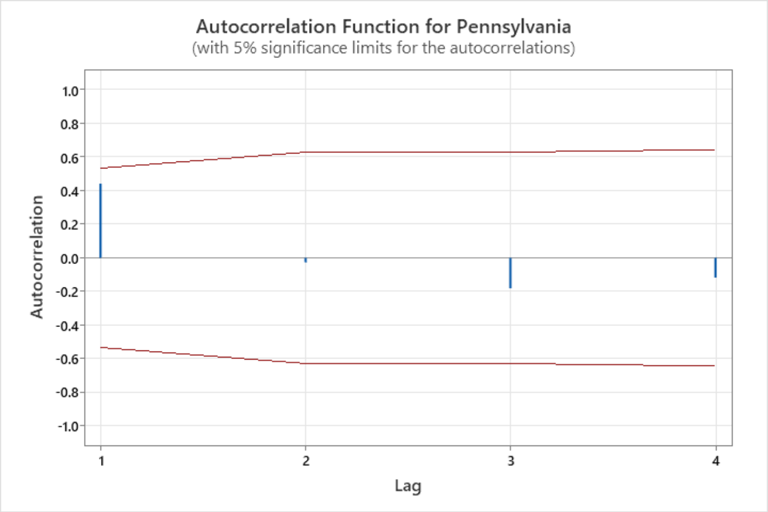

Fig. S15. Autocorrelation function (ACF) plot of raw milk outbreaks for Pennsylvania.
